# A human breast cancer-derived xenograft and organoid platform for drug discovery and precision oncology

**DOI:** 10.1038/s43018-022-00337-6

**Published:** 2022-02-24

**Authors:** Katrin P. Guillen, Maihi Fujita, Andrew J. Butterfield, Sandra D. Scherer, Matthew H. Bailey, Zhengtao Chu, Yoko S. DeRose, Ling Zhao, Emilio Cortes-Sanchez, Chieh-Hsiang Yang, Jennifer Toner, Guoying Wang, Yi Qiao, Xiaomeng Huang, Jeffery A. Greenland, Jeffery M. Vahrenkamp, David H. Lum, Rachel E. Factor, Edward W. Nelson, Cindy B. Matsen, Jane M. Poretta, Regina Rosenthal, Anna C. Beck, Saundra S. Buys, Christos Vaklavas, John H. Ward, Randy L. Jensen, Kevin B. Jones, Zheqi Li, Steffi Oesterreich, Lacey E. Dobrolecki, Satya S. Pathi, Xing Yi Woo, Kristofer C. Berrett, Mark E. Wadsworth, Jeffrey H. Chuang, Michael T. Lewis, Gabor T. Marth, Jason Gertz, Katherine E. Varley, Bryan E. Welm, Alana L. Welm

**Affiliations:** 1 Department of Oncological Sciences, University of Utah, Salt Lake City, UT USA; 2 Huntsman Cancer Institute, University of Utah, Salt Lake City, UT USA; 3 Eccles Institute of Human Genetics, University of Utah, Salt Lake City, UT USA; 4 Department of Pathology, University of Utah, Salt Lake City, UT USA; 5 Department of Surgery, University of Utah, Salt Lake City, UT USA; 6 Department of Internal Medicine, Division of Medical Oncology, University of Utah, Salt Lake City, UT USA; 7 Department of Neurosurgery, University of Utah, Salt Lake City, UT USA; 8 Department of Orthopaedics, University of Utah, Salt Lake City, UT USA; 9 Department of Pharmacology and Chemical Biology, University of Pittsburgh, UPMC Hillman Cancer Center, Magee Womens Research Institute, Pittsburgh, PA USA; 10 Lester and Sue Smith Breast Center, Baylor College of Medicine, Houston, TX USA; 11 The Jackson Laboratory for Genomic Medicine, Farmington, CT USA; 12 Department of Genetics and Genome Sciences, UCONN-Health, Farmington, CT USA

**Keywords:** Breast cancer, Cancer models, Cancer therapy, Cancer

## Abstract

Models that recapitulate the complexity of human tumors are urgently needed to develop more effective cancer therapies. We report a bank of human patient-derived xenografts (PDXs) and matched organoid cultures from tumors that represent the greatest unmet need: endocrine-resistant, treatment-refractory and metastatic breast cancers. We leverage matched PDXs and PDX-derived organoids (PDxO) for drug screening that is feasible and cost-effective with in vivo validation. Moreover, we demonstrate the feasibility of using these models for precision oncology in real time with clinical care in a case of triple-negative breast cancer (TNBC) with early metastatic recurrence. Our results uncovered a Food and Drug Administration (FDA)-approved drug with high efficacy against the models. Treatment with this therapy resulted in a complete response for the individual and a progression-free survival (PFS) period more than three times longer than their previous therapies. This work provides valuable methods and resources for functional precision medicine and drug development for human breast cancer.

## Abstract (web summary)

Welm and colleagues present a biobank of human-derived xenografts and organoids and demonstrate its value for high-throughput drug screening and applied precision medicine.

## Main

The heterogeneity of human cancers has limited success of drug treatment. Models recapitulating the reality of variable treatment responses are needed for more precise drug development. To model human tumors, we and others have developed and exploited PDX models, whereby human tumors are implanted into immune-deficient mice and serially transplanted. PDX models recapitulate human tumors with high fidelity^1^ and exhibit treatment responses that are concordant with human responses^2^. PDX models can also be used to interrogate drug response and resistance, study tumor heterogeneity and evolution, and model metastatic disease^2^. However, whether used for precision oncology or as research tools, PDX models are limited by high cost and low throughput. For several solid tumors, three-dimensional (3D) organoid modeling from human tumors and PDXs is now feasible and is more representative of human cancer than two-dimensional (2D) cultures^3^. Human patient-derived organoids (PDOs) show strong biological fidelity with their parental tumors, including concordant drug responses, and have been developed for many cancer types^4^.

Genomic testing is becoming mainstream to personalize cancer therapy. In a study of 429 individuals with diverse malignancies, 62% had mutations that matched to at least one drug, and 20% had mutations that matched to multiple drugs. Compared to the 38% of individuals who received physician’s choice of drug (unmatched or low-match cases), individuals who received all matching drugs had longer PFS^5^. However, accumulating data suggest that functional testing using human-derived models may hold distinct advantages over genomics alone to personalize therapy. In a study of 769 individuals with various cancers, genomics identified therapeutic options for <10% of individuals with advanced disease, with a <1% success rate for a match to an approved therapy^6^. However, organoids or PDXs were grown from 38% of cases. As a proof of concept, models from four cases were tested with combined genomic and functional testing, and in all cases, effective targeted agents and combinations were identified. Although drug responses could often be related to genomic findings, in half of the cases, functional screening identified different drug responses despite similar driver mutations^6^.

It is challenging to identify therapies for breast cancer based on genomic alterations. More genetic and epigenetic drivers are being uncovered, but in metastatic breast cancer, the major medical need, molecular heterogeneity is vast and impedes development of successful therapies^7^. Clinically actionable mutations are identified in 40–46% of cases^8,9^, but no clinical benefit was realized from matching therapies to variants in a recent trial^8^.

Hundreds of PDX models have been developed for breast cancer^10^. However, there remains a shortage of models representing the deadliest breast cancers: drug-resistant, metastatic tumors, endocrine-resistant estrogen receptor-positive (ER^+^) and HER2^+^ tumors. A larger biobank of advanced breast cancer models and in vitro methods to propagate these tumors are necessary to better understand sensitivity and resistance to therapies across diverse breast cancer subtypes. Short-term cultures of human breast cancer cells derived from PDXs can show responses that recapitulate tumor responses in vivo^11,12^; however, long-term cultures are desirable for mechanistic studies of tumor biology and drug response/resistance. The ability to run companion in vivo studies is also ideal. We established a large collection of paired PDX and PDxO models with high fidelity to their original tumors and developed PDxO drug screening techniques. We also demonstrated feasibility for combined genomic and functional precision oncology in the clinical setting.

## Results

### PDX models representing the deadliest forms of breast cancer

We previously reported that breast PDXs recapitulated key tumor characteristics, including metastasis and clinical outcomes^13^. We now emphasize PDXs representing the greatest unmet medical research needs: tumors that are endocrine-resistant, ER^+^ and HER2^+^ coexpressing, unusually aggressive (for example, metaplastic), drug-resistant and primary–metastatic pairs or longitudinal samples from the same individual. A summary of our collection is in Supplementary Table 1 with more detail in Supplementary Table 2.

Our overall ‘take rate’, defined as PDX growth for at least two generations, was 29%. Primary tumors were more difficult to engraft (25% of 102 attempts) than tumors derived from metastases (36% of 50). ER^+^ PDXs were the most difficult to develop, with a take rate of 9% for primary ER^+^ tumors (*n* = 32 attempts) and 16% for metastatic ER^+^ tumors (*n* = 32). Take rates for HER2^+^ primary tumors were 25% (*n* = 8) compared to 33% for HER2^+^ metastatic tumors (*n* = 6). TNBC had a take rate of 58% for primary tumors (*n* = 12 attempts) and 85% for metastases (*n* = 13). While most attempts were from surgical resections or body fluids, such as effusions, we also established PDXs from TNBC primary tumor biopsies. We found take rates for TNBC biopsies to be 29% (*n* = 56 attempts).

Each PDX line was ‘credentialed’ through a rigorous process (Extended Data Fig. 1a), including tests for human and mouse pathogens, such as *Corynebacterium bovis* and lactate dehydrogenase elevating virus (LDEV), and removal of the pathogen if necessary (Methods). PDXs were validated by immunohistochemistry (IHC) to be positive for breast epithelial markers and human mitochondria and negative for mouse and human lymphoma marker CD45 (Supplementary Figs. 1–37). ER, progesterone receptor (PR) and HER2 staining was concordant with the original tumor and/or clinical pathology reports (Supplementary Tables 1 and 2).

**Fig. 1:**
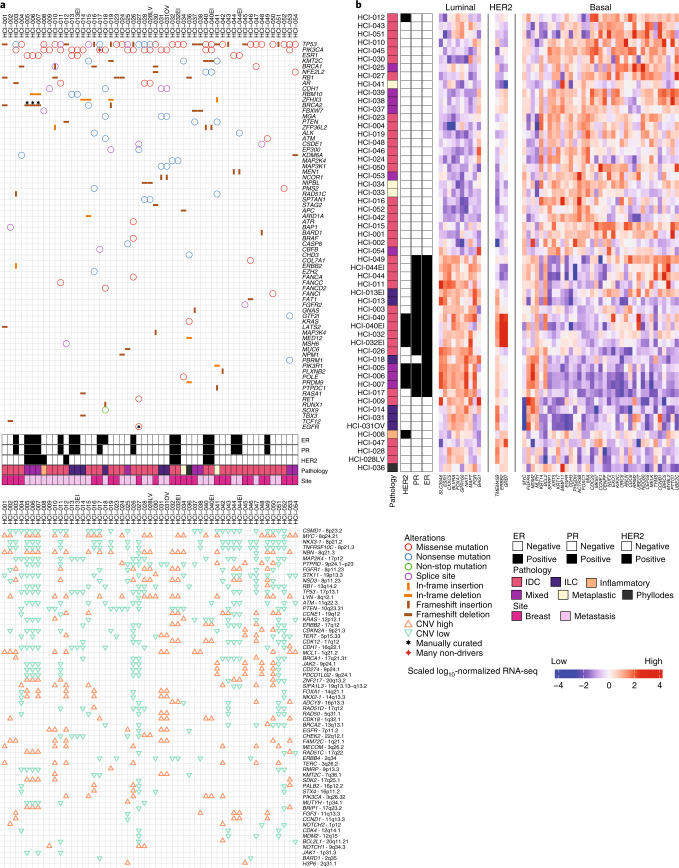
Genomic characterization of breast cancer PDX models. **a**, Top, oncoprint plot showing single-nucleotide variants and insertion–deletions (indels) for commonly mutated genes in cancer. Annotations for each model include hormone receptor (HR) status for ER and PR, HER2 status, pathology (invasive ductal carcinoma (IDC), mixed, phyllodes, invasive lobular carcinoma (ILC), inflammatory or metaplastic) and whether the sample was from the primary breast tissue or a metastatic site. Bottom, specific gene-level CN alterations are displayed. **b**, Unsupervised clustering of the PDX models was performed using root mean squared scaling of transcript abundance in the PAM50 gene set.

**Extended Data Fig. 1:**
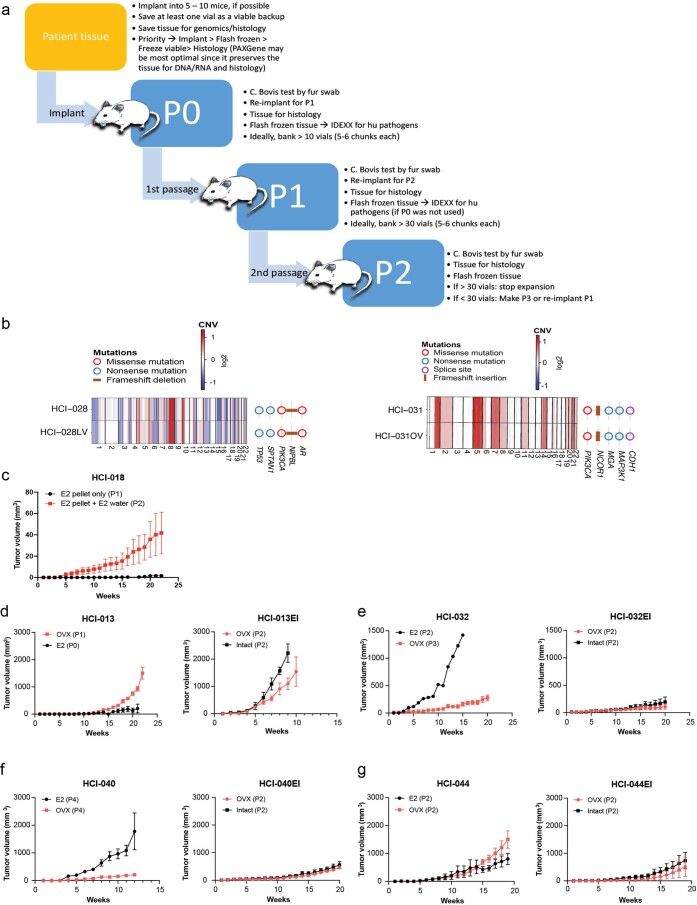
PDX establishment. (**a**) Detailed description of the PDX establishment pipeline **(b)** Side-by-side genomics comparison of WES and CNV are presented for HCI-028 and HCI-028LV **(left)** and HCI-031 and HCI-031OV **(right)**. **(c)** HCI-018 PDX tumor growth in mice implanted with E2 pellets only versus mice that received E2 pellets followed by E2 supplementation in drinking water. Data are shown as mean ± s.e.m. n=5 mice per treatment group. **(d-g)** Establishment of estrogen-independent (EI) ER^+^ PDX sublines. Tumor growth of parental ER^+^ line under standard estrogen supplementation conditions (E2) versus in ovariectomized (OVX) mice with no E2 supplement. Data are shown as mean ± s.e.m. HCI-013 E2 group had 5 mice with two tumors each (n=10 tumors plotted in graph) and HCI-013 OVX group had 5 mice with one tumor each (n=5 tumors plotted in graph); all subsequent mice had 1 tumor each. HCI-032 E2 group had n=1 mouse, HCI-032 OVX group had n=4 mice; HCI-040 E2 group had n=5 mice, HCI-040 OVX group had n=5 mice; HCI-044 E2 group had n=4 mice, HCI-044 OVX group had n=3 mice. Growth of the established EI subline arising from the OVX condition, subsequently transplanted into OVX or intact mice with no E2 supplement. EI PDX lines are maintained in intact mice with no E2 supplement. For HCI-013 only, the tumor from the OVX condition in left panel was first expanded in culture for two weeks in phenol-red free medium with charcoal-stripped serum prior to implantation into OVX mice. Growth in intact mice is the subsequent passage. Data are shown as mean ± s.e.m. HCI-013EI OVX n=5 mice, HCI-013EI Intact n=3 mice per group; HCI-032EI OVX n=5 mice per group, HCI-032EI Intact n=5 mice per group; HCI-040EI OVX n=5 mice per group, HCI-040EI Intact n=5 mice per group; HCI-044EI OVX n=3 mice per group, HCI-044EI Intact n=3 mice per group. ‘P’ denotes passage number. Source data

Genomics analysis revealed that PDXs reflected the heterogeneity of human breast cancer with respect to driver mutations and intrinsic subtypes. We examined missense, nonsense, non-stop, frameshift and splice mutations in known driver genes from previous The Cancer Genome Atlas (TCGA) analyses^14,15^. As expected, the most common variants were in *TP53* and *PIK3CA* (Fig. 1a). We also examined copy number variant (CNV) data using PDXNet standards^1^ and noted common lesions, such as reduced CN for *PTEN* and *RB1* and increased CN for *MYC* (Fig. 1a). RNA-sequencing (RNA-seq) analysis of PAM50 genes^16^ revealed that our collection comprises all the common breast cancer subtypes (Fig. 1b). Analysis of the genomic relationship between PDX lines and their metastatic sublines in mice, when available, was also examined. HCI-028LV (Supplementary Fig. 38) was derived from a metastasis to the mouse liver from the HCI-028 TNBC PDX. HCI-028 was derived from the pleural fluid of an individual who developed liver, bone, ovary and brain metastases. HCI-031OV (Supplementary Fig. 39) was derived from a metastasis to the mouse ovary from the HCI-031 lobular TNBC PDX. HCI-031 was derived from the pleural fluid of an individual that developed metastases in fallopian tubes, bones, pleura, liver and brain. In both cases, the metastatic sublines retained the same genomic driver mutations and have similar gene expression profiles to their parental PDX lines (Fig. 1 and Extended Data Fig. 1b), and both metastatic sublines spontaneously metastasized back to the organ from which they were derived. The metastatic profiles of each PDX model and the corresponding individual, when known, are shown in Supplementary Table 2.

### New models of endocrine-resistant ER^+^ breast cancer

Growth of ER^+^ breast cancers is challenging due to the relatively slow growth rate and dependence on estrogen. Some ER^+^ tumors require supplementation with estradiol (E2)^13^. To avoid urine retention and cystitis caused by estrogen^17^, we lowered the supplemental E2 dose and retained estrogen-dependent growth of ER^+^ PDXs. For extremely estrogen-dependent lines, such as HCI-018, the combination of E2 pellet and E2 maintenance in drinking water was required to sustain tumor growth (Extended Data Fig. 1c). Thus, we established and maintained all standard ER^+^ PDX lines with subcutaneous 0.4-mg E2 pellets placed during tumor implantation, followed by E2 in the drinking water from 4 weeks after implantation to the end of the experiment.

**Extended Data Fig. 2:**
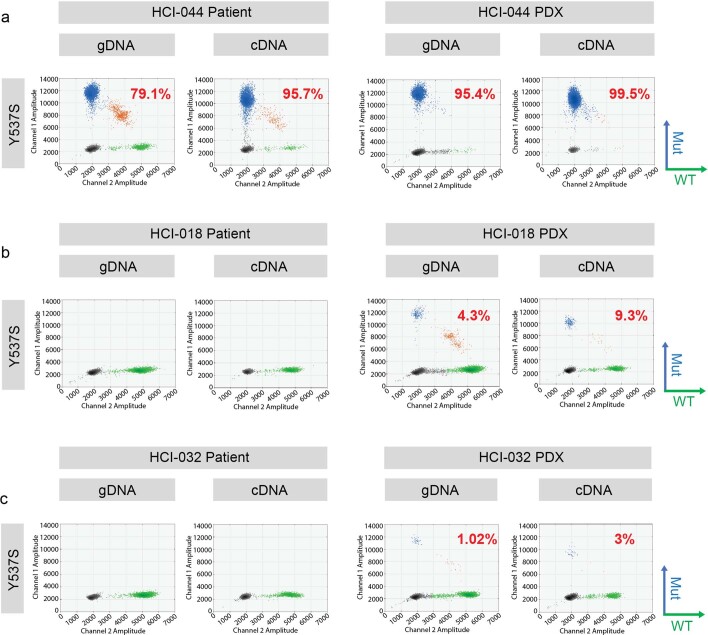
*ESR1* mutation analysis. Droplet digital PCR detection of Y537S homozygous *ESR1* mutation in HCI-044 PDX tumors **(a)** and Y537S low allele frequency tumors in HCI-018 PDX **(b)** and HCI-032 PDX **(c)**. 2D scatter plots of ddPCR results showing fluorescent detection of individual droplets with either gDNA or cDNA. Blue and green dots represent droplets with WT or mutant *ESR1* genotypes indicated on the right panel of each plot, respectively. Orange dots represent droplets containing both WT and mutant *ESR1* DNA. Black dots represent droplets without DNA. Mutation allele frequencies are labelled accordingly.

The estrogen dependence of each ER^+^ PDX was tested by attempting to grow the established PDX in ovariectomized mice with no E2. Tumors that grew in estrogen-deprived conditions were considered E2 independent, and sublines were generated (‘-EI’ designation; Supplementary Table 1, Extended Data Fig. 1d–g and Supplementary Fig. 40). Estrogen independence in metastatic breast cancer has been attributed to mutations in *ESR1* (ref. ^18^), which encodes ERα. We examined the mutation status of *ESR1* in ER^+^ models by droplet digital PCR (ddPCR) for hotspot mutations (Y537S/Y537C/Y537N and D538G). Five of nine (56%) metastatic ER^+^ PDXs from seven individuals contained an *ESR1* mutation (Supplementary Table 1). One model, HCI-044, carries a homozygous Y537S mutation, as does the matching tumor (Extended Data Fig. 2a). In two cases, no *ESR1* mutations were detected in the human sample, but the Y537S mutation was detected at <10% frequency in the PDX (DNA and RNA) samples. These include HCI-018 from a brain metastasis (allele frequency (AF) of 4.3% in DNA and 9.3% in RNA; Extended Data Fig. 2b) and HCI-032 from a recurrent breast tumor (AF of 1% in DNA and 3% in RNA; Extended Data Fig. 2c). The discrepancy between the human sample and PDX is likely due to heterogeneity between the tumor fragments used for the PDX versus that used for DNA/RNA isolation. Heterogeneity of *ESR1* mutations in human samples has been reported along with similar heterogeneity in PDXs^19^. Alternatively, the presence of the mutation in the PDX could reflect evolution of the tumor following engraftment in mice, but the low AF does not suggest strong selective pressure. To this point, selection of the estrogen-independent subline of HCI-032 (HCI-032EI) did not result in any *ESR1* mutations detected by ddPCR.

### Paired PDXs and PDxOs

**Fig. 2:**
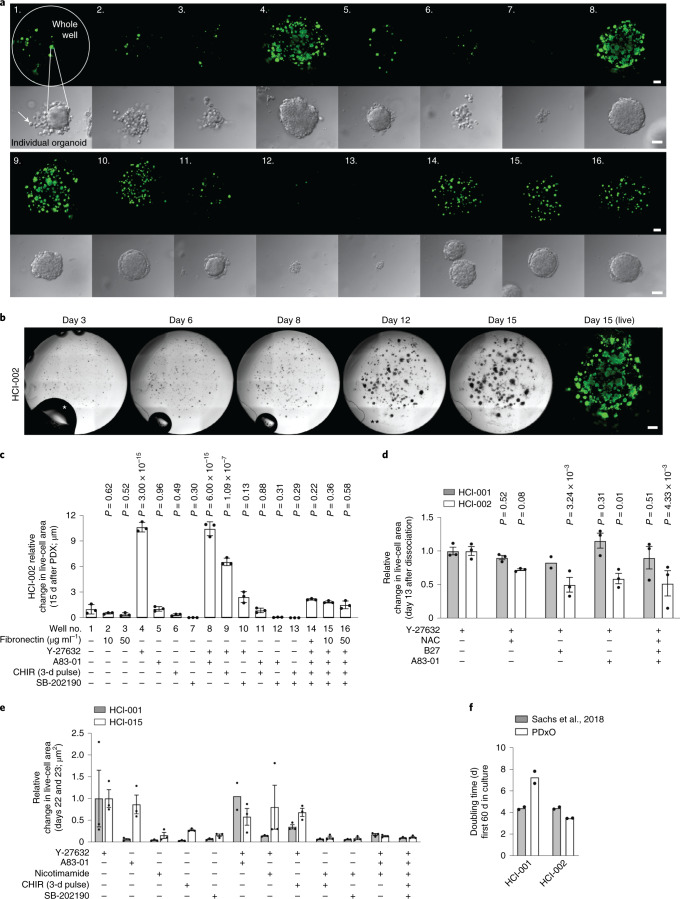
Optimization of PDxO culture conditions. **a**, Live-cell area of entire wells (top) and brightfield images of individual organoids (bottom) representative of PDxO HCI-002 grown under 16 different conditions (experiment in **c**) 15 d after organoid preparation; scale bars, 500 μm (top) and 50 μm (bottom). **b**, Brightfield images representative of organoid growth over time in PDxO HCI-002 (from experiment in **c**). For day 3, the asterisk (*) identifies a bubble in the medium, which gradually disappears during culture. For day 12, the asterisks (**) identify a piece of debris, a common occurrence in the glass-bottom plates required to acquire images; scale bar, 500 μm; right, calcein AM stain to show live cells (green). **c**, Quantified live-cell area of HCI-002 PDxOs grown under 16 different culture conditions. Data are normalized to the control condition (well 1). One experiment was performed. Data are presented as mean ± s.e.m.; *n* = 3 biological replicates. Statistical comparisons to the control condition (well 1) were performed using an ordinary two-way analysis of variance (ANOVA) and uncorrected Fisher’s least significant different (LSD) test with single pooled variance. CHIR, CHIR-99021. **d**, Effect of various culture additives on cell viability 13 d after first dissociation for PDxOs HCI-001 and HCI-002. Data are presented as mean ± s.e.m.; *n* = 3 biological replicates (*n* = 2 biological replicates for HCI-001 Y-27632 + B27). Statistical comparisons to the control condition (+Y-27632) were performed within each line by ordinary two-way ANOVA and uncorrected Fisher’s LSD with single pooled variance. **e**, Effects of culture additives on cell viability for other TNBC PDxO lines, HCI-001 and HCI-015. Data are presented as mean ± s.e.m.; *n* = 3 biological replicates (*n* = 2 biological replicates for HCI-001 Y-27632 + A83-01). **f**, Comparison of doubling times during the first 60 d of culture to previously published organoid growth conditions^20^; *n* = 2 biological replicates. Source data

To generate matched in vivo and in vitro models, we grew long-term organoid cultures from PDXs. To theoretically minimize alterations in the biology of tumors, we optimized conditions that sustained long-term growth of PDxOs with the fewest medium supplements possible. We examined various conditions and assessed PDxO growth by measuring area occupied by live cells in the wells, morphology of organoids and intracellular ATP content. We used HCI-002 as a test model. By testing additives often used in organoid cultures, we found that a Rho kinase inhibitor (Y-27532) was sufficient to support growth of HCI-002 PDxOs over 15 d of initial culture (Fig. 2a–c). Addition of other common supplements did not enhance the effect of Y-27632; some supplements decreased viability (Fig. 2a,c,d). Culturing other TNBC PDxOs under optimized conditions again showed that the critical supplement was Y-27632 (Fig. 2d,e). While some TNBC PDxOs grew slower under our conditions than under conditions previously described by Sachs et al.^20^, others grew at similar rates (Fig. 2f); both methods appeared sufficient to support PDxOs. Because the Sachs medium contains many additives, we chose to use the simpler, Y-27632-supplemented medium as the basal condition for establishment and maintenance of PDxO lines.

**Extended Data Fig. 3:**
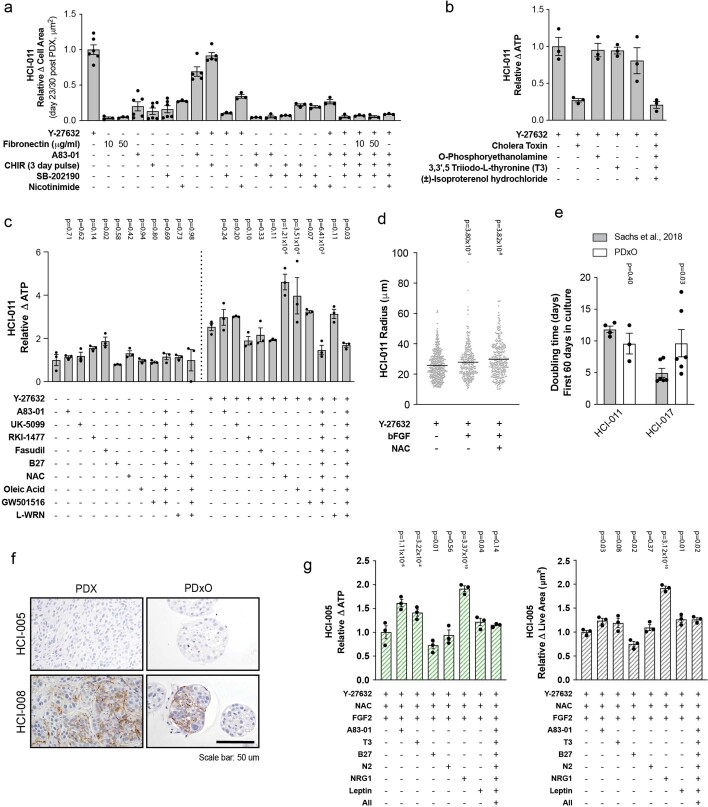
Optimization of ER + and HER2 + PDxO culture. **(a)** Growth response of HCI-011 PDxO to culture medium additives, quantified as live cell area. Mean ± s.e.m. All conditions are n=3 biological replicates except A83-02 + Y-27632 and Y-27632+CHIR (3 days) n=5 biological replicates; Y-27632, A83-01, CHIR (3 days), SB-202190 n=6 biological replicates. **(b)** Effect of other common breast cancer medium supplements on growth of HCI-011 PDxOs, quantified by Cell Titer Glo 3D (CTG-3D) assay to measure ATP content. Mean ± s.e.m., n=3 biological replicates. **(c)** Effect of other common breast cancer medium supplements on growth of HCI-011 PDxOs, quantified by CTG-3D. Mean ± s.e.m., n=3 biological replicates. Statistical comparisons to control condition (minus all (left) or +Y-276362 (right)) by ordinary two-way ANOVA, uncorrected Fisher’s LSD with single pooled variance **(d)** HCI-011 PDxO organoid size (radius) after addition of Y-27632, NAC and FGF2 to PDxO cultures. Each gray dot represents one organoid. Data are shown as mean and individual data points: control n=449 individual organoids, bFGF n=313 individual organoids, bFGF + NAC n=344 individual organoids. Statistical comparisons to control (+Y-27632) by ordinary two-way ANOVA, uncorrected Fisher’s LSD with single pooled variance. **(e)** Comparison of culture doubling time of HCI-011 and HCI-017 when established in our optimized ER + PDxO media or organoid media published by Sachs et al., 2018. Mean ± s.e.m. HCI-017 n=6 biological replicates, HCI-011 Sachs et al n=4 biological replicates, HCI-011 PDxO n=3 biological replicates. Statistical comparisons to control condition (Sachs et al., 2018) within each line by ordinary two-way ANOVA, uncorrected Fisher’s LSD individual variances computed from each comparison. **(f)** Immunohistochemistry of HER2 showing variable HER2 staining in PDxOs and their parental PDX tumor with HER2 + histories. HCI-005 (top) and HCI-008 (bottom) are shown. Each IHC staining has been performed once for each model. Scale bar corresponds to 50 um. **(g)** Effect of additional medium supplements on growth of HCI-005 PDxOs, quantified by CTG-3D assay (left) and live cell area (right). Mean ± s.e.m., n=3 biological replicates. Statistical comparisons to control condition (+Y-27632+NAC + FGF2) by ordinary two-way ANOVA, uncorrected Fisher’s LSD with single pooled variance. Source data

ER^+^ PDxOs displayed good viability when organoids were freshly grown from PDX tumors using the basal conditions but failed to regrow after passaging. To determine additional requirements for ER^+^ PDxOs to thrive long term, we used HCI-011 as a test case (Extended Data Fig. 3a–d). Y-27632 was the most effective Rho kinase inhibitor of the three tested, and most supplements did not outperform Y-27632 alone (Extended Data Fig. 3c, left). In the presence of Y-27632, addition of *N*-acetylcysteine (NAC), oleic acid or basic fibroblast growth factor promoted growth of HCI-011 after dissociation (Extended Data Fig. 3c,d). Oleic acid was not pursued, as it caused accumulation of lipid-filled vacuoles (not shown). Thus, optimal medium for ER^+^ PDxOs comprised the basal medium plus basic fibroblast growth factor and NAC. Again, our optimized conditions and those described by Sachs et al.^20^ both supported long-term growth of ER^+^ PDxOs, although differences in growth rate were observed with some lines (Extended Data Fig. 3e). The significance of in vitro growth rates in terms of pathophysiological relevance to human tumors is unclear, so we again chose the simpler, more cost-effective medium.

**Fig. 3:**
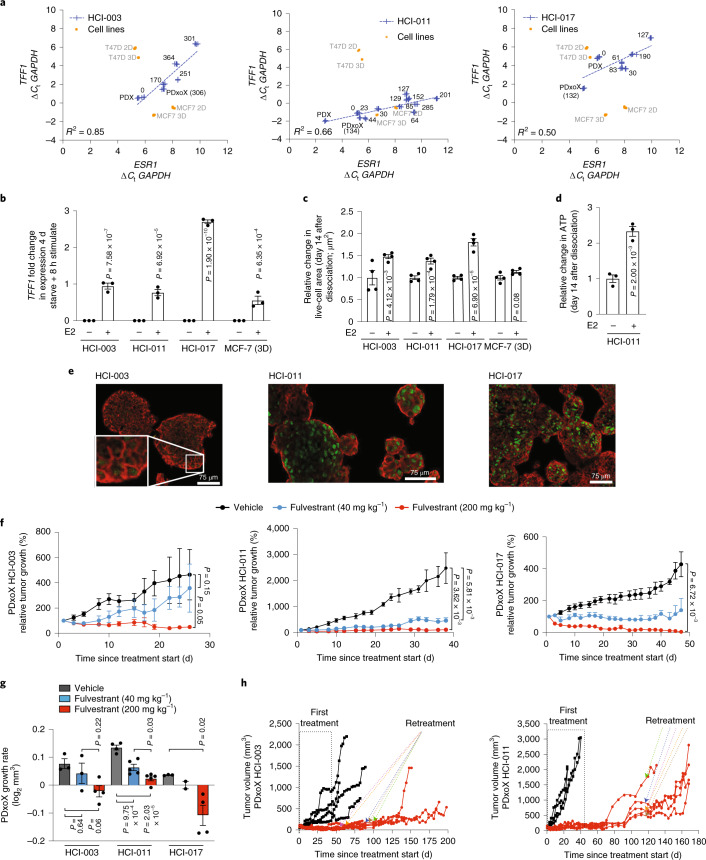
Estrogen pathway integrity in ER^+^ PDxOs. **a**, Expression of *ESR1* and *TFF1* in PDxOs HCI-003, HCI-011 and HCI-017 compared to PDX, PDxoX or MCF7 and T47D cells cultured in 2D or 3D for 6 d. Numbers represent total days in culture. Data are normalized to *GAPDH* and represent *n* = 4 technical replicates; *C*_t_, threshold cycle. **b**, *TFF1* expression in HCI-003, HCI-011 and HCI-017 PDxOs and 3D cultures of MCF7 cells stimulated with E2 for 8 h after 4 d in phenol red-free medium with charcoal-stripped FBS. Data are normalized to *GAPDH* and the no E2 condition. Data are presented as mean ± s.e.m.; *n* = 3 biological replicates. Statistical comparisons within each line were performed using an ordinary two-way ANOVA and uncorrected Fisher’s LSD with individual variances computed from each comparison. **c**, Live-cell area under the same conditions as **b**. Data are normalized to the no E2 condition and are presented as mean ± s.e.m.; *n* = 4 biological replicates. Statistical comparisons within each line were performed using an ordinary two-way ANOVA and uncorrected Fisher’s LSD with single pooled variance. **d**, Quantified ATP of HCI-011 treated as in **b**. Data are normalized to the no E2 condition. Data are presented as mean ± s.e.m.; *n* = 3 biological replicates. A statistical comparison was performed using a two-tailed unpaired *t*-test. **e**, Cytospin immunofluorescence (IF) staining of ER (green) and EpCAM (red) of PDxOs HCI-003, HCI-011 and HCI-017 without E2 stimulation; scale bar, 75 µm. Organoids were stained and imaged once. **f**, HCI-003, HCI-011 and HCI-017 PDxoX tumor response to 40 mg kg^–1^ or 200 mg kg^–1^ fulvestrant treatment. Mean tumor volume relative to tumor volume at treatment start was calculated (HCI-003: vehicle, *n* = 3 mice; fulvestrant (40 mg kg^–1^), *n* = 3 mice; fulvestrant (200 mg kg^–1^), *n* = 4 mice; HCI-011: vehicle, *n* = 4 mice; fulvestrant (40 mg kg^–1^), *n* = 5 mice; fulvestrant (200 mg kg^–1^), *n* = 5 mice; HCI-017: vehicle, *n* = 3 mice; fulvestrant (40 mg kg^–1^), *n* = 2 mice; fulvestrant (200 mg kg^–1^), *n* = 4 mice). Data are presented as mean ± s.e.m.; *P* values were determined by comparing area under growth curves up to 19 d (HCI-003), 24 d (HCI-011) and 47 d (HCI-017) using a two-sided *t*-test. **g**, Tumor growth rate was calculated from the data in **f**. Data are presented as mean ± s.e.m. Statistical comparisons within each line were performed using a one-way ANOVA and Tukey’s multiple comparison test with single pooled variance. **h**, PDxoX HCI-003 (left) and HCI-011 (right) with 200 mg kg^–1^ fulvestrant after off-treatment recurrence to select resistance. Individual tumors are shown, and arrows indicate the start of retreatment for each mouse. Source data

We examined the functionality of ER in ER^+^ PDxO lines in culture. Over time in culture, mRNA levels of *ESR1*, as well as its transcriptional target *TFF1* (ref. ^21^), decreased but remained the same or higher than *ESR1* levels in ER^+^ cell lines grown in 2D or 3D (Fig. 3a). We thus investigated the estrogen responsiveness of long-term ER^+^ PDxO cultures. Our medium includes 5% fetal bovine serum (FBS), which contains E2; we tested whether removing estrogen or supplementing with saturating levels of E2 would affect ER function in PDxO lines. We cultured ER^+^ PDxOs in our regular ER^+^ PDxO medium and compared these conditions to medium containing charcoal-stripped FBS to remove E2. Stripped serum reduced expression of *TFF1*, and *TFF1* expression was rescued 8 h after adding back E2 (Fig. 3b). Survival and growth of PDxOs was also stimulated by E2 (Fig. 3c,d), and the magnitude of E2 response was equal to or better than that of MCF7 cells in 3D culture (Fig. 3b,c).

PDxO cultures retained ER protein expression (Fig. 3e), and three representative ER^+^ PDxO lines cultured long term responded to the selective ER degrader fulvestrant after being engrafted into mice as PDxO xenografts (PDxoX; Fig. 3f,g). These data indicate that long-term ER^+^ PDxO cultures maintain functional ER and retain endocrine sensitivity in vitro and in vivo. Of note, we were able to select for resistance to fulvestrant in the HCI-003 and HCI-011 PDxoX models in vivo by allowing responsive tumors to recur after stopping treatment and then retreating with fulvestrant (Fig. 3h). HCI-017 PDxoXs were more sensitive to fulvestrant; tumors did not recur following initial treatment.

Although we have few HER2^+^ breast cancers in our collection, we have models from individuals with metastatic breast cancer who had HER2^+^ primary tumors but whose tumors lacked HER2 amplification following progression after HER2-targeted therapy (Supplementary Table 1). These models express variable levels of HER2 in the PDX^13^ and PDxO (Extended Data Fig. 3f). We optimized long-term PDxO conditions for these lines; addition of neuregulin 1 significantly boosted growth and metabolic activity of HCI-005, an example of a PDxO with a HER2^+^ history (Extended Data Fig. 3g).

Even with optimized growth conditions for each subtype, some PDxOs were difficult to establish. Certain tumors contained aggressive stroma comprising mouse mesenchymal-like cells that could outcompete the tumor cells. In these cases, mouse cells were removed using fluorescence-activated cell sorting (FACS) for human tumor markers during passaging (Methods). We considered each line to be ‘established’ once it was confirmed to be human breast cancer, free of mouse cells and reliably passageable.

### Long-term PDxOs retain their phenotypes

**Fig. 4:**
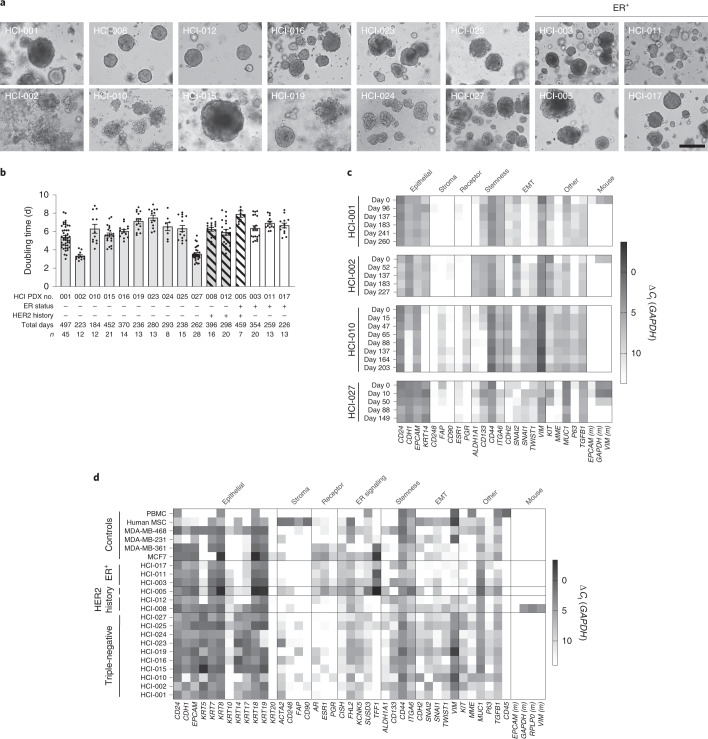
Characterization of established PDxOs. **a**, Brightfield images of established PDxO lines to show characteristic morphology at maturity; scale bar, 50 μm. **b**, Culture doubling times for each established PDxO line over long-term culture. Each dot indicates a culture passage; *n* is indicated below the *x* axis; error bars represent ±s.e.m. **c**, Expression of PDxO characterization genes in HCI-001, HCI-002, HCI-010 and HCI-027 PDxOs at different culture time points. Data are normalized to *GAPDH* and represent technical replicates of *n* = 4; EMT, epithelial-to-mesenchymal transition. **d**, Expression of PDxO characterization genes at single time points for representative PDxOs, validating their human epithelial nature and subtype status, with additional markers to highlight diversity across different lines. Data are normalized to *GAPDH* and represent technical replicates of *n* = 4; PBMC, peripheral blood mononuclear cells; MSC, mesenchymal stem cells. Source data

We derived 40 PDxO lines from 47 attempts (85% take rate) (Supplementary Table 1). These lines have been cultured for >200 d. PDxO lines have diverse morphology and behavior (Fig. 4a). For example, HCI-001 forms cohesive organoids, while HCI-002 grows as dispersive cell clusters; HCI-010 and HCI-019 are morphologically heterogeneous. Generally, PDxOs are solid spheres, with few extruding cells. Doubling times of PDxOs generally ranged between 3 and 8 d, with no notable trends across subtypes (Fig. 4b), although doubling times below 4 d were only observed in TNBC lines.

All PDxOs were validated to be human epithelial cells and were diverse with respect to the expression of other selected genes by quantitative PCR with reverse transcription (RT–qPCR; Fig. 4c,d). For each PDxO line, gene expression across selected panels was consistent over time (Fig. 4c). Exceptions included changes concurrent with loss of mouse cells (Fig. 4c,d) and the unique loss of *CDH1* in HCI-010 (Fig. 4c). HCI-008, an inflammatory breast cancer, was notable for recruitment and retention of stromal cells (Fig. 4d), which had to be removed by FACS.

**Extended Data Fig. 4:**
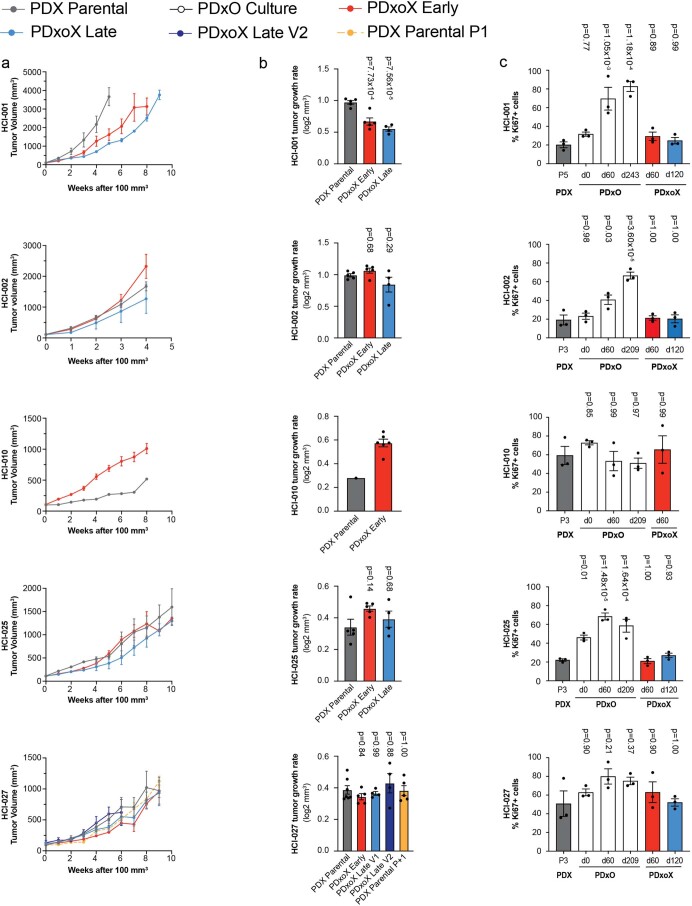
Patient-derived model growth rate and proliferation comparison. PDxO xenograft (PDxoX) tumor volumes **(a)** and tumor growth rates **(b)** compared to parental PDX lines for HCI-001, HCI-002, HCI-010, HCI-025, and HCI-027. PDxOs were in culture for 51-64 days (PDxoX early) or 113-123 days (PDxoX late) prior to xenografting. Tumor volume shown as mean ± s.e.m. Tumor growth rates shown as mean ± s.e.m. HCI-001 parental n=5 mice, early n=5 mice, late n=4 mice; HCI-002 parental n=5 mice, early n=5 mice, late n=4 mice; HCI-010 parental n=1 mouse, early n=6 mice; HCI-025 parental n=5 mice, early n=5 mice, late n=4 mice; HCI-027 parental n=7 mice, early n=5 mice, late n=4 mice, late V2 n=4 mice, PDX parental P1 n=5 mice. Statistical comparisons to control (PDX parental) for growth rates by ordinary one-way ANOVA, Tukey’s multiple comparisons test with a single pooled variance. HCI-010 PDX only generated n=1 mouse, requiring use of two-tailed unpaired t test. **(c)** Quantification of Ki67+ nuclei of parental PDX, early (51-68 days) and late (113-127 days) PDxO cultures and early and late PDxoX. Ki67 staining was performed on one PDX tumor per HCI line, and n=3 individual sets of IHC stains were performed. Data are shown as mean ± s.e.m. and normalized to total hematoxylin+ nuclei count for each image. Statistical comparisons to control (PDX) for Ki67 quantification by ordinary one-way ANOVA, Tukey’s multiple comparisons test with a single pooled variance. Source data

We characterized several PDxO lines in depth to determine if they retained their original characteristics after being propagated long term as organoids. When reimplanted as PDxoXs, tumor growth rates were generally not statistically different from parental PDX growth rates, even when implanted after different time points in culture (Extended Data Fig. 4). Exceptions were HCI-001, which showed a decline in PDxoX growth rate with time in culture, and HCI-010, where the PDxoX derived from an early culture grew faster than the parental PDX (Extended Data Fig. 4). Interestingly, a late culture of HCI-010 failed to grow PDxoX at all, coincident with its phenotypic switch with loss of *CDH1* and gain of *SNAI2l* expression (Fig. 4c). The same result was obtained with an independently generated PDxO line from a different HCI-010 PDX tumor (not shown), suggesting that this phenomenon is intrinsic to the biology of this model.

Although some PDxOs had more Ki67^+^ cells than what was observed in PDXs, the percent of proliferating cells was not different between PDxoXs and PDXs across five lines examined (Extended Data Fig. 4). For PDX tumors with <20% of Ki67^+^ cells, PDxO culture prompted a significant increase in proliferation; however, for PDX tumors with >50% Ki67^+^ cells, Ki67^+^ levels were not significantly altered in culture (Extended Data Fig. 4). Organoid morphology was assessed by hematoxylin and eosin (H&E) staining and IHC with antibodies specific for human-specific vimentin, Ki67 and human-specific cytokeratin 8 (CAM5.2). PDxOs and PDxoXs engrafted after different culture times resembled their originating PDXs (Supplementary Figs. 41–44).

### PDXs and PDxOs retain fidelity to their originating tumors

**Fig. 5:**
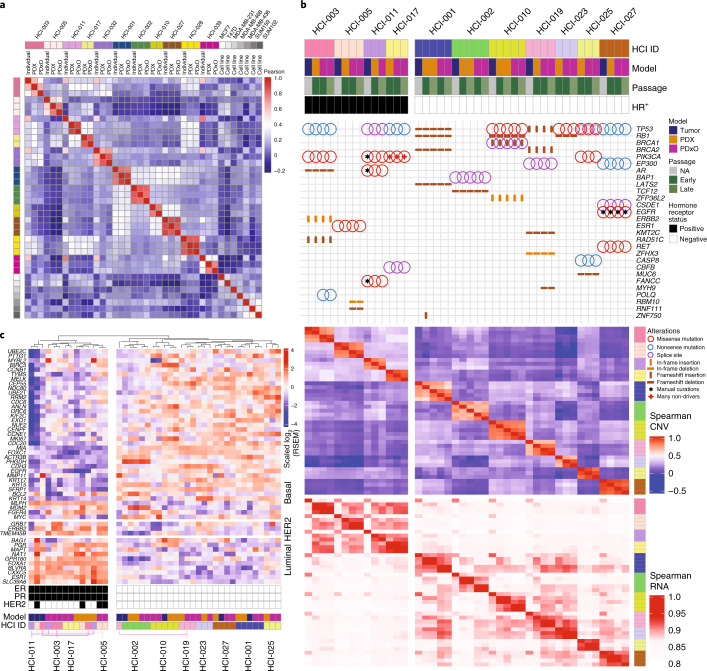
Genomic landscape of PDxOs compared to PDXs and human tumors. **a**, Correlation heat map illustrating genome-wide DNA methylation analysis for 11 sets of patient-derived models compared to commonly used breast cancer cell lines. The color scale indicates the Pearson correlation coefficient. **b**, Eleven sets of models were characterized at different time points (early and late) to assess molecular fidelity with the human tumors. The heat map is divided into four sections from top to bottom: annotations, exome sequencing variant detection, CN correlations from SNP array data and RNA-seq gene expression correlations. Mutation variants are shown with an oncoprint plot highlighting single-nucleotide variants and indels for commonly mutated genes in breast cancer. Quantitative CNV correlations are shown using a heat map of Spearman correlations for gene-level log_2_ CN ratios. Quantitative transcriptome correlations are shown using a heat map of Spearman correlations for gene-level log_10_-transformed RNA-Seq by Expectation-Maximization (RSEM) count estimates; NA, not applicable. **c**, Unsupervised clustering of the same models shown in **b**, with the PAM50 gene set to classify subtype.

Breast tumors have specific patterns of DNA methylation, which distinguish cancers from normal tissue or benign tumors and reflect breast cancer subtype and features, such as metastatic potential^22,23^. As an initial comparison between our models and the tumors from which they were derived, we performed a genome-wide DNA methylation analysis on nine sets of matched human tumors, PDXs and PDxOs as well as two PDX–PDxO pairs for which primary tumor material was not available. These data revealed that the human-derived models are more similar to their originating tumors than are commonly used cell lines (Fig. 5a).

Further genomics analysis on models for which we had both early- and late-passage matched PDXs and PDxOs (11 sets of ER^+^ and TNBC models) revealed high concordance between individual samples, PDX and PDxO models (Fig. 5b,c). While common driver mutations (for example, in *TP53*, *RB1*, *BRCA1* and *PIK3CA*) were retained in all early- and late-passage models, unique variations in five cancer-associated genes were observed in 4 of the 11 sets of models. The model-specific variations included a *POLQ* nonsense mutation only in PDxOs derived from HCI-003 and *RBM10* and *RNF111* deletions only in PDxOs derived from HCI-005. We also found a *ZNF750* in-frame insertion in early-passage HCI-001 PDXs but not in later-passage PDXs nor in early- or late-passage PDxOs and a frameshift deletion of *MYH9* that was observed in HCI-019 PDxOs but not in the PDXs or human tumor. In all cases, the PDxOs were made from a different passage of the PDX than that which was sequenced. Thus, mutational differences could be due to subclone selection bias from passaging heterogeneous tumors, or due to a small degree of tumor evolution. However, these data show that, overall, the major mutational drivers observed in human tumors were maintained in models at various passages.

**Extended Data Fig. 5:**
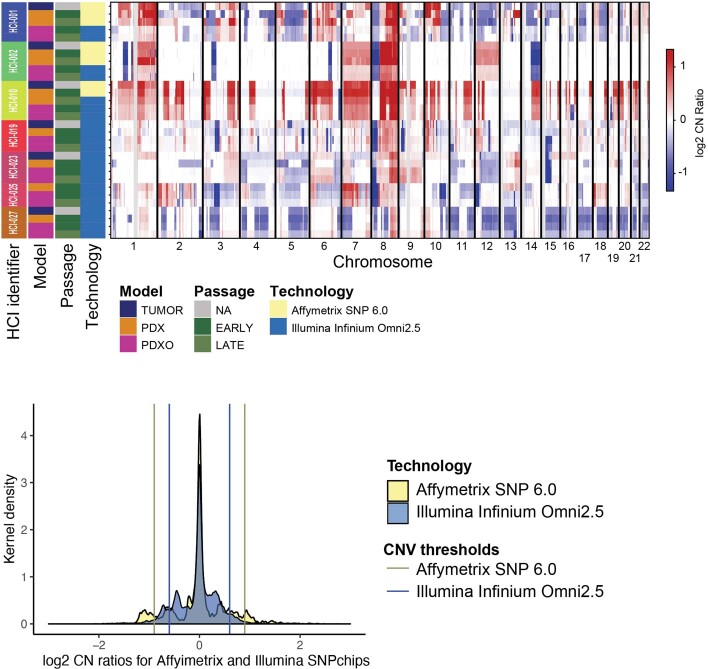
CNV analysis. CNVkit segmentented copy numbers plot113 illustrates log2 CN ratios for the indicated models. The annotations (left) display HCI-line, model, passage, technology (i.e., SNPchip platform). Segmented copy number data is presented as log2 CN ratios indication amplifications (red) and deletions (blue). (Lower) Graph showing density plot of log2 CN ratios are colored according to the sequencing platform. Vertical bars indicate the thresholds set to define discrete amplifications and deletions.

Systematic, engraftment-specific CN changes have been reported in PDX models^24^; however, a recent comprehensive study found that CN changes are minimal and largely attributed to spatial heterogeneity of samples rather than to tumor evolution following engraftment^1^. To examine whether gross CN changes occurred in our models following in vivo and ex vivo passaging, we assessed early- and late-passage models versus their matching human tumors using single-nucleotide poymorphism (SNP) arrays. We observed high correlation between human samples and their models compared to models from different individuals (*P* = 9.14 × 10^–42^ and 95% confidence interval (95% CI) = 0.57 to 0.63; Mann–Whitney *U*-test of Spearman correlation values; Fig. 5b and Extended Data Fig. 5). This result supports gross CN conservation from human samples to their models^1^. At the gene level, we only observed a few genes that transitioned CN following model passaging. One example is *SNCG* (10q23.2) in HCI-001. Interestingly, *SNCG* was the only gene of 30 in the region that transitioned from amplified to deleted across passages (CN ratios = 0.99, −0.56, −0.66, −1.47 and −1.51 for human tumors, PDX-early, PDX-late, PDxO-early and PDxO-late, respectively). Five genes at 6q27 exhibiting large genomic changes (*PSMB1*, *PDCD2*, *TBP*, *OR4F7P* and *WBP1LP8*) were found in HCI-010 as the region transitioned from amplified in the human tumor to deleted in the early passage of the PDX and PDxO (Extended Data Fig. 5). Thus, while some CN alterations may reflect selection in the PDX, our data suggest that xenografts and organoids generally retain the genomic structure of the human tumors.

Analysis of the whole transcriptome also showed high correlation of transcript abundance between samples (0.86 ± 0.023 s.d., Spearman correlation coefficient). We observed that intramodel correlation was significantly greater than intermodel correlations for each model (*P* = 7.75 × 10^–29^, Mann–Whitney *U*-test of Spearman correlation values; Fig. 5b). We noted less similarity between human tumors and models in ER^+^ cases, suggesting that ER^+^ models may have more selective pressure (*P* = 0.0008, 95% CI of −0.03 to −0.01; Mann–Whitney *U*-test of Spearman correlation values). This is consistent with low ‘take rates’ of ER^+^ PDXs. However, ER^+^ PDXs and PDxOs retained the luminal subtype of their parental tumors (Fig. 5c).

### PDxO drug responses are concordant with responses in vivo

**Fig. 6:**
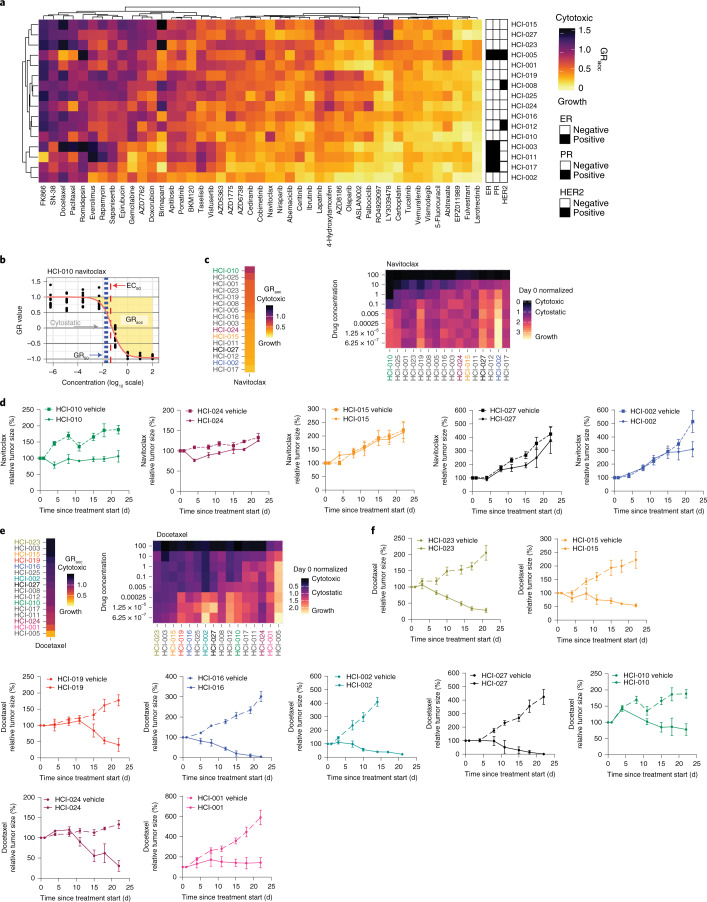
PDxO drug screening shows concordance with in vivo data and identifies birinapant as a potential therapy for some TNBC tumors. **a**, Unsupervised clustering of 16 PDxO models and 45 screened compounds. Color indicates GR_aoc_ statistics (darker colors indicate cytotoxicity, and lighter colors indicate growth). Annotations indicate HR status. **b**, An illustration of dose–response curve statistics that can be calculated using the R package GRmetrics. The *y* axis displays growth rate-adjusted estimates from the CellTiter-Glo 3D (CTG-3D) cell viability assays. The *x* axis shows log fold change of eight-point dose concentrations. Each dot represents 1 of 12 replicates (3 biological replicates and 4 technical replicates each). Annotations include half-maximal effective concentration (EC_50_), GR_50_ (concentration at which the GR value is 0.5), cytostatic (concentration at which the model is neither growing nor shrinking) and GR_aoc_ (the area over the dose–response curve that estimates both sensitivity and cytotoxicity). **c**, Ordered models based on GR_aoc_ for navitoclax sensitivity. High values with darker colors suggest a cytotoxic response to the compound. Drug concentrations are micromolar units. The colors of the model identifiers correspond to in vivo data in **d**. The heat map displays drug response to navitoclax in PDxO screens. The coloration indicates CTG-3D cell viability assays in PDxO screens that were normalized to day 0 ranging from 0 (black, cytotoxic) to 3 (yellow, growth). Models are sorted by GR_aoc_ estimate. **d**, Responses to navitoclax in vivo for the most sensitive model predicted by PDxO screening (HCI-010) and four others: HCI-024, HCI-015, HCI-002 and HCI-027. Data are shown as mean ± s.e.m. Treatment groups for all PDX lines are composed of *n* = 3 mice; vehicle groups for all PDX lines include *n* = 6 mice. **e**, Stacked heat map displays ordered GR_aoc_ calculations for each model’s response to docetaxel from dark (cytotoxic) to light (growth). The colors of model identifiers correspond to in vivo modeling in **f**. The heat map displays drug response to docetaxel in PDxO screens. Drug concentrations are micromolar units. The coloration indicates CTG-3D cell viability assays in PDxO screens that were normalized to day 0 ranging from 0 (black, cytotoxic) to 2.5 (yellow, growth). Models are sorted by GR_aoc_ estimate. **f**, Results of in vivo docetaxel treatment for HCI-023, HCI-015, HCI-019, HCI-016, HCI-002, HCI-027, HCI-010, HCI-024 and HCI-001. Data are shown as mean ± s.e.m. Treatment groups for all PDX lines include *n* = 3 mice; vehicle groups for all PDX lines include *n* = 6 mice. Source data

**Extended Data Fig. 6:**
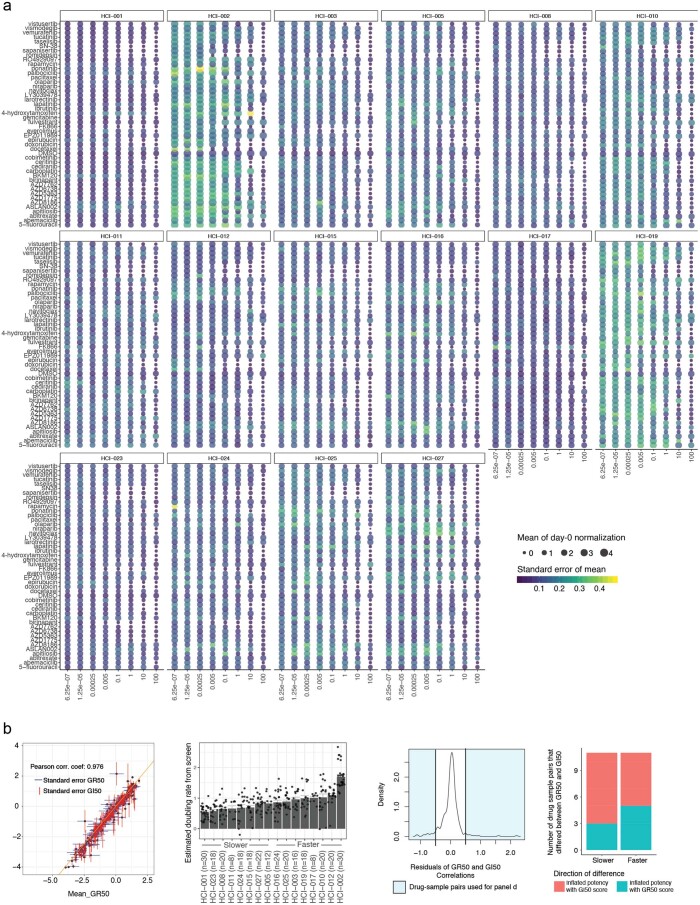
Drug screen quality control. **(a)** Sixteen bubble plots display variability of the screening data across replicates. Plotted are the mean (size) and standard error of the mean (color) for each drug concentration (x-axis) and drug (y-axis) pair using day-0 normalized values. Lighter colors indicate the most variable measures. **(b, left)** Correlation plot showing mean GI50 scores versus GR50 scores as different data normalization techniques for sample-to-sample comparisons. Each point displays the mean log-transformed GR50 and mean log-transformed GI50 values across biological replicates (all replicates required GR50 and GI50 estimates) with extending whiskers (1 standard error) for drug-sample pairs. **(b, mid left)** Estimated growth rates from the screening data are shown as mean values (slower to faster growing models from left to right). Error bars indicate + /- s.e.m. Models are grouped into faster and slower growers and compared in panel d. Point indicates the estimated double rate of each biological replicate using log2 ratio of endpoint of DMSO treated organoids and day zero measurements. Number of samples is indicated on x-axis labels. **(b, mid right)** Density figure displays the residuals, i.e., the biggest differences from a perfect correlation. Shaded in light blue are the data with the biggest discrepancies between the two drug response metrics. The shaded area includes 32 drug-sample pairs that are analyzed in panel d. **(b, right)** Using the most discrepant samples from panel b, we display where GR50 or GI50 disproportionally inflate potency metrics in faster and slower growers. Here we show that GR50 and GI50 metrics do not inflate or deflate drug responses based on variable growth rates in these models (16 drug-sample pairs in faster, and 16 drug sample pairs in slower group). Stacked bars are colored by the direction of discrepancy, i.e., GR50 scores less than GI50 scores (teal) or GI50 scores were less than GR50 scores (red).

**Extended Data Fig. 7:**
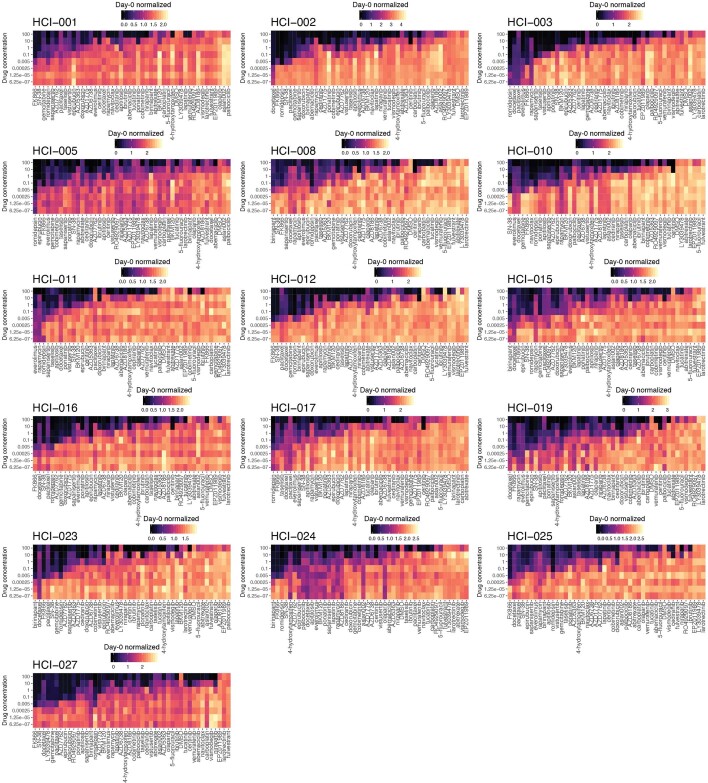
Drug screen data sorted by HCI line. Sixteen heat maps, organized by model, illustrate individual drug responses to 45 compounds. Coloration of these heatmaps indicates CellTiter-Glo 3D cell viability assays that were normalized to day 0, ranging from 0 (black, cytotoxic) to 2 + (yellow, growth). Values at 1 are considered cytostatic. Color scaling is performed relative to each model. Drugs are sorted left to right from largest GRaoc to smallest, indicating decreasing drug efficacy in each model, respectively. Drug concentrations are micromolar units.

**Extended Data Fig. 8:**
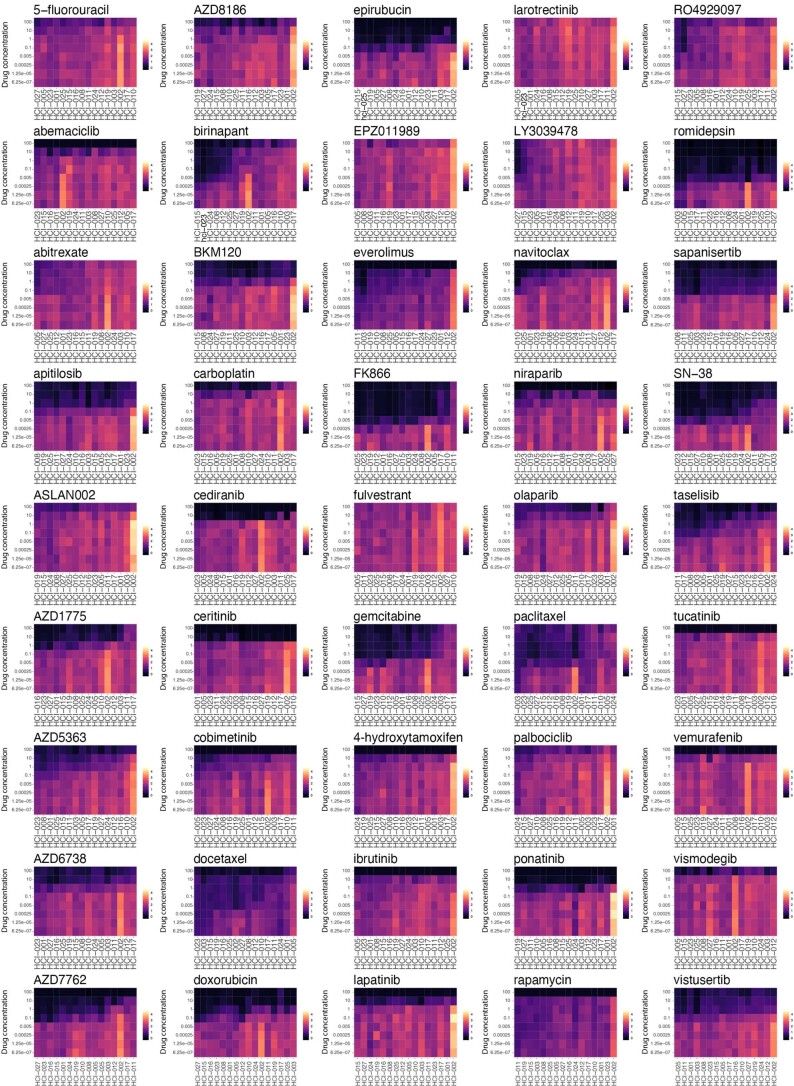
Drug screen data sorted by compound. Forty-five tiled heatmaps, organized by drug, display day-zero normalized values at multiple concentrations for each drug (darker colors indicated cytotoxic responses while lighter colors indicate growth). Coloration scales are identical from figure to figure. Samples are sorted from left to right by AOC metrics (x-axis). Drug concentrations are micromolar units.

We developed drug screening protocols for PDxOs, whereby organoids were plated into a 384-well format for testing in a 4-d drug response assay. We used a large dose range of each compound, so the entire range of effects (growth, cytostasis or cytotoxicity) could be observed in each PDxO line with each drug in a relatively high-throughput manner. Sixteen PDxO lines were screened against a panel of 45 compounds in an eight-point dose–response assay with technical quadruplicates and in biological triplicate to determine therapeutic response. Reproducibility was robust across replicates (Extended Data Fig. 6). Due to variable doubling times between models, we used the growth rate-correcting strategy proposed by Hafner et al. to calculate and score our dose–response curves^25^. This method adjusts cell viability using a log_2_ fold change estimator and allows us to compare drug responses between models from different individuals even when growth rates were different. We found that GR_50_, the concentration at which we model half-maximal adjusted growth, and GR_aoc_, the area over the dose–response curve, are suitable estimations for organoid drug sensitivity and cytotoxicity. The landscape of treatment responses across different PDxO lines is shown in Fig. 6a and Extended Data Figs. 7 and 8.

**Extended Data Fig. 9:**
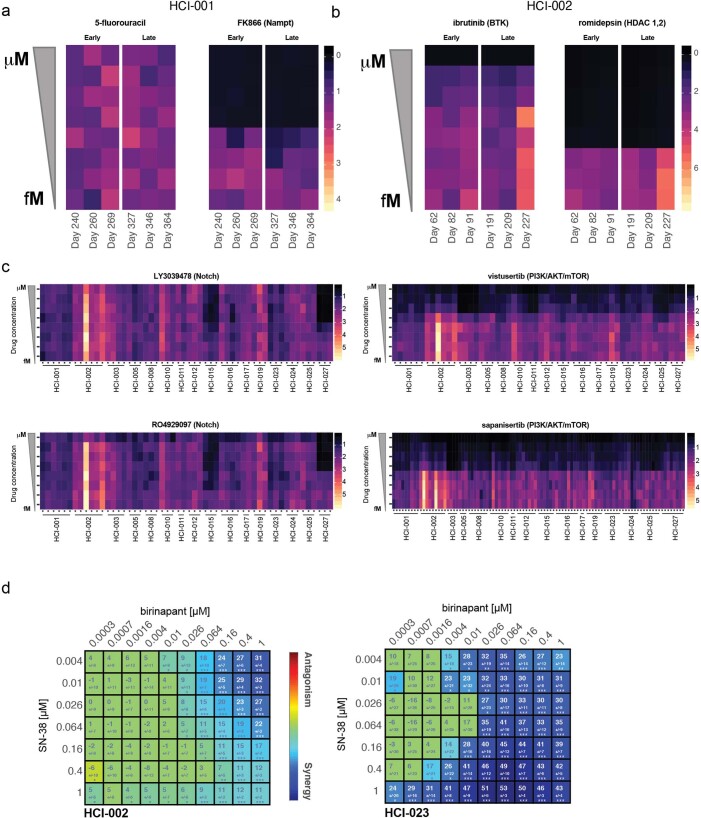
Longitudinal drug screens and synergy. **(a**) Two compounds, 5-fluorouracil and FK866 were screened at eight-point dose response curves (y-axis) at different days in culture (x-axis). Individual replicates and dose responses from a CellTiter-Glo 3D cell viability assay were normalized to day 0 for the model HCI-001. Values range from 0 (black) to 4 (light-yellow), where zero indicates cytotoxic effects and yellow shows growth phenotypes. Values at 1 are considered cytostatic. **(b)** As in the previous panel two compounds, ibrutinib and romidepsin, were screened in model HCI-002 at different times in culture. Here, response values range from 0 (red) to 7 (light-yellow). Values at 1 are considered cytostatic. **(c)** Individual biological replicates for 16 models are shown for two different targeted therapies in the Notch pathway (LY3039478 and RO4929097), as well as two targeted therapies in the mTOR pathway (vistuserib and sapanisertib). Colored as previously described in panel **(a)**. Response values range from 0 (black) to 5 (light-yellow). Values at 1 are considered cytostatic. **(d)** Synergy plot as a result of drug treatment of birinapant and SN-38 in HCI-002 (left) and HCI-023 PDxOs (right). Blue indicates synergy and red indicates antagonism. The number in the box represents the Lowe synergy score + /- standard deviation as provided by Combenefit software; Screen was performed in n=12 replicates as defined in methods section. Statistical differences are reported by the software as one sample t-test and *p < .05, **p < .001, ***p < .0001. Source data

PDxO drug responses were reproducible when the screens were repeated multiple times over up to 1 year in culture, and similar responses were achieved with different compounds against the same target (Extended Data Fig. 9). Using GR_aoc_ scores (Fig. 6b), each model can be ranked for sensitivity to each drug. Examples are shown for the BCL2 antagonist navitoclax, where HCI-010 was the most responsive PDxO line (Fig. 6c) and was the most responsive PDX tested (Fig. 6d). Similar results comparing organoid and PDX responses to docetaxel are shown in Fig. 6e,f.

**Fig. 7:**
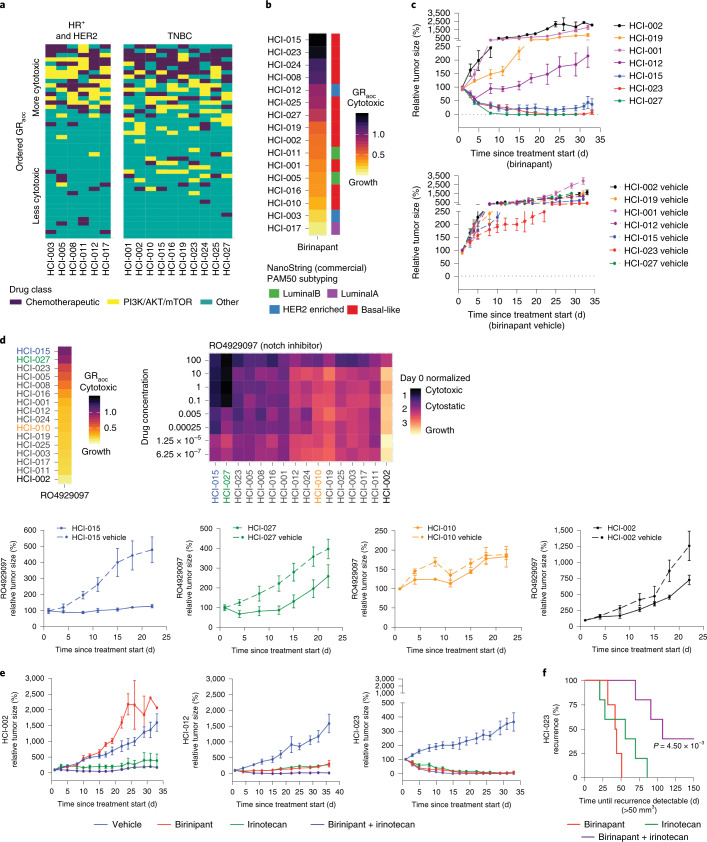
Growth rate-adjusted PDxO screening analysis ranks models in concordance with PDX response. **a**, A tile plot displays sample-specific drug ranks colored by drug class: chemotherapeutic agents (dark purple), PI3K/AKT/mTOR-targeted agents (yellow) and all other drugs (teal). Samples are separated by HR^+^ and HER2^+^ tumors or TNBC. **b**, Stacked heat map rank of PDxO models for birinapant drug responses. Samples are sorted by GR_aoc_ with the best responses on top. The PAM50 breast cancer subtype for each model is displayed to the right. **c**, In vivo drug treatment response to birinapant in various PDX models (top) with matching vehicle controls (bottom). Data are shown as mean ± s.e.m.; birinapant treatment groups: *n* = 5 mice for all PDX lines; vehicle groups: *n* = 5 mice for all PDX lines. **d**, Stacked heat map displays GR_aoc_ calculations for each model’s response to the γ-secretase inhibitor RO4929097 from dark (cytotoxic) to light (growth). The color of the model identifiers corresponds to in vivo modeling within **d**. The heat map displays drug response to RO4929097 in PDxO screens. The coloration indicates CTG-3D cell viability assays in PDxO screens that were normalized to day 0 ranging from 0 (black, cytotoxic) to 2 (yellow, growth). Models are sorted by GR_aoc_. Drug concentrations are micromolar units. **e**, In vivo drug treatment response to birinapant, irinotecan or a combination in HCI-002 (left), HCI-012 (middle) and HCI-023 (right) PDX models. Data are shown as mean ± s.e.m.; treatment and vehicle groups: *n* = 5 mice for all PDX lines. **f**, Time to recurrence of HCI-023 PDX tumors following cessation of birinapant, irinotecan or combination treatment compared by a log-rank Mantel–Cox test; treatment groups: *n* = 5 mice for all PDX lines. Source data

As expected, certain drugs showed selective effects on particular breast cancer subtypes. Cytotoxic chemotherapies showed more activity against TNBC lines than non-TNBC lines (*P* = 0.04334, Mann–Whitney *U*-test, comparison of GR_aoc_ scores, 95% CI of 0.004 to 0.235), while PI3K, AKT and mTOR inhibitors showed more activity in ER^+^ and/or HER2^+^ lines than TNBC (*P* = 2.678 × 10^–5^, two-sided Mann–Whitney *U*-test comparison of ranks, 95% CI of 3 to 9 rank positions; Fig. 7a). To investigate further how well drug responses in PDxOs mirrored responses in PDXs, we selected drugs that showed very distinctive responses or resistance patterns in PDxO lines. For example, 6 of the 12 TNBC PDxO lines responded with remarkable sensitivity to birinapant, a SMAC mimetic^26^, while the remaining 6 TNBC lines were resistant to high doses of birinapant (Fig. 7b). We tested birinapant in vivo on seven PDX lines that were predicted to span a range of birinapant sensitivity according to PDxO results. TNBC PDXs predicted to be resistant to birinapant (HCI-001, HCI-002 and HCI-019) resulted in progressive disease similar to controls, whereas PDX lines predicted to be sensitive to birinapant (HCI-015, HCI-023 and HCI-027) resulted in tumor shrinkage. HCI-012 had an intermediate response, showing initial shrinkage followed by growth (Fig. 7c).

As another example of concordant drug responses, we noted that HCI-015 and HCI-027 PDxOs showed exceptional responses to two γ-secretase inhibitors, RO4929097 and LY3039478 (Fig. 6a and Extended Data Fig. 9c). We were unable to obtain LY3039478 for in vivo studies but found that effects of RO492097 in PDXs were concordant with PDxO results for the two responsive and two unresponsive lines (Fig. 7d). Interestingly, we observed a *NOTCH1* CN amplification (Fig. 1) that may explain the favorable response of HCI-027 to RO4929097.

We next investigated whether drug combinations could be efficiently tested in PDxO culture using synergy matrices. A potential synergistic interaction between birinapant and SN-38 (the active metabolite of the prodrug irinotecan) was reported in ovarian cancer cells in vitro^27^. Using both drugs separately and in combination, we determined that this synergy was also apparent in breast cancer PDxOs, especially for birinapant-sensitive tumors like HCI-023 (Extended Data Fig. 9d). Indeed, treatment of PDX HCI-002 (birinapant resistant) showed little or no benefit with the combination versus irinotecan alone, while the combination treatment improved response in the partially birinapant-sensitive line HCI-012 (Fig. 7e). In the birinapant-sensitive line HCI-023, either birinapant or irinotecan was able to completely eliminate tumors, but the combination treatment resulted in a more durable response following treatment cessation than either single agent (Fig. 7e,f). These results suggest that PDxOs can predict drug responses in PDXs accurately and may be used to identify new treatment options for breast cancer.

### PDxO drug screening can inform clinical care

**Fig. 8:**
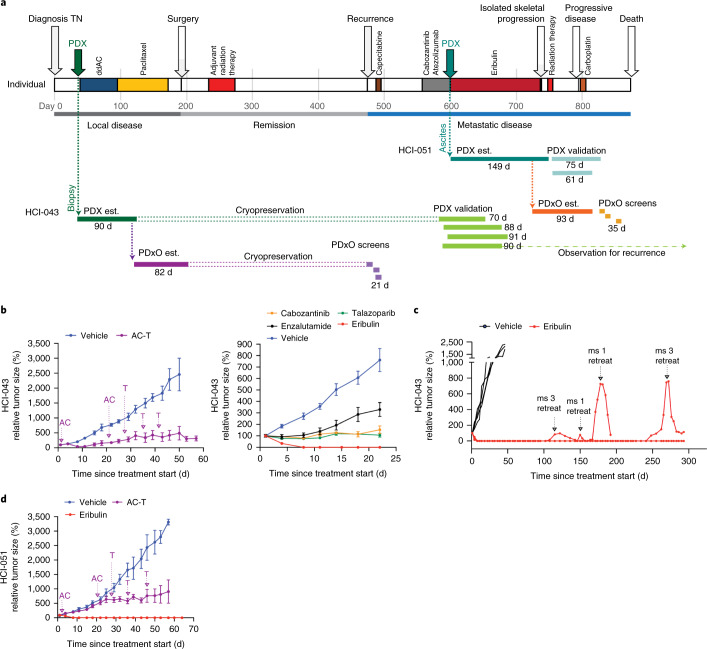
PDxO screening can be performed in real time with clinical care. **a**, Timeline of the individual HCI-043, including clinical history, patient-derived model establishment (PDX and PDxO est.), PDxO drug screens and in vivo validation of responses in PDX. Model development and drug screening/testing were done with both the pretreatment biopsy sample (HCI-043) and the metastatic ascites sample (HCI-051), with similar results. ddAC, dose-dense doxorubicin and cyclophosphamide. **b**, Treatment of the HCI-043 PDX with human-matched neoadjuvant therapy (doxorubicin + cyclophosphamide followed by paclitaxel (AC-T); left) or with drugs selected from the PDxO screen (right). Arrows indicate the sequencing of AC-T drug treatment. Data are shown as mean ± s.e.m.; left: AC-T treatment group, *n* = 5 mice; vehicle group, *n* = 4 mice; right: cabozantinib, *n* = 4 mice; talazoparib, *n* =4 mice; enzalutamide, *n* = 4 mice; eribulin, *n* = 5 mice; vehicle group, *n* = 8 mice. **c**, Follow-up of HCI-043 PDX mice after stopping treatment with eribulin following three doses. Two different mice (ms) exhibited recurrence off-treatment, but the tumors regressed after treatment was restarted. No resistant tumors were detected over the lifespan of the mice (293 d after initial treatment began). **d**, Treatment of HCI-051 PDX with AC-T, as in **b**. Data are shown as mean ± s.e.m.; treatment and vehicle groups, *n* = 5 mice for all PDX lines. Source data

To illustrate that PDxO drug screening can be feasibly performed to inform clinical care, we present the case of a 43-year-old individual with stage IIA TNBC (Fig. 8a). A treatment-naive biopsy was taken to make a PDX, then they received preoperative chemotherapy with doxorubicin and cyclophosphamide, followed by paclitaxel (AC-T therapy, per standard of care). Surgical pathology showed complete pathologic remission, and adjuvant radiation therapy ensued per standard of care. However, rapid growth of the PDX (HCI-043) in the interim suggested a high risk of recurrence^13^.

**Extended Data Fig. 10:**
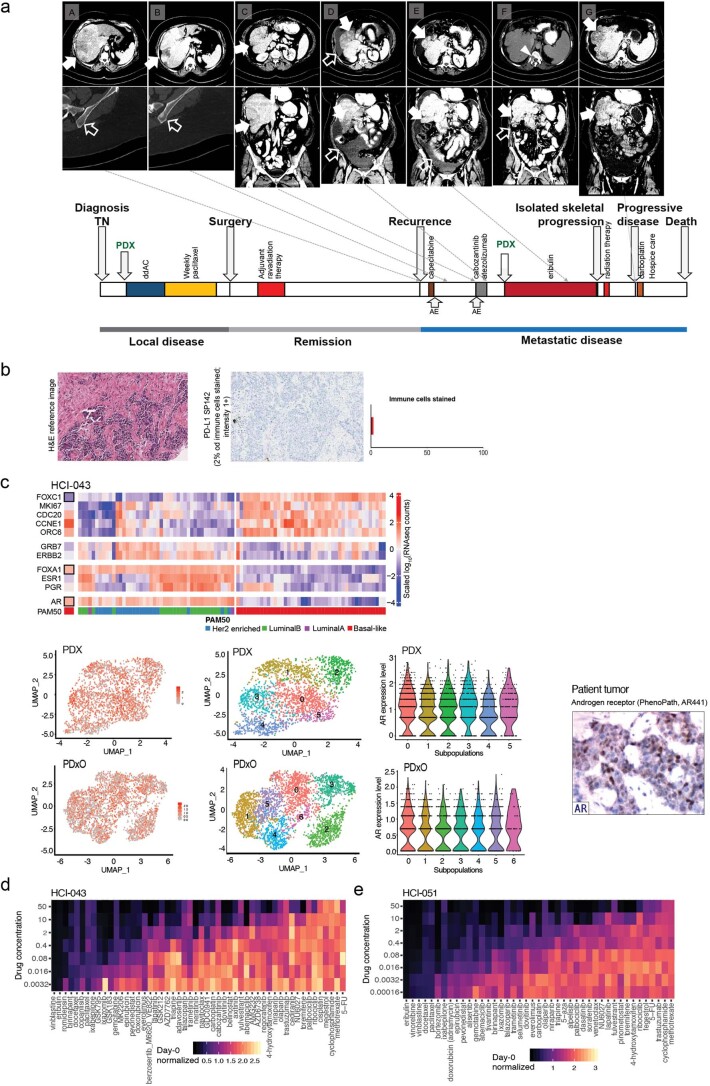
Application of PDxO screening to precision oncology. **(a**) Representative imaging data (upper panel) and a timeline representing the natural history of individual HCI-043’s breast cancer (lower panel) Events are as follows: A. Diagnosis of early recurrent disease metastatic to the liver (solid arrow). No skeletal metastases (empty arrow). B. No response to capecitabine; new onset skeletal metastases (empty arrow). C. Initiation of cabozantinib and atezolizumab; liver metastases still present (solid arrows). D. No response to cabozantinib and atezolizumab; progression of the hepatic metastases (solid arrow) and production of malignant ascites (empty arrows). E. After 3 cycles of the PDxO-informed eribulin treatment, the patient achieved a complete radiographic remission of the hepatic metastases (solid arrows). The malignant ascites also regressed somewhat (empty arrow). F. After 5 cycles on eribulin, there was complete remission of the malignant ascites (empty arrow) and continued complete remission of the hepatic metastases (solid arrow). However, new onset isolated metastasis in T12 vertebrae (arrowhead) required discontinuation of eribulin and treatment with radiation therapy. G. Recurrence of the hepatic metastases 2 months after withholding the eribulin (solid arrows). **(b)** H&E staining and PD-L1 staining of HCI-043 patient’s tumor. The tumor tested low but positive for PD-L1 on the basis of an FDA-approved commercially available test. Due to the nature of the test a scalebar is not available. **(c)** (upper panel) RNA-seq data showing expression of genes associated with the luminal androgen receptor (LAR) subtype in HCI-043 patient tumor. (lower panel) scRNA-seq data showing androgen receptor (AR) expression (red; left side) in all tumor cell clusters (middle) in HCI-043 PDX and PDxOs. Immunohistochemistry for the androgen receptor on the patient tumor was detected by a commercial vendor (PhenoPath; right side). **(d-e)** Dose response heatmaps showing results of drug screening on the pre-treatment breast tumor biopsy model HCI-043 **(d)** and the post-treatment metastatic ascites sample **(e)** from timepoint D on the timeline in panel a, HCI-051. Coloration of these heatmaps indicates CellTiter-Glo 3D cell viability assays that were normalized to day 0 ranging from black (cytotoxic) to yellow (growth), which have been scaled respectively. The drug order on both plots is sorted by GRaoc.

Despite the complete pathologic remission, which portends a favorable prognosis^28,29^, the individual experienced early metastatic recurrence in the liver (Fig. 8a and Extended Data Fig. 10a). Their first line of therapy in the metastatic setting (to which we were blinded) consisted of capecitabine, but they experienced progression with new onset skeletal metastases (Fig. 8a). The tumor was weakly positive for PD-L1 (Extended Data Fig. 10b), so the next line of therapy consisted of cabozantinib and atezolizumab in the context of a clinical trial. Cycle two of this regimen was delayed due to adverse events, but eventually the investigational therapy was stopped due to progressive disease (Fig. 8a).

In the meantime, we had developed and cryopreserved the HCI-043 PDxO line corresponding to the untreated primary tumor. After learning of her recurrence, we thawed the organoids and screened a library of FDA-approved and experimental drugs and performed whole-genome sequencing and bulk and single-cell RNA-seq on the PDX model. Commercial genomics analysis did not reveal any clinically actionable mutations, but RNA-seq analysis showed that the tumor was of the luminal androgen receptor subtype^30^, with *AR* mRNA expressed in all tumor clones and AR protein detected in a subset of cells by clinical IHC (Extended Data Fig. 10c). In the organoid drug screen, two FDA-approved breast cancer drugs, eribulin and talazoparib, emerged as promising candidates, while several of the chosen clinical therapies, including 5-fluorouracil (the active metabolite of capecitabine) and cabozantinib, did not appear to be effective (Extended Data Fig. 10d). We also noted genomic deletions in *BRCA1*, *BRCA2* and *RAD50* (Fig. 1), which may explain the enhanced sensitivity to talazoparib.

In vivo testing on the HCI-043 PDX verified a non-complete response to the individual-matched AC-T therapy (with the caveat that doses lower than the human-equivalent dose are required in mice) and confirmed PDxO data that eribulin was the best drug tested (Fig. 8b). Enzalutamide (tested due to the luminal androgen receptor phenotype), cabozantinib and talazoparib each slowed growth of the tumor compared to vehicle-treated controls but did not cause tumor regression. The durability of the response to eribulin was tested by stopping treatment after three doses and following the animals for tumor recurrence. In all mice, there was either no recurrence or complete regression on retreatment with a single dose of eribulin. We followed mice for 293 d, when we had to euthanize them for old age (Fig. 8c).

With Institutional Review Board (IRB) approval, we returned our results to the clinic. After the individual was put on eribulin, the hepatic metastases sustained a complete remission for a period of nearly 5 months (Fig. 8a and Extended Data Fig. 10a). In the meantime, we also generated PDXs and PDxOs (HCI-051) from the AC-T-resistant tumor from an ascites sample collected from the individual just before eribulin treatment (Fig. 8a). The metastatic models were more resistant to AC-T but were still completely responsive to eribulin (Fig. 8d and Extended Data Fig. 10e).

Although the individual’s liver metastases and ascites regressed completely on eribulin (Extended Data Fig. 10a), there eventually was isolated progression in bone. Eribulin was withheld and radiation therapy ensued. While off systemic therapy, the liver metastases remained in remission for two more months but eventually returned. It is unknown whether the recurrent liver metastases were sensitive to eribulin; a different therapy was given at that point (carboplatin; Fig. 8a). Carboplatin was not effective, and, unfortunately, the individual died. In an analysis of all therapies given, the PFS and time to next systemic therapy (TTNT) that was achieved with the PDxO-informed therapy (eribulin) was 3.5 and 4.8 times longer than the PFS and TTNT achieved with the prior therapy (138 d versus 41 d and 197 d versus 41 d, respectively). Of note, a recent clinical trial using genomics-informed therapies in individuals with metastatic cancer (MOSCATO-01) used a matched therapy:prior therapy PFS ratio of 1.3 as a benchmark of success^31^. These results suggest that functional drug testing with PDxOs is feasible and can give beneficial results in real time with clinical care.

## Discussion

We report development and characterization of a collection of PDX and matched organoid cultures. A bank of human breast tumor organoids has been previously described by Sachs et al.^20^, so it is important to note how our collection is different. The organoid collection described by Sachs et al. was mainly developed from primary, untreated breast tumors. While primary breast tumors are easy to obtain during surgery, these tumors are curable 70–80% of the time with standard therapy; 65% of our collection comes from treatment-resistant tumors and represents recurrences from eight different metastatic sites. Some are models of primary–metastatic pairs or longitudinal collections over time from the same individuals.

Our collection facilitates drug screening on models of advanced breast cancer representing the human population likely to be in clinical trials. Although short-term 2D culture of PDX-derived tumor cells was previously reported as a drug screening tool^32^, there are advantages to stable cultures. One caveat of the short-term 2D approach is that the cultures are transient and contaminated with non-tumor cells, and one cannot use the same cultures for future studies and comparisons as new drugs and techniques arise. In our experience, human tumors can be quite unstable immediately after being placed in culture (2D or 3D); this may affect drug responses. Despite the longer time taken, we find that stable PDxO-based drug screening is feasible and cost-effective and allows in vivo validation of results in the matching PDX models. In a case study of a individual with TNBC with early metastatic recurrence, we found that drug responses in the models, to which the treating oncologist was initially blinded, aligned with observed clinical responses. Our study was not originally designed to prospectively inform human care, but compelling results uncovered a potentially effective FDA-approved drug and resulted in IRB approval to return those results to the clinic. Treatment with the PDxO-directed therapy resulted in a complete response for the individual and PFS and TTNT periods that were 3.5 and 4.8 times longer, respectively, than previous therapies. While only an example, these data suggest that more work should be done to determine whether PDxO screening can be leveraged to identify vulnerabilities to FDA-approved or investigational agents and inform medical decisions. Future trials will be required to determine if functional precision oncology using human-derived models can improve outcomes in individuals with metastatic breast cancer. Because only ~30% of breast cancers can be engrafted as PDXs, using PDxOs for precision medicine is not possible in every case. However, we and others have reported that the engraftable tumors are those that eventually progress to the metastatic stage and are more likely to require tailored therapy^13,19,33^.

Our data also showed that PDxO screening can uncover experimental drugs with therapeutic potential. We found that birinapant showed potent activity in certain TNBC organoids, and this was validated in PDXs. Others have shown that birinapant has selective activity for TNBC compared to ER^+^ breast cancers^34,35^, and a similar SMAC mimetic (LCL161) combined with paclitaxel showed clinical activity only in TNBCs expressing a TNFα signature^36^. These data demonstrate that unbiased screening in PDxOs can identify experimental agents with clinical activity in particular cancer subtypes. Different types of assays could also be used based on cancer stem cell activity^37^, apoptosis^38^ or cell phenotype^39^.

PDxO drug response assays are not without limitations. Although we were able to discern cytotoxic effects in our assays, we were unable to reliably detect activity of drugs that convey less potent activity. For example, response of several ER^+^ PDxoX lines to fulvestrant is shown in Fig. 3; however, response to fulvestrant was difficult to discern in PDxO models in short-term organoid assays (Fig. 6). Similarly, 4-hydroxytamoxifen responses in PDxOs did not correlate with ER status (Fig. 6). It is likely that 4-day drug exposure on slow-growing models, such as ER^+^ breast cancers, is not enough to reveal vulnerabilities, especially when the main biological outcome is cytostasis or stable disease (as for fulvestrant). We also noted lack of cytotoxic effects with CDK4/CDK6 inhibitors (CDK4/CDK6i) in short-term organoid assays. This was not surprising because PDX models take 10–30 days to show a cytostatic effect with CDK4/CDK6i^40,41^. However, cell lines grow much more quickly and can show rapid effects with CDK4/CDK6i^42^. Thus, 4-day PDxO drug responses are best for identifying drugs with cytotoxic activity. Future work will determine whether longer-term drug exposure, possibly with passaging, will be a better read-out for less potent, yet clinically relevant, drug activity.

It is important to consider intertumor and intratumor heterogeneity when models derive from a particular tumor site(s). In the case of HCI-043/HCI-051, models were made from the untreated breast tumor and from ascites following metastatic recurrence. Both models showed strong response to eribulin, and, while the individual’s liver metastases regressed completely while on eribulin, a metastasis in the bone appeared during this treatment. It is unknown whether the eribulin-resistant bone metastasis in the individual was a different tumor clone that was perhaps not represented in our models. Attempts to make models from as many anatomic locations as possible should help inform these questions. Models derived from circulating tumor cells (CTCs) may better represent the heterogeneity of the disease in the individual^43^, but it is difficult to derive models from CTCs for certain cancers, including breast cancer, due to low CTC numbers. Integration of functional data from human-derived models with genomic data from circulating tumor DNA may allow prioritization of drugs that might be effective in multiple tumors.

One limitation of common models of human cancer is lack of human stroma, including immune cells, and growth in an imperfect environment. Human stromal cells are replaced by mouse stroma during PDX development^13,44^. Human immune cells can be engrafted to seed ‘humanized’ immune systems in mice with PDXs^45,46^, but this approach is plagued by variability of immune engraftment, and the full functionality of tumor–immune interactions has not been well established. In our PDxO system, mouse cells are removed during organoid propagation; some tumors recruit an aggressive stroma that compromises the ability of the organoids to thrive. As a result, the organoid platform, like most other in vitro systems, does not fully recapitulate the tumor microenvironment, which limits the classes of drugs that can be tested in this system. Because the macroenvironment of tumors is also critical for their behavior, it would be interesting to compare how PDXs from metastatic breast cancers, and their derived organoids, might differ when engrafted into mouse mammary fat pads versus into the brain or other sites that match the origin of the human metastasis.

In summary, this work provides a large, clinically relevant resource of paired in vivo and in vitro human-derived models of breast cancer, with an emphasis on the most difficult cases for which research advances are urgently needed. We show that these models can be used for drug screening and discovery, and our methods are also conducive to conducting functional precision medicine in real time with clinical care.

## Methods

Specific resources used in this study include details for antibodies (Supplementary Table 3), chemicals and other reagents (Supplementary Table 4), oligonucleotides (Supplementary Table 5), drug screening details (Supplementary Table 6) and in vivo drug doses and regimens (Supplementary Table 7). Further information on research design is available in the Nature Research Reporting Summary linked to this article.

### Development of breast cancer PDX lines

The University of Utah IRB (protocols 89989, 91596 and 10924) approved human sample collection following informed consent. IRB 91596 allows us to return drug testing data to the clinic, which was performed for one individual with written informed consent. The deidentified clinical information in this study is published in accordance to the ethics approvals for this study. The University of Utah Institutional Animal Care and Use Committee (IACUC) approved all procedures using live animals. Detailed tissue processing and implantation protocols have been previously described^47^. Briefly, female immune-compromised mice (NOD *scid* gamma (NSG), Jackson Laboratory stock 5557; NOD/*scid*, Jackson Laboratory stock 1303 or NOD *rag* gamma (NRG), Jackson Laboratory stock 7799) were used to generate PDXs, typically at the age of 3–4 weeks. In rare cases, with younger mice not available or for ovariectomy experiments, we used mice up to 10 weeks old. Fresh or thawed human breast tumor fragments were implanted into the cleared inguinal mammary fat pad. In the case of bone metastasis samples, bone fragments were coimplanted. For liquid specimens, pleural effusion or ascites fluid, samples were processed as previously described^47^, and 1–2 million cells were injected into cleared mammary fat pads in 10–20 μl of commercial Matrigel (Corning). For ER^+^ tumors, mice were given supplemental E2 (see below). Mice were monitored for health, and tumors were measured weekly with digital calipers once growth was observed. When tumors reached 1–2 cm in diameter, tumors were aseptically collected and reimplanted into new mice or viably banked^47^. The maximum tumor diameter approved by the IACUC was not exceeded. The initial tumor was termed ‘passage 0’ (P0), and passages continued to be tracked with each generation. Clinical information and individual demographics for PDX lines combined can be found in the Baylor College of Medicine (BCM) PDX Portal (https://pdxportal.research.bcm.edu/) and on the Welm lab research website (https://uofuhealth.utah.edu/huntsman/labs/welm-labs/research.php).

### Pathogen testing and removal of LDEV or *C. bovis*

PDX tumors were tested for select human pathogens (Epstein–Barr virus, human cytomegalovirus, hepatitis B, hepatitis C, human immunodeficiency virus 1, human immunodeficiency virus 2 and lymphocytic choriomeningitis virus) and confirmed negative for *C. bovis* and LDEV using commercial testing from IDEXX Laboratories. Other mouse pathogens were monitored using sentinel testing in the vivarium. The facility is specific pathogen free, and no positive results have been obtained during the development of these PDXs.

#### LDEV removal

LDEV may transmit through serial transplantation of infected tissue in mice or by use of LDEV-contaminated Matrigel-like products^48^. Some early PDX tumors (HCI-010, HCI-013 and HCI-013EI) were generated with Engelbreth–Holm–Swarm (EHS)-derived matrix^47^ before realizing that EHS tumors, and some commercial Matrigel lots, contained LDEV. To decontaminate LDEV^+^ tumors, including EHS tumors, we utilized various methods. In two of the three infected lines, it was sufficient to perform FACS using positive selection with anti-human CD298 as a universal cell surface marker of human cells^49^ and negative selection for mouse-specific CD45, followed by retransplantation into mice. For HCI-010, this method was insufficient even after adding additional negative selection with antibodies specific for mouse CD11b and F4/80, so the line was passaged once through 3- to 4-week-old immune-deficient female rats (NIH-RNU; Charles River stock 568) to achieve LDEV^–^ status. For FACS, freshly collected PDX or EHS tumors were processed to single cells^47^, stained with PE-conjugated anti-human CD298 and FITC-conjugated anti-mouse CD45 and sorted with a BD FACS Aria (for gating strategy, see Supplementary Fig. 45a). Human CD298^+^ and mouse CD45^–^ tumor cells were collected and washed in HBSS (HyClone), followed by resuspension in LDEV-free Matrigel (Corning or matrix prepared in the lab; see below). Cells (0.5 to 2 million) in 10–20 μl of Matrigel were injected into cleared mammary fat pads of 3- to 4-week-old NSG mice; tumors were collected when they reached 2 cm in diameter. Tumors were tested for LDEV using IDEXX testing. LDEV-free matrix was prepared by growing EHS tumors subcutaneously in 8- to 10-week-old male or female C57BL/6J mice (Jackson Laboratory stock 664), collecting and preparing single-cell suspensions^47^ and FACS to remove macrophages, as described above. After LDEV^–^ status was confirmed, tumor stocks and matrix were prepared as previously described^47^.

#### *C. bovis* removal

Recently, institutions reported *C. bovis* infection of PDXs with rapid transmission in immune-deficient mice. Symptoms of infection include red ears, rash and alopecia on the face and neck. We screened for *C. bovis* using fur swabs and IDEXX testing. Positive tests resulted in immediate killing and disinfection with 2% chlorhexidine gluconate solution by submerging the entire mouse for 5 min^50^. Tumors were aseptically collected without allowing skin or fur to touch the tumor. *C. bovis* negativity was confirmed on the next passage.

### Estrogen delivery

We modified our published protocol^47^ by reducing the dose to 0.4 mg of E2 in beeswax pellets. E2 was also delivered in drinking water using a protocol kindly shared online by the Wicha lab (http://www.med.umich.edu/wicha-lab). Briefly, a 2.7 mg ml^–1^ stock of β-estradiol in 100% ethanol was diluted to a concentration of 8 μg ml^–1^ in sterile drinking water. We changed water once per week, because there was no significant difference in plasma E2 concentrations with once versus twice per week changes (not shown). E2 is light sensitive, so we tested the stability of the E2 in water over time in clear or amber bottles. We observed no significant differences in mouse plasma levels (not shown); we use clear bottles. For all standard experiments (except those grown as estrogen-independent sublines), ER^+^ tumors were grown in mice with a 0.4-mg E2 pellet implanted subcutaneously at the time of tumor implantation, followed by administration of E2 in the drinking water beginning 4 weeks after tumor implantation until the tumor was collected.

### Development of estrogen-independent ER^+^ breast PDX models

ER^+^ PDX tumors were collected and transplanted into ovariectomized mice without E2 supplementation. Tumor sublines that grew under these conditions were considered to be estrogen independent and were given the designation -EI. To generate -EI lines, 6- to 8-week-old mice were ovariectomized bilaterally using two separate dorsal incisions parallel to the midline under general anesthesia using standard procedures (https://www.criver.com/sites/default/files/resource-files/ovariectomy.pdf) immediately followed by tumor implantation into the mammary fat pad. To minimize pain and distress of the ovariectomy, mice were given buprenorphine (0.1–0.2 mg kg^–1^) before and after surgery and carprofen (5 mg kg^–1^) daily for 3 d following surgery. In the case of HCI-013EI, a 2-week culture step in phenol red-free HBEC medium^47^ supplemented with charcoal-stripped FBS occurred between the steps of tumor growth in ovariectomized mice and retransplantation. Future passages of -EI lines occurred in intact mice with no E2 supplementation.

### Establishment of PDxO cultures

For PDxO preparation, PDX tissue chunks were digested in a GentleMACS dissociator in warm advanced DMEM/F12 with GentleMACS human tumor dissociation enzymes and 10 μM Y-27632 added. After digestion, differential centrifugation was performed to enrich for organoids and deplete single cells. Organoids were embedded in 200-μl Matrigel domes, which were plated in six-well tissue culture plates onto a 50-μl Matrigel base layer. After a 5-min incubation period, plates were flipped, and Matrigel domes were solidified for 10 min before subtype-specific culture medium was added. For all breast cancer subtypes, 10 μM Y-27632 was added fresh to the PDxO base medium (Advanced DMEM/F12 with 5% FBS, 10 mM HEPES, 1× Glutamax, 1 μg ml^–1^ hydrocortisone, 50 μg ml^–1^ gentamicin and 10 ng ml^–1^ hEGF). Additionally, for HER2^+^ PDxOs, 10 nM heregulin-β1 was added, and for ER^+^ PDxOs, 100 ng ml^–1^ FGF2 and 1 mM NAC was added. Medium was exchanged every 3 to 4 d, and, once mature, cultures were passaged by incubating in dispase solution (20% FBS in dispase with Y-27632), followed by a wash step with base medium and a dissociation step in TrypLE Express. Single cells were seeded at 200,000–400,000 cells per dome. To eliminate mouse cells, organoid cultures were either differentially centrifuged several times after dispase incubation or sorted by FACS. For FACS, single-cell suspensions of dissociated organoid cultures (obtained with the regular passaging process) were incubated with human and mouse anti-FcR, followed by antibody staining (AlexaFluor647 anti-mouse CD90.2, AlexaFluor647 anti-mouse CD29, AlexaFluor488 anti-human CD326 and FITC anti-human CD298) (for gating strategy, see Supplementary Fig. 45b). After sorting, human cells were cultured on ultra-low attachment plates overnight to allow aggregation into organoids and embedded in Matrigel domes the next day. Minimal to zero mouse content was confirmed by RT–qPCR using mouse *GAPDH* primers. For cryopreservation, mature PDxO domes were frozen in PDxO base medium with 20% FBS, 10% DMSO and 10 μM Y-27632.

### PDxO nucleic acid extraction and RT–qPCR

Matrigel domes from mature PDxO cultures were mechanically disrupted, washed twice in cold, unsupplemented Advanced DMEM/F12 medium and centrifuged. The pellet was lysed in RLT Plus with β-mercaptoethanol and stored at −80 °C. Samples were incubated at 37 °C for 5 min and vortexed for 1 min. Lysate was transferred to QiaShredder columns and centrifuged. DNA and RNA were isolated from the flow-through using a Qiagen AllPrep kit following the manufacturer’s instructions. Nucleic acid concentrations were determined by Qubit BR assays. DNase-treated RNA was reverse transcribed using a SuperScript IV VILO Master Mix with ezDNase Enzyme kit. One nanogram of cDNA per sample was run in 5-μl reactions in technical quadruplicate using PowerUp SYBR Green Master Mix against 500 nM of each forward and reverse primer (Supplementary Table 5). Reactions were run on a Roche LightCycler 480 using the PowerUp SYBR Green-recommended standard cycling mode (primer melting temperature (*T*_m_) ≥ 60°C). Technical quadruplicate *C*_t_ values were averaged, and a threshold of 32 cycles was set. Average values above threshold are reported as not detectable. *C*_t_ values were normalized to human *GAPDH*. Cell lines were obtained from ATCC (MCF7, HTB-22; MDA-MB-231, HTB-26; MDA-MB-468, HTB-132; T47D, HTB-133) or Lonza (hMSC, PT2501) and cultured as recommended. MDA-MB-361 was obtained from another lab and validated by IDEXX CellCheck 9-Human STR marker profiling. Human PBMCs were donated anonymously through the HCI Biorepository.

### IHC staining of PDX tumors and PDxOs

PDX tissues were fixed and processed as described previously^47^. For fluid samples, for example, pleural effusion or ascites fluid, cells were centrifuged and fixed in 2% paraformaldehyde before embedding in histogel for sectioning. To prepare formalin-fixed paraffin-embedded PDxO blocks, PDxO domes were mechanically disrupted in cold PDxO base medium, washed and resuspended in Matrigel. The Matrigel/organoid mixture was pipetted into a chamber slide and incubated at 37 °C to solidify. To prepare histology blocks, a published protocol^51^ was followed, with the exception that 65 °C warm histogel was used for pipetting bottom and top layers. H&E staining was performed to confirm PDxO content, and IHC staining was performed for ER, PR, HER2, pan-cytokeratin, human vimentin, E-cadherin, human mitochondria, mouse and human CD45, EpCAM and human-specific cytokeratin (CK-CAM5.2). To quantify Ki67^+^ nuclei, three independent Ki67 staining sets were performed, and the percentage of Ki67^+^ nuclei relative to hematoxylin^+^ nuclei was quantified using ImageJ. Antibody details are provided in Supplementary Table 3. For each staining set, one random image per slide was quantified. All IHC images were acquired on an Olympus BX50 microscope with a UplanFI ×20/0.50-NA objective using a Canon EOS camera and acquisition software EOS Utility 2 version 2. If brightness and/or saturation were adjusted, it was applied to the entire image using Adobe Photoshop CC 2019.

### Cytospin and IF staining of PDxOs

Mature PDxO domes were mechanically disrupted and incubated in dispase solution for 30 min at 37 °C. PDxOs were washed twice with cold PDxO medium and fixed in fixing solution (2% paraformaldehyde and 0.01% Tween 20 in PBS) for 20 min at room temperature. After centrifugation, PDxOs were permeabilized in PBS with 0.5% Triton X-100 for 30 min and centrifuged, and two aldehyde blocking steps were performed by incubating in aldehyde block solution (1 mg ml^–1^ NaBN_4_ and 0.01% Tween 20 in PBS) for 5 min. After resuspending in 0.01% Tween 20 in PBS, PDxOs were centrifuged and washed once in 0.01% Tween 20 in PBS. The pellet was resuspended in 1 ml of PBS with 2% bovine serum albumin (BSA). PDxO solution (100 μl) was loaded on cytospin slides and spun at 2,000 r.p.m. for 2 min at room temperature. Slides were dried overnight, and ER/EpCAM double IF staining was performed the next day. Cytospin slides were rehydrated in PBS containing 0.5% Triton X-100 for 20 min at room temperature, washed in PBS and incubated in blocking buffer (50 mM NH_4_Cl, 10% goat serum, 5% BSA, 0.5% Tween 20 and 0.2% Triton X-100 in PBS) with mouse FcR blocking reagent. Slides were incubated with mouse anti-human ER antibody, ER antibody was removed and anti-human EpCAM antibody was diluted in blocking buffer (10% goat serum, 5% BSA, 0.5% Tween 20 and 0.2% Triton X-100 in PBS) and incubated with M.O.M. diluent. Slides were washed (PBS with 0.05% Tween 20) and incubated with secondary antibodies (anti-mouse AF555 and anti-rabbit AF488), which were diluted in blocking buffer with M.O.M. diluent. After washing, PDxOs were stained with DAPI, washed and incubated in 0.1% Sudan Black solution for 7 min to quench autofluorescence and washed and covered with mounting medium and cover slips. Images were acquired using an Olympus IX81 microscope (camera/sensor: Hamamatsu C11440/Orca Flash 4.0LT; lens: ×40/0.6-NA/∞; light source: U-LH100HGAP0; filters: DAPI (Brightline 506B), green fluorescent protein (GFP; Semrock 30LP B OMF), red fluorescent protein (RFP; Olympus DSU-MRFP HQ) and Olympus CELLSENS software). Exposure adjustment and deconvolution was performed on raw files using a constrained-iterative algorithm. ImageJ was used to generate overlays and resolution changes. The final resolution was 300 dpi on TIFF files. Brightness and saturation were adjusted on the entire image using Adobe Photoshop CC 2019.

### Quantification of live-cell area and relative PDxO growth

Organoids were seeded as single cells in 9-μl Matrigel domes with up to 11,300 cells per well onto 3-μl Matrigel base layers in a 48-well glass-bottom plate (MatTek, P48G-1.5-6-F). For live-cell area quantifications, 0.5 μM calcein AM and 0.5 μg ml^–1^ Hoechst dye was added to each well and incubated for 1 h at 37 °C. Wells were washed once with HBSS and imaged using an inverted confocal microscope at the HCI Cell Imaging Core. The area of calcein AM^+^ organoids was quantified using ImageJ. To quantify relative PDxO growth, medium was replaced with 250 μl of HBSS, and 100 μl of CTG-3D was added per well. Matrigel domes were mechanically disrupted and placed on a shaker in the dark for 20 min (500 r.p.m. at room temperature). Plates sat at room temperature for 10 min in the dark before samples were read on an EnVision XCite plate reader (PerkinElmer, 2105-0020).

### Three-dimensional drug screening of PDxOs

Mature organoids were collected from culture using dispase treatment at 37 °C for 25 min. Five thousand to ten thousand cells (~50 organoids) were seeded per well in 384-well tissue culture plates, each comprising a solidified 10-μl Matrigel base layer and 30 μl of subtype-specific PDxO medium supplemented with 5% (by volume) Matrigel. In a separate 384-well plate, 16 wells were similarly seeded to generate day 0 seeding controls. Plates were incubated at 37 °C and 5% CO_2_ overnight. For the seeding control plate, 30 μl of culture medium was added and assayed with CTG-3D to generate a seeding control value at the time of dosing. A separate drug plate was prepared with an eight-point serial dilution, and 30 μl of each condition, in technical quadruplicate, was transferred to seeded 384-well plates. Dosed PDxO plates were covered with Breathe-Easy seals, incubated for 96 h (37 °C and 5% CO_2_) and assayed with CTG-3D. Raw luminescent values from each condition were normalized to the day 0 seeding control value.

### Scoring dose–response curves for PDxO

Response scores were calculated based on a published growth rate normalization strategy^25^. When screening results did not fit the sigmoidal curve, GRmetrics produced missing values for GR_50_ statistics. This occurred in organoids that did not respond to a compound and when little or no cytotoxicity was observed. We compared results for GR_50_ versus GI_50_ (the concentration at which there was 50% growth inhibition) to address whether GR_50_ or GI_50_ metrics inflate drug potencies in our models based on faster or slower growers (Extended Data Fig. 6b). The GI_50_ equation that we used to address this was

$$\frac{{\left( {{{T}} - {{T}}_0} \right)}}{{\left( {{{C}} - {{T}}_0} \right)}} \times 100 = 50$$

where *T* is the response measure at endpoint, *T*_0_ is the response measure at baseline and *C* is the response measure of the control sample at endpoint, that is, DMSO. The same variables were used for the GR_50_ equation, which is

$$2^{\frac{{{{{\mathrm{log}}}}2\left( {{{T}}/{{T}}_0} \right)}}{{{{{\textrm{log}}}}2\left( {{{C}}/{{T}}_0} \right)}}} - 1$$

Comparing the GR_50_ and GI_50_ for each data point showed a Pearson correlation coefficient of 0.976 (Extended Data Fig. 6b). Differences between the two metrics are shown in Extended Data Fig. 6b, showing the residuals of all drug–sample pairs to the regression line. When we grouped the data into faster and slower growers, we did not find that the GR-adjusted metrics inflates drug potencies in slower-growing lines (Extended Data Fig. 6b).

We used AOC values throughout this manuscript. This recommendation is also suggested as ‘best practice’ to account for differences in cell growth rates when using different culture platforms^52^. We chose AOC to interpret drug responses based on the observation that often a flat or linear curve fit to the dose–response curves did not always cross the 50% viability threshold (a necessary feature to calculate GR_50_ or GI_50_ scores). AOC statistics, however, could still be generated. Specifically, we observed 332 missing data points for GR_50_, 217 missing data points for half-maximum inhibitory concentration (IC_50_), 274 missing data points for GI_50_ and 0 missing data points for GR_aoc_. Combenefit software was used to generate Loewe synergy plots of PDxO drug screening results^53^. Synergy response data were generated across *n* = 3 biological replicates, each comprised of *n* = 4 technical replicates.

### Genomic characterization of human samples and PDX and PDxO models

#### Whole-exome sequencing (WES)

Genomic sequencing was performed at the Huntsman Cancer Institute High-Throughput Genomics and Bioinformatics Core, with more details at https://uofuhealth.utah.edu/huntsman/shared-resources/gba/htg/. Agilent SureSelectXT Human All Exon V6+ COSMIC or Agilent Human All Exon 50-Mb library or IDT xGEN Human Exome v2 with Nextera Flex library preparation protocols were used with inputs of 100–3,000 ng of sheared genomic DNA (Covaris).

#### Sequence alignment and variant calling

Fastq files were uploaded to the PDXNet shared data pool for alignment and variant calling on the SevenBridges cloud interface (https://www.sevenbridges.com/). PDXNet-approved sequencing analysis methods^54^ were utilized to generate variant call format files (VCFs) absent of potential mouse read contaminants (see Code availability). VCF files were converted into mutation annotation format using vcf2maf v1.6.17, which implements VEP release 95.3 (ref. ^55^).

#### Filtering WES data for cancer mutations

Mutation calling was restricted to previously identified cancer genes and mutations. Missense mutations were displayed if they met stringent criteria. If a missense mutation was found in an established germline predisposition gene and associated with breast cancer samples^56^, we also required deleterious or likely deleterious labels by SIFT v5.2.2 (ref. ^57^), a damaging or likely damaging label by PolyPhen v2.2.2 (ref. ^58^), a pathogenic or likely pathogenic alteration by CLIN_SIG version 201810 (ref. ^59^) or a ‘HIGH’ impact rating by Ensembl release 95 ensembl-variation version 95.858de3e (ref. ^55^). In addition to the genes reported by Huang et al., we included *ESR1* and additional genes involved in DNA damage repair, including *FANCD2*, *FANCE*, *FANCF*, *FANCG*, *HMBS*, *POLD1*, *FANCI* and *FANCL*. Missense mutations in germline predisposition genes required a gnomAD population frequency less than 0.001 or be absent from gnomAD v170228 (ref. ^60^). We also present nonsense, in-frame, frameshift and predicted splice site mutations from previously predicted somatic cancer genes^14^ and germline predisposition genes^56^, only if their gnomAD frequency was less than 0.001 or not reported. Mutations presented in figures were manually curated using IGV^61^. Commercial genetic testing reports provided additional benchmarks for variant quality. If reports conflicted with our pipeline, we rescued variants if we identified supporting reads in the sequence alignment. All mutations required a minimum variant allele fraction greater than 10% to remain included. No silent mutations were reported.

#### SNP array CNV

Each model underwent analysis using the Illumina Infinium Omni2.5Exome-8 v1.3 or v1.4 or the Affymetrix SNP 6.0 array. These samples were processed according to PDXnet specifications^1^ using the tumor-only procedure outlined by recommendations in ASCAT^62^. To account for median-centered differences between the Affymetrix and Illumina chip, we calculated platform-specific thresholds for amplifications and deletions (0.9 and −0.9 for Affymetrix CN ratios and 0.6 and −0.6 for Illumina CN ratios; Extended Data Fig. 5). The gene set chosen for Fig. 1a was curated from a set of four breast cancer or pan-cancer publications^63,64^ as well as sentinel genes, that is, genes that represent a larger genomic region, from public and commercial sources, including FoundationOne, cBioPortal and Ambry Genetics Corporation. These extra genes include *FGF3*, *CDK12*, *NOTCH2*, *H3P6*, *SIPA1L3*, *ADCY9*, *FAM72C*, *SDK2*, *CDK18*, *STX4*, *TNFRSF10C*, *NKX3-1*, *LYN*, *JAK1*, *JAK2*, *CD274*, *PDCD1LG2*, *ERBB4*, *BARD1*, *BRCA1*, *BRCA2*, *BRIP1*, *CDH1*, *CHEK2*, *MRE11A*, *MUTYH*, *NBN*, *NP1*, *PALB2*, *RAD50*, *RAD51C and RAD51D*.

#### RNA-seq

Due to the extended period of data collections, two different library preparation strategies were used for RNA-seq preparation: Illumina TruSeq RNA Library Preparation kit v2 (RS-122-2001 and RS-122-2002) and the Illumina TruSeq Stranded Total RNA kit with Ribo-Zero Gold (RS-122-2301 and RS-122-2302). Samples were processed and sequenced by the High-Throughput Genomic Sequencing Core at the Huntsman Cancer Institute. Due to the variable technical approaches for obtaining RNA transcript abundance data, downstream batch correction strategies were required to correct for technical differences between platforms (see below).

#### Transcript abundance estimations

Like WES, all RNA-seq samples were processed as part of the CGC-SevenBridges cloud interface in accordance with PDXNet-approved pipelines^54^ (see Code availability). Both the transcript-level and gene-level abundance results files capture RSEM expected counts, transcripts per million and fragment per kilobase per million estimates. For this manuscript, RSEM estimates were used for in-depth sample correlations and comparative RNA-seq analyses.

#### RNA-seq count normalization correction strategies and analysis

RNA-seq transcript abundance was used for PAM50 gene-based classification and in-depth sample correlation. We implemented the following steps to normalize transcript abundance. First, we normalized RSEM expected counts using DeSeq2 (ref. ^65^). Second, normalized counts were offset by 1 and log_10_ transformed. For gene-level comparison analysis, we performed root mean squared scaling for each gene. For correlation analysis, we removed likely housekeeping genes by estimating the s.d. of transcript abundance for each gene. We removed genes with a s.d. less than 0.1, which left 80% of the dataset for pairwise Spearman correlations. The resulting correlation matrix was then leveraged to estimate intra- and intersample correlation differences. We used a non-parametric Mann–Whitney *U*-test to compare intrasample correlation estimates (ignoring the diagonal) to the pairwise correlation estimates of other models. Finally, we used ComplexHeatmap R^66^ to display heat maps.

#### Single-cell RNA-seq

Frozen PDX and PDxO samples were dissociated to single cells using the Miltenyi Human Tumor Dissociation kit and GentleMACS dissociator. Samples were strained and loaded onto the 10x Genomics Chromium Controller. The 10x Genomics Single Cell 3′ Gene Expression Library Prep v3 was used. Following library quality control, 2 × 150-base pair (bp) sequencing was performed using Illumina’s NovaSeq6000. We used CellRanger (version 3.0.2, 10x Genomics) to align reads to GRCh38-3.0.0 and hg-mm10, to remove low-quality cells and mouse cells and to quantify gene abundance from GRCh38 bam. PDX and PDxO single-cell RNA-seq data were aggregated using the CellRanger command ‘aggr’ with depth normalization. Raw expression data were loaded into R version 3.5.2 and analyzed using the Seurat package version 3.1.0 (ref. ^67^). Criteria for further filtering high-quality cells were (1) cells have more than 2,000 and less than 10,000 genes detected and (2) cells have less than 20% mitochondrial transcript counts. The R package ‘sctransform’^68^ was used for data normalization, and ‘UMAP’ was used for dimensional reduction and graph-based clustering approaches to cluster the cells.

#### Methylation sequencing

Reduced representation methylation sequencing was performed on 500 ng of genomic DNA. Briefly, MspI restriction digest of DNA was performed, Klenow Fragment (3′–5′ exo-) (NEB) was used to fill in the overhangs and leave a 3′-poly(A) extension, and adapters containing methyl-C were ligated. Treatment with sodium bisulfite (EZ DNA Methylation Gold kit, Zymo Research) or enzymatic conversion (Enzymatic Methyl-seq Conversion Module, NEB, E7125L) was used to convert unmethylated cytosine to uracil. The converted library was PCR amplified using *Taq* polymerase and barcoded primers. Equimolar quantities of barcoded libraries were pooled and sequenced to obtain at least 50 million reads per sample on the Illumina HiSeq 2500 or NovaSeq. PhIX control library (Illumina) was included at 20–25% in each sequencing lane to improve cluster identification and base balancing. FastQC v0.11.4 (http://www.bioinformatics.babraham.ac.uk/projects/fastqc/) was used to ensure that the read starts with the MspI recognition sequence and contained adequate base balancing (20–40% A,C,G and T) in subsequent cycles. Adapters were trimmed from the sequencing reads using Trim Galore v0.4.4 (http://www.bioinformatics.babraham.ac.uk/projects/trim_galore/), with options --rrbs and --fastqc. Alignment to the hg19 reference genome was performed using Bismark v0.19.0108 options -bowtie2, --non_bs_mm, -N 1 and --multicore 6. Library quality was considered sufficient if the unique read alignment was greater than 60%, the percent of methylated Cs in the CHH context (where H correspond to A, T or C) was less than 3% and more than 700,000 CGs in the genome had at least 10 reads. The percent of methylated reads at individual CG positions was quantified using the Bismark Methylation Extractor options --zero_based and --bedGraph. The Pearson correlation in genome-wide methylation between samples was assessed using the cor function and the pheatmap package version 1.0.10 in the statistical software R version 4.0.2.

### *ESR1* mutation testing

Assessment of hotspot *ESR1* mutations in PDX tumors was conducted with ddPCR. Briefly, genomic DNA and total RNA were extracted from each tumor using the Qiagen AllPrep DNA/RNA kit. cDNA was synthesized from 500 ng of RNA using a PrimeScript RT Reagent kit. A reaction mixture was prepared by mixing 50 ng of genomic DNA or cDNA templates, ddPCR supermix for probes and corresponding primer/probe sets for specific *ESR1* mutations (Y537S/Y537C/Y537N/D538G), as previously described^69^. Droplets were generated using the QX100 Bio-Rad droplet generator with 20 μl of reaction mixture and 70 μl of droplet generation oil. The *ESR1* ligand-binding domain fragment was amplified in each sample, and signals from wild-type and mutant probes of each droplet were read using the Bio-Rad QX100 droplet reader. Mutation allele frequencies were further calculated using Quantasoft Software (Bio-Rad). Positive- and negative-control droplets were included in each run to exclude potential contamination artifacts and to control for proper gating of alleles. Genomic DNA extracted from genome-edited MCF7 *ESR1*-mutant cells^70^ was used for positive controls of Y537S and D538G detection, and mutant DNA oligonucleotides were used for positive controls of Y537C and Y537N detection. Genomic DNA from MCF7 *ESR1* wild-type cells was used for negative controls.

### Generation of PDxoXs

To generate PDxoXs, Matrigel domes of mature PDxO cultures were disrupted by pipetting and washed with cold PDxO base medium. The organoid pellet was resuspended in Matrigel on ice. Each mouse was injected with 20 μl of Matrigel/organoid mixture into the mammary fat pad, as previously described^47^.

### PDX drug treatments

Treatment regimens are in Supplementary Table 7. For in vivo drug testing, SCID/Bg, NSG or NRG mice were implanted orthotopically with PDX tumor fragments. NRG mice were used for most experiments with cytotoxic chemotherapy due to their increased tolerance for DNA damage relative to SCID mice. When tumors reached ~100 mm^3^ in size, drug treatment (or vehicle control treatment) was initiated. Tumor size was monitored 1–2 times per week, depending on the study. In some cases, treatment was stopped to see if tumors recurred and restarted to determine resistance. Some of the in vivo drug testing experiments (docetaxel and navitoclax) were performed by the Patient-derived Xenograft and Advanced *In Vivo* Models (PDX-AIM) Core Facility of Baylor College of Medicine (M.T.L., Director) in SCID/Bg mice as a way to cross-validate results between PDxO drug responses and PDX responses.

### Materials availability

PDX models and organoids will be made available to the community as per standard practice (for example, under material transfer agreement), and requests can be initiated by contacting the corresponding authors. Exceptions might include samples with limited material, such as original biopsies.

### Statistics and reproducibility

Specific statistical tests are listed in each figure legend, along with the number of samples assessed. All data points were distinct; no repeated measures were used. Data distribution was assumed to be normal, but this was not formally tested. Statistics used for genomics analysis and drug response assays are described in detail in the Methods. No statistical methods were used to predetermine sample sizes; our sample sizes are similar to those reported in previous publications and by PDXNet consortium standards^54^. Mice were randomized to treatment groups. For organoid experiments, organoids were randomly aliquoted to wells for experiments. Investigators were not blinded to the studies, but data were analyzed by multiple investigators, including those not involved in the experiment. Replicates for each experiment are described in the figure legends. If not specified, the experiment was performed once with the presented number of data points. IHC images displayed in the figures are representative of the respective PDX, PDxO or PDxoX line. No data were excluded, with a few exceptions where technical errors occurred during an experiment, rendering data unusable (for example, a Matrigel dome detached).

### Reporting Summary

Further information on research design is available in the Nature Research Reporting Summary linked to this article.

## Supplementary information

Supplementary Information Supplementary Figs. 1–45.

Reporting Summary

Supplementary Table 1 Supplementary Tables 1–7.

## Data Availability

WES, SNP array CNV data and RNA-seq data reported in Figs. 1 and 5 are available to authorized users in the NIH database of Genotypes and Phenotypes (dbGaP) repository under the accession number phs002479.v1.p1. Data from four individuals were excluded for posting raw data due to IRB language permitting their use in research but not public dissemination of genomic data (HCI-025, HCI-026, HCI-027 and HCI-031). Access to data will be granted following registration in the dbGaP system as an approved user with an eRA Commons account and completion of the online data access request process. Cell line and human-derived model DNA methylation data are available at the Gene Expression Omnibus (GEO) under accession codes GSE152202 and GSE186747, respectively. Source data are provided with this paper. All other data supporting the findings of this study are available from the corresponding author on reasonable request.

## Source data

Source Data Fig. 2 Statistical source data.
Source Data Fig. 3 Statistical source data.
Source Data Fig. 4 Statistical source data.
Source Data Fig. 6 Statistical source data.
Source Data Fig. 6 Raw data.
Source Data Fig. 7 Statistical source data.
Source Data Fig. 8 Statistical source data.
Source Data Extended Data Fig. 1 Statistical source data.
Source Data Extended Data Fig. 3 Statistical source data.
Source Data Extended Data Fig. 4 Statistical source data.
Source Data Extended Data Fig. 9 Statistical source data.

## Code availability

Code used to parse, analyze and visualize high-throughput genomics and drug screening data is publicly available at https://github.com/MHBailey/pdxo_2021_paper. DNA, RNA and SNP array sequence alignment and variant calling were performed on the SevenBridges platform using PDXnet specific workflows. Code is available at the following URLs after acquiring CGC access permissions: https://cgc.sbgenomics.com/u/michael_lloyd/pdxnet-jax-wes-tumor-only-workflow/, https://cgc.sbgenomics.com/u/pdxnet/pdxnet-jax-rna-seq-workflow/ and https://cgc.sbgenomics.com/u/pdxnet/pdx-wf-commit2/apps/#pdxnet/pdx-wf-commit2/snp-array-tumor-only-workflow-for-illumina-infinium-omni-2-5-exo/3, respectively.
