# Supplementary material for: A human breast cancer-derived xenograft and organoid platform for drug discovery and precision oncology
Source: Nat Cancer. 2022 Feb 24;3(2):232–50. doi: 10.1038/s43018-022-00337-6 (PMC8882468; doi:10.1038/s43018-022-00337-6)
Supplement: Supplementary file 1 — Supplementary Figs. 1–45. [file 43018_2022_337_MOESM1_ESM.pdf]

nature portfolio

https://doi.org/10.1038/s43018-022-00337-6

Supplementary information

A human breast cancer-derived xenograft and organoid platform for drug discovery and precision oncology

In the format provided by the authors and unedited

Supplementary Fig. 1

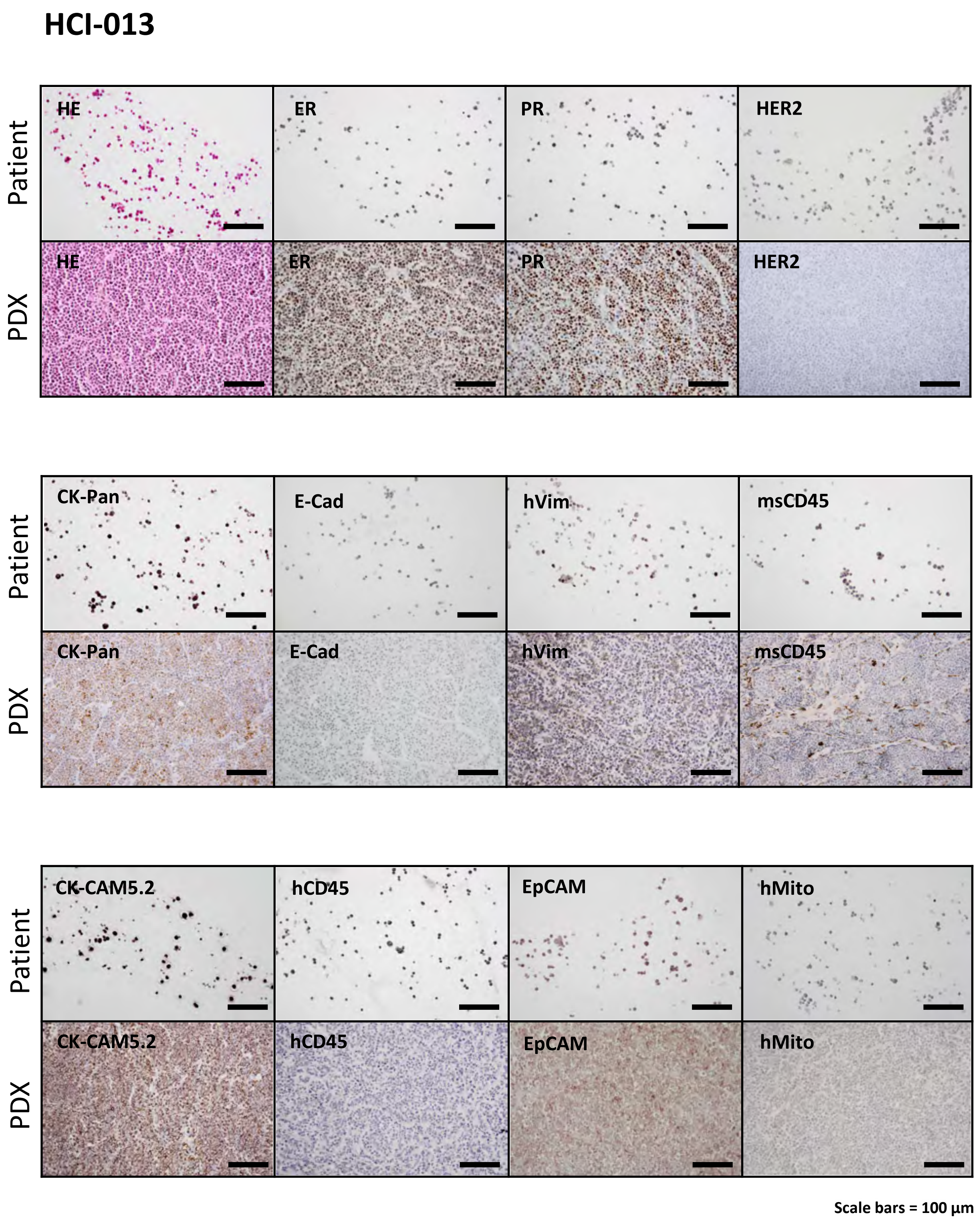

Supplementary Fig. 2

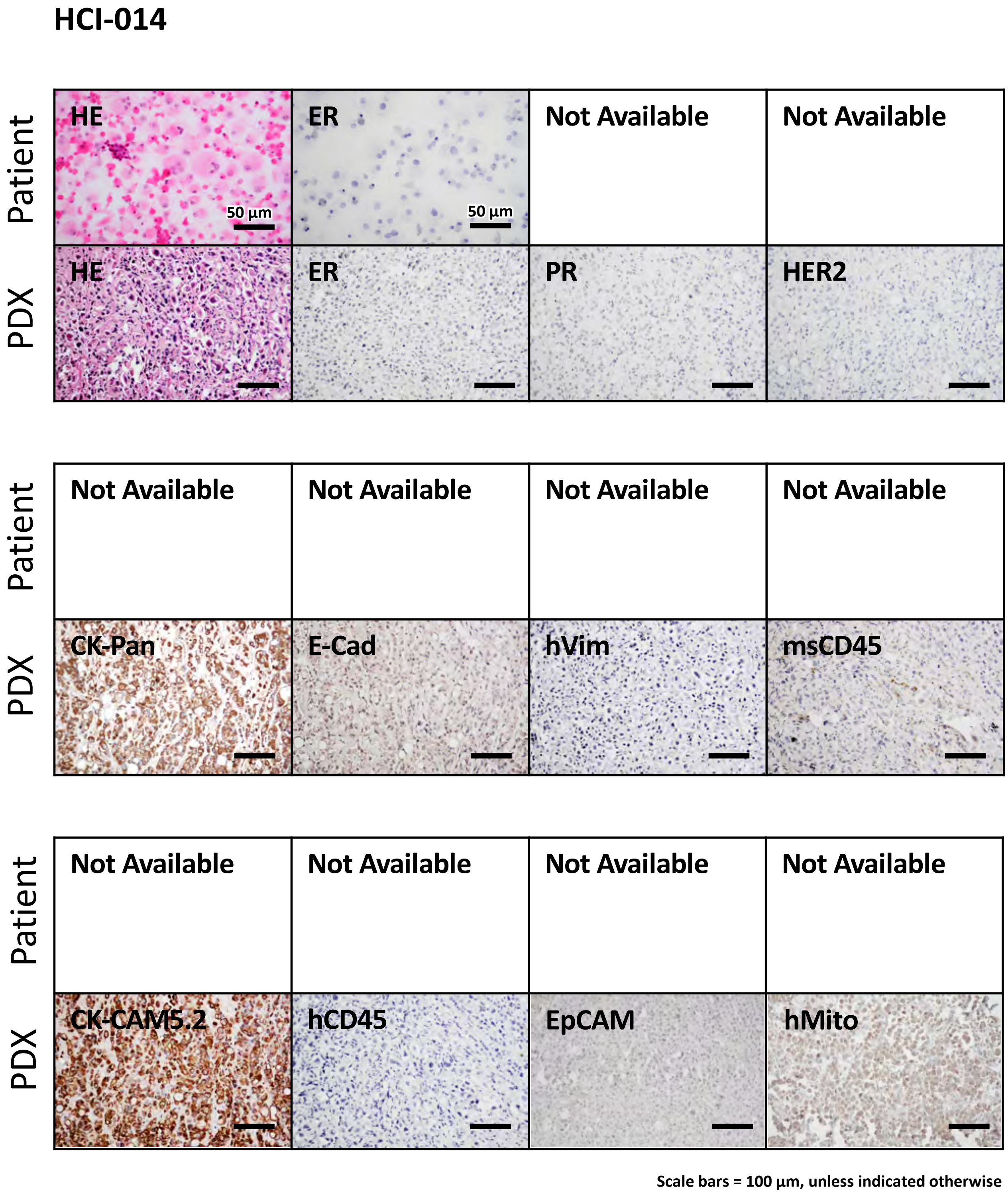

Supplementary Fig. 3

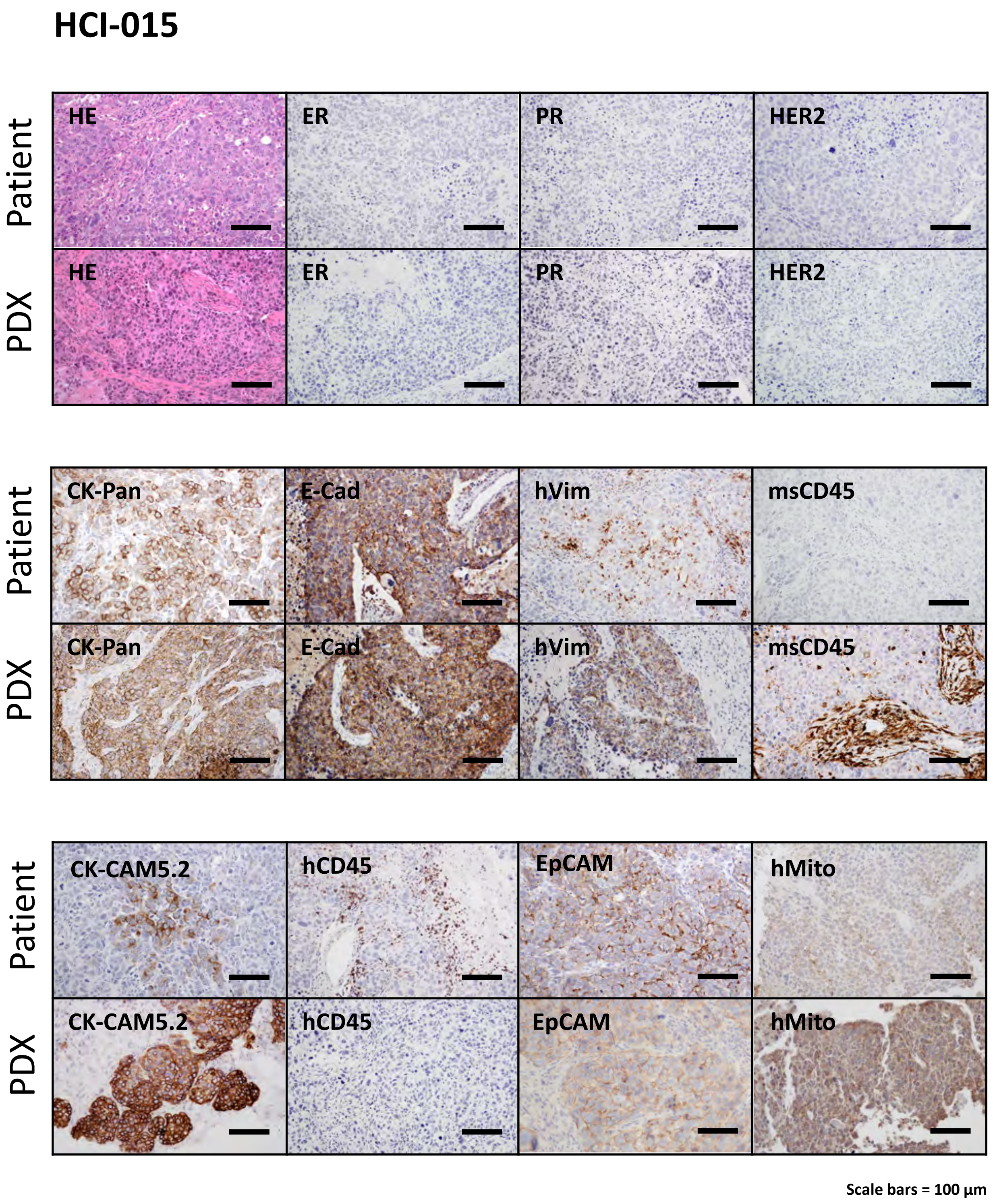

Supplementary Fig. 4

HCI-016

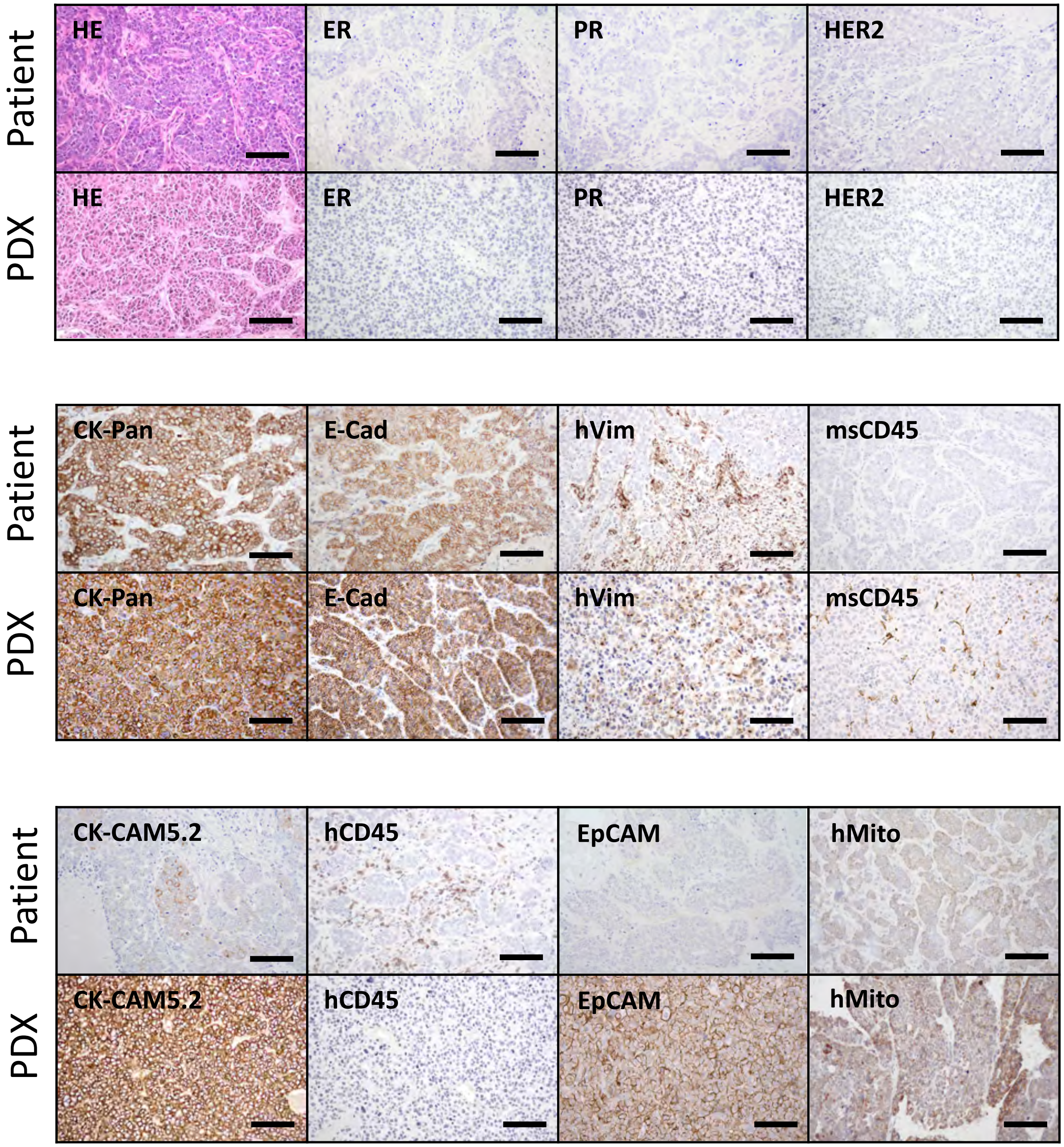

Scale bars = 100 µm

Supplementary Fig. 5

HCI-017

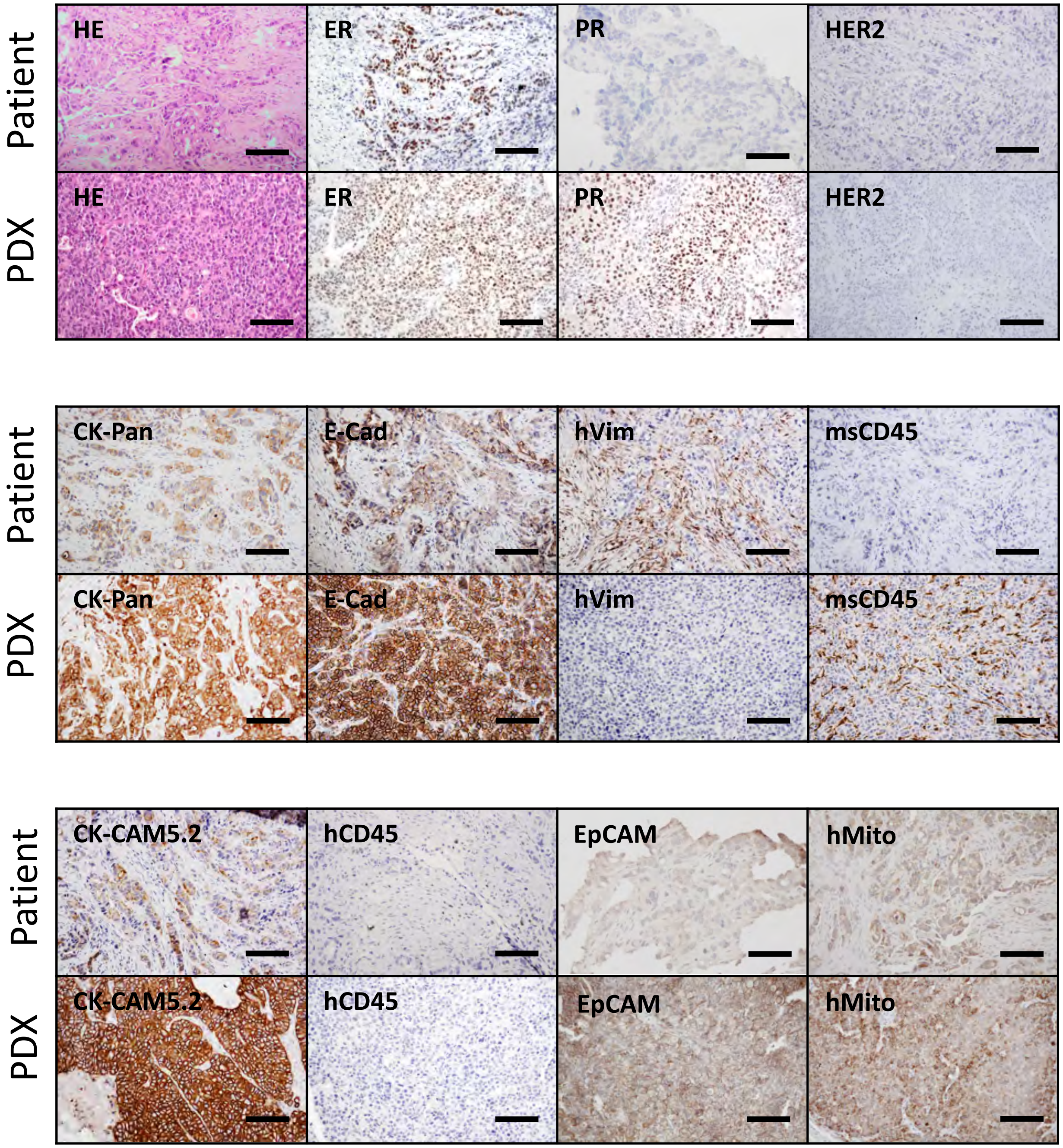

Scale bars = 100 µm

Supplementary Fig. 6

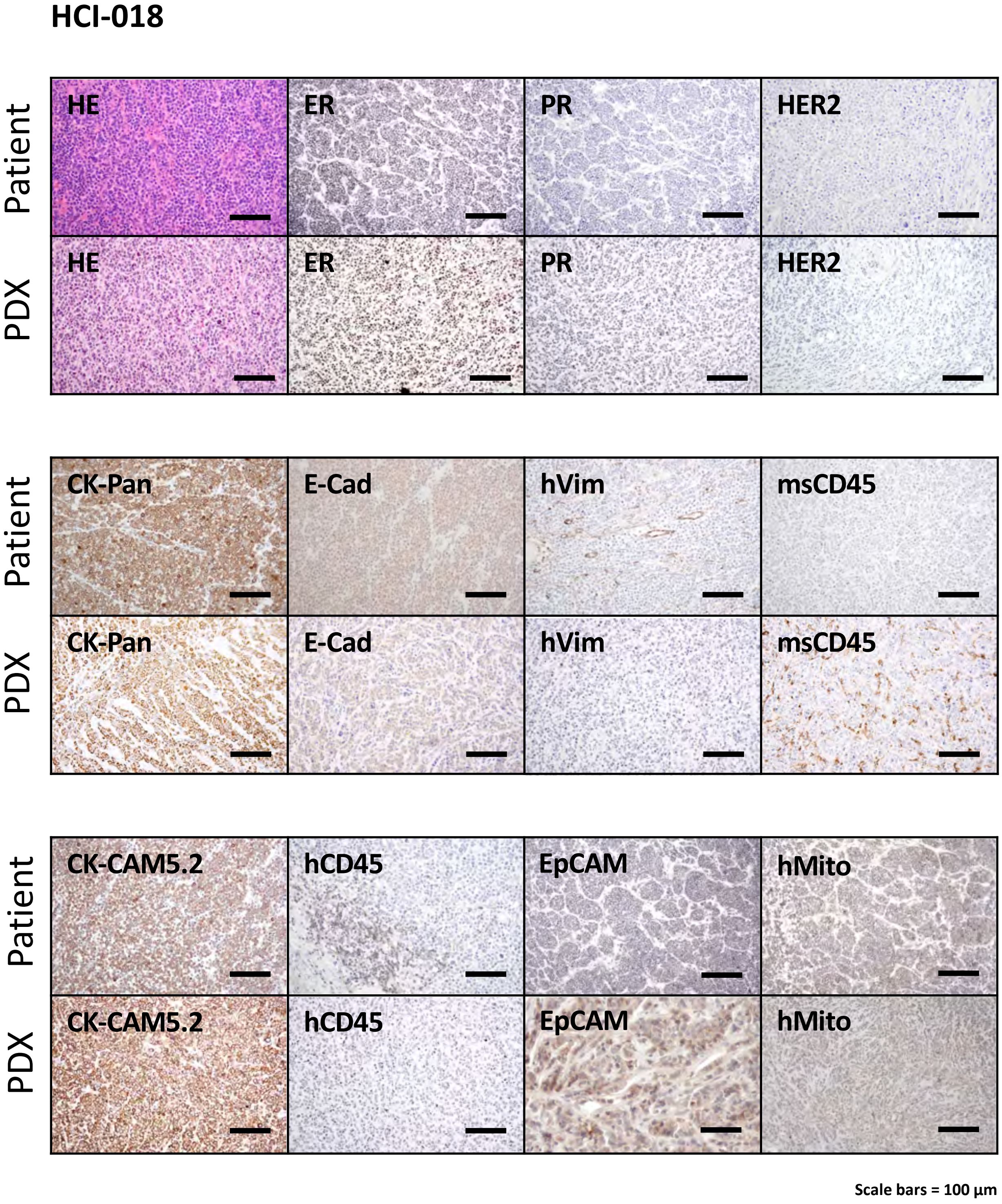

Supplementary Fig. 7

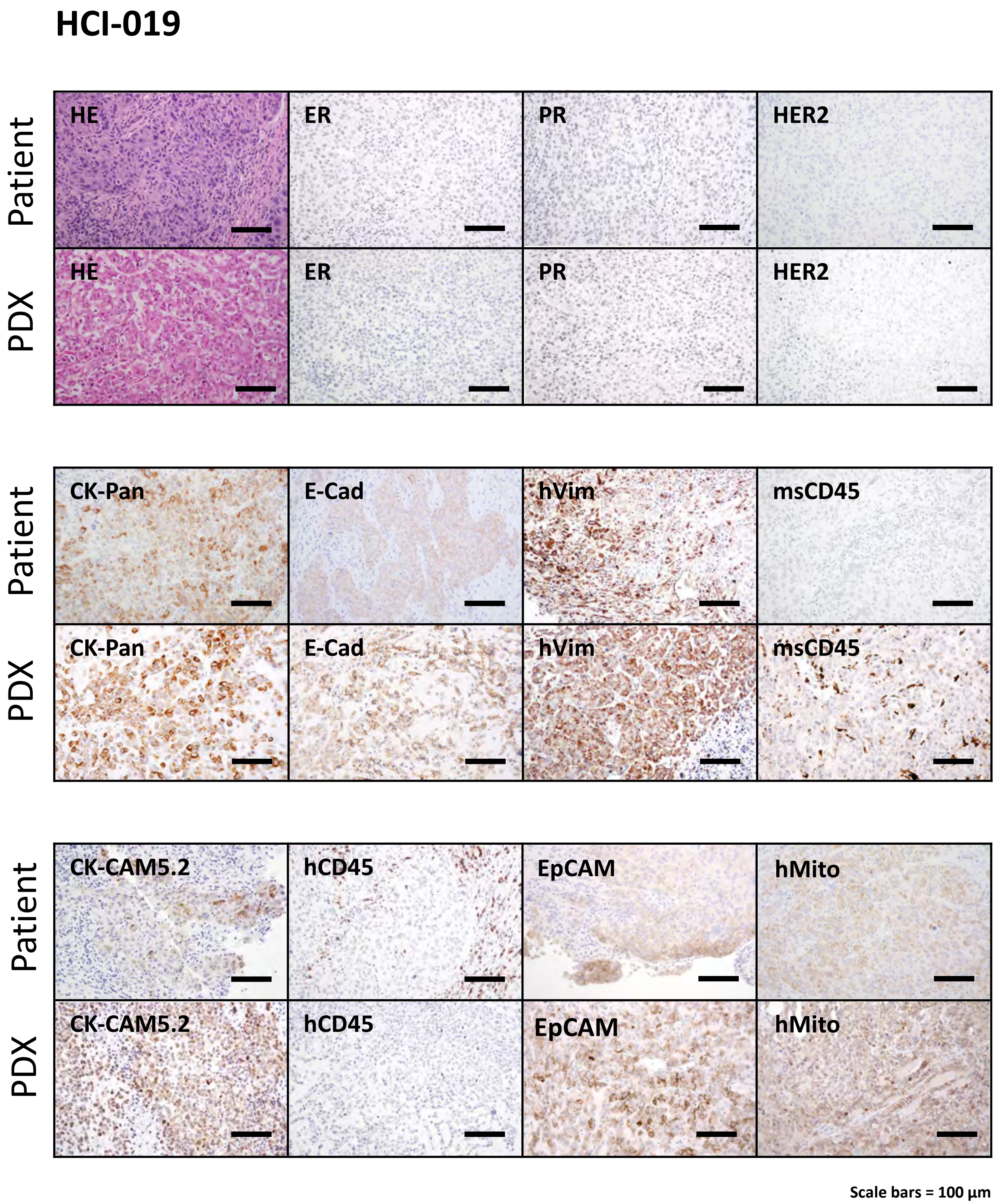

Supplementary Fig. 8

HCI-023

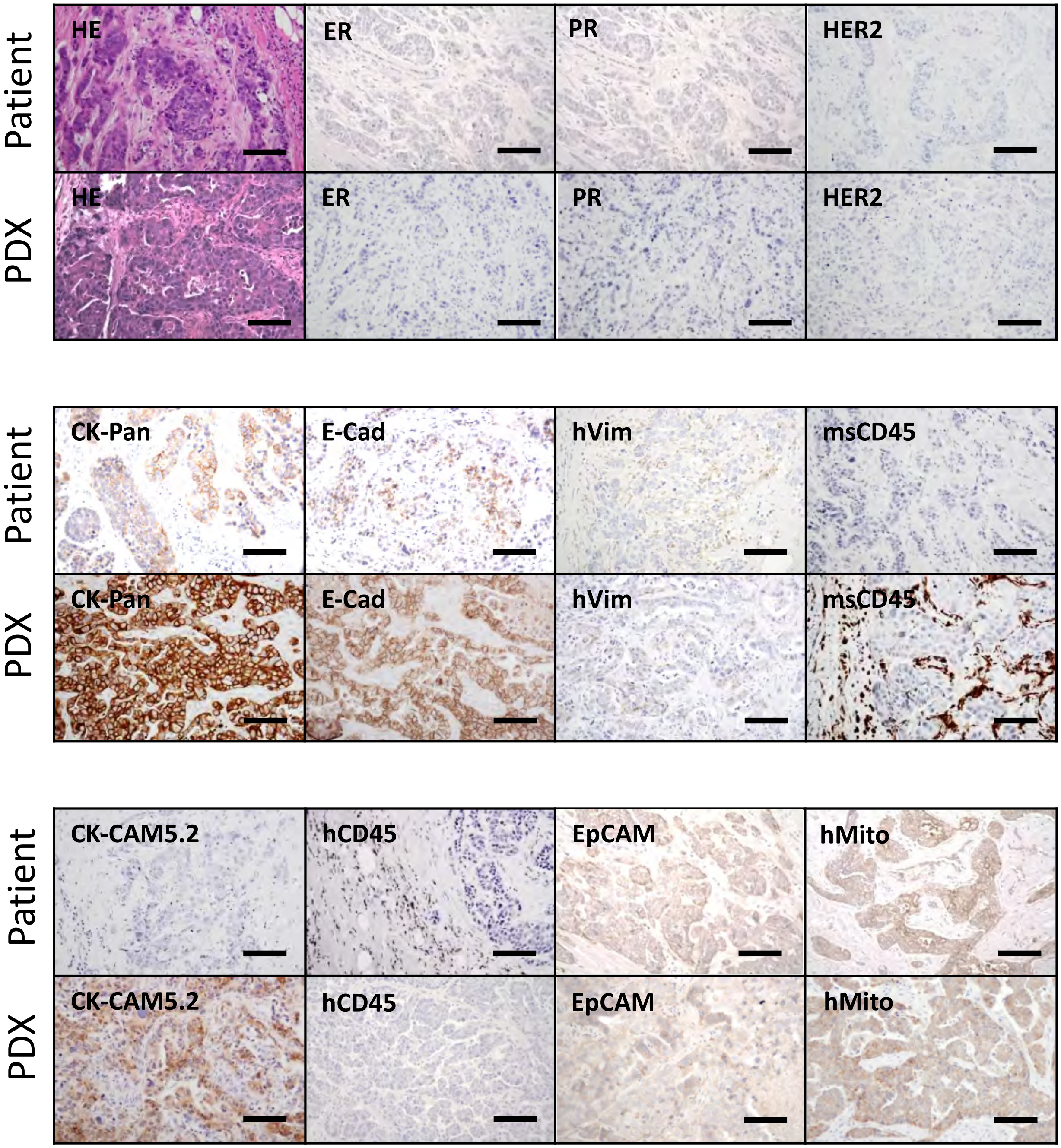

Scale bars = 100 µm

Supplementary Fig. 9

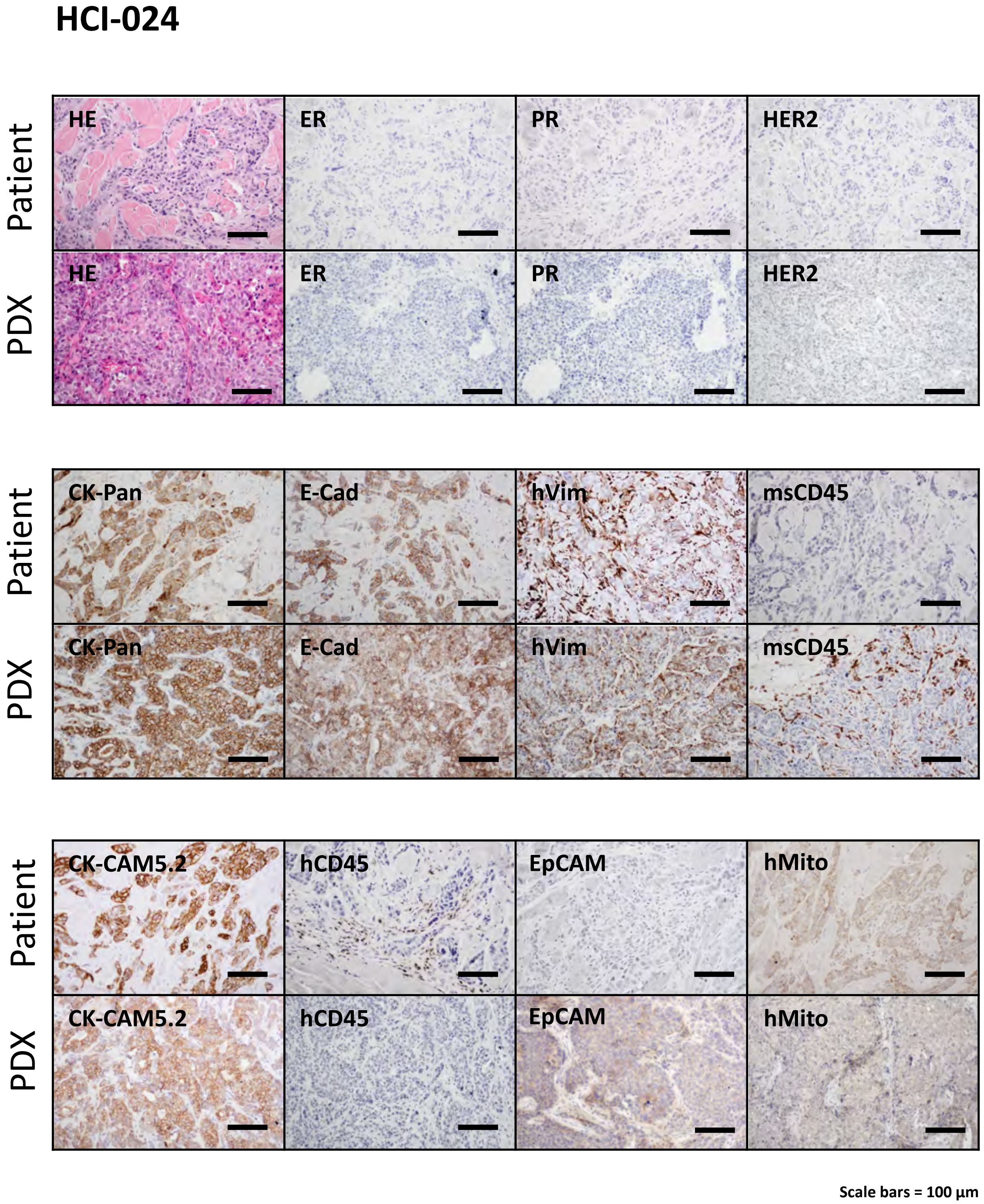

Supplementary Fig. 10

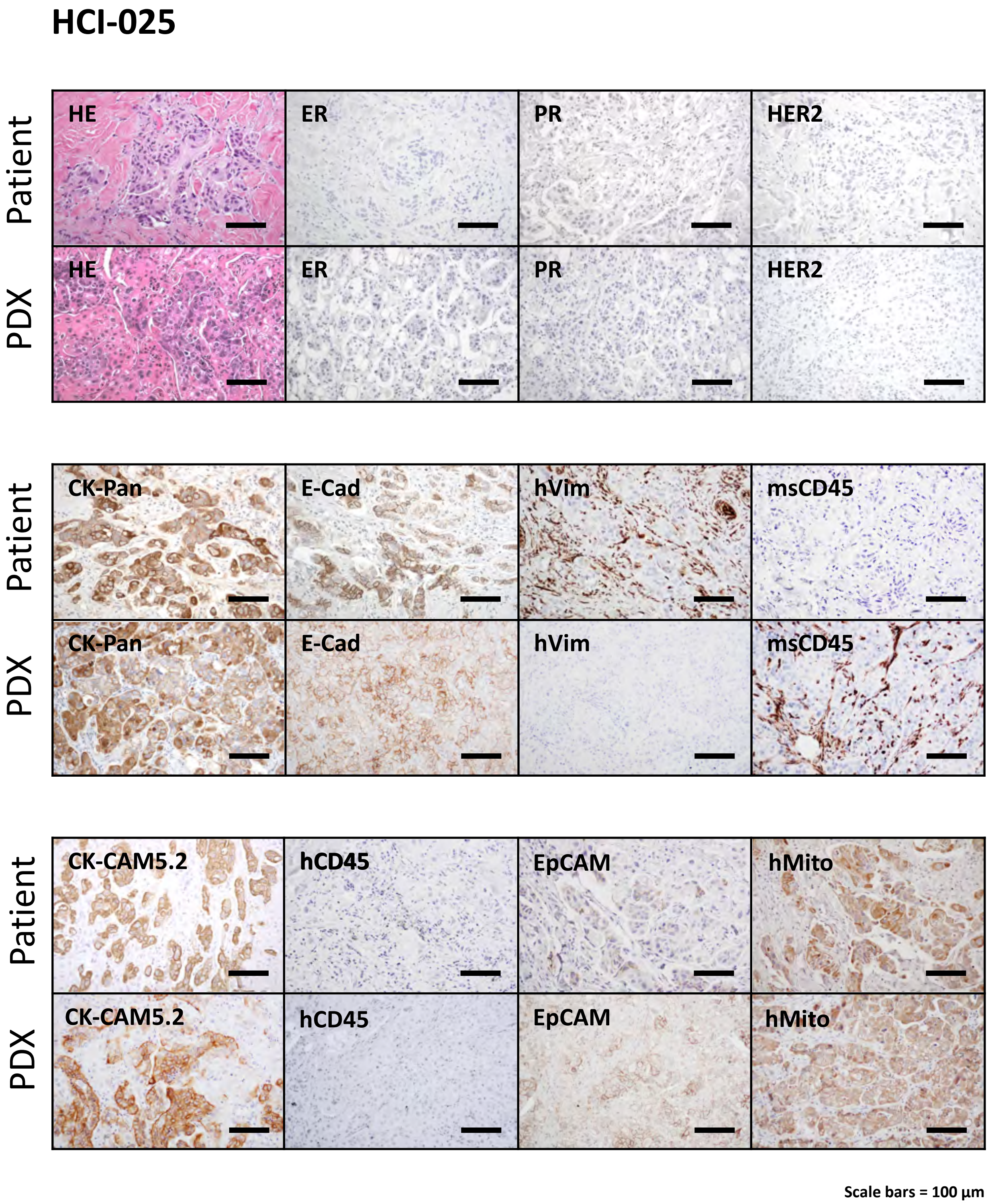

Supplementary Fig. 11

HCI-026

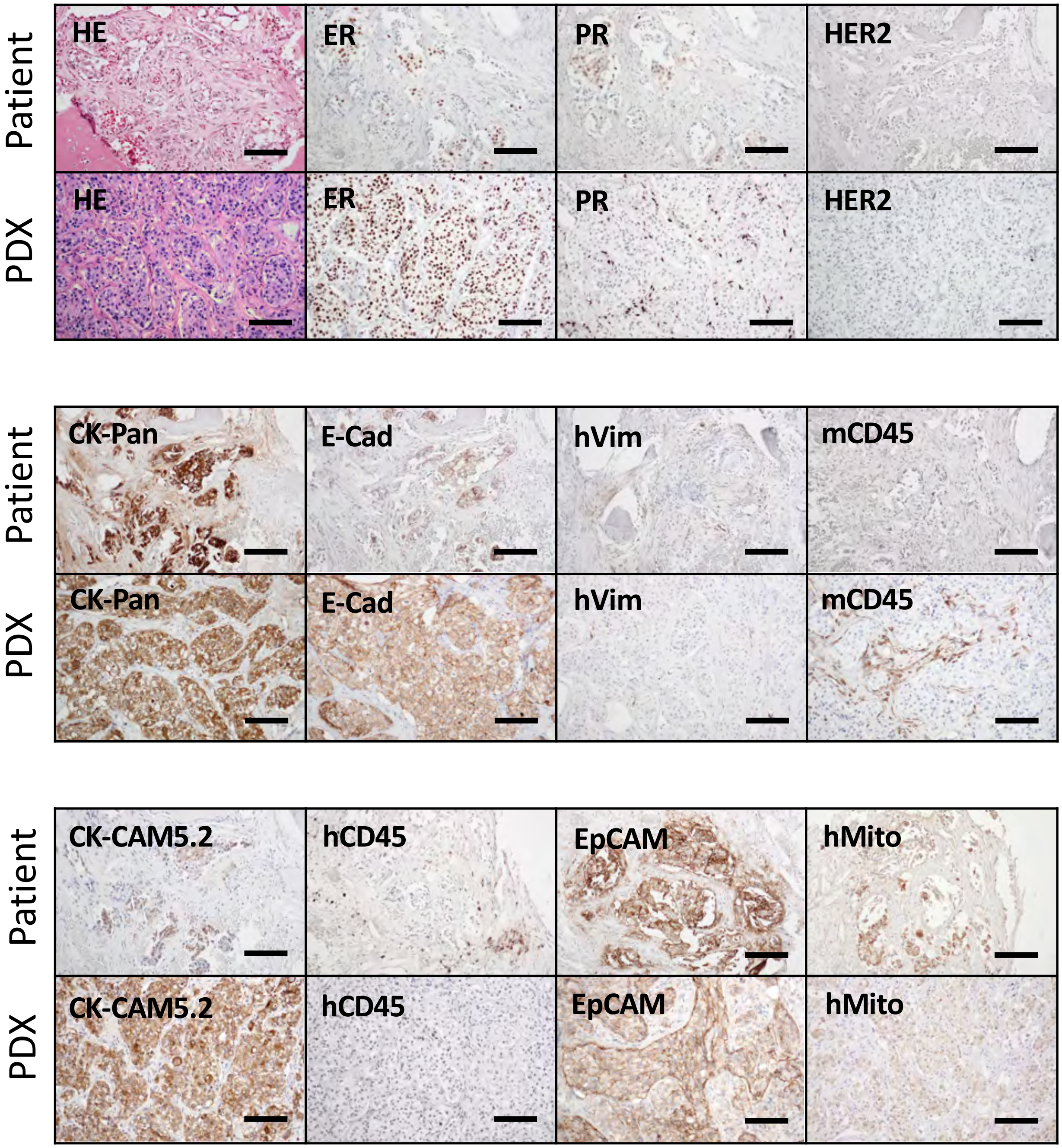

Scale bars = 100 µm

Supplementary Fig. 12

HCI-027

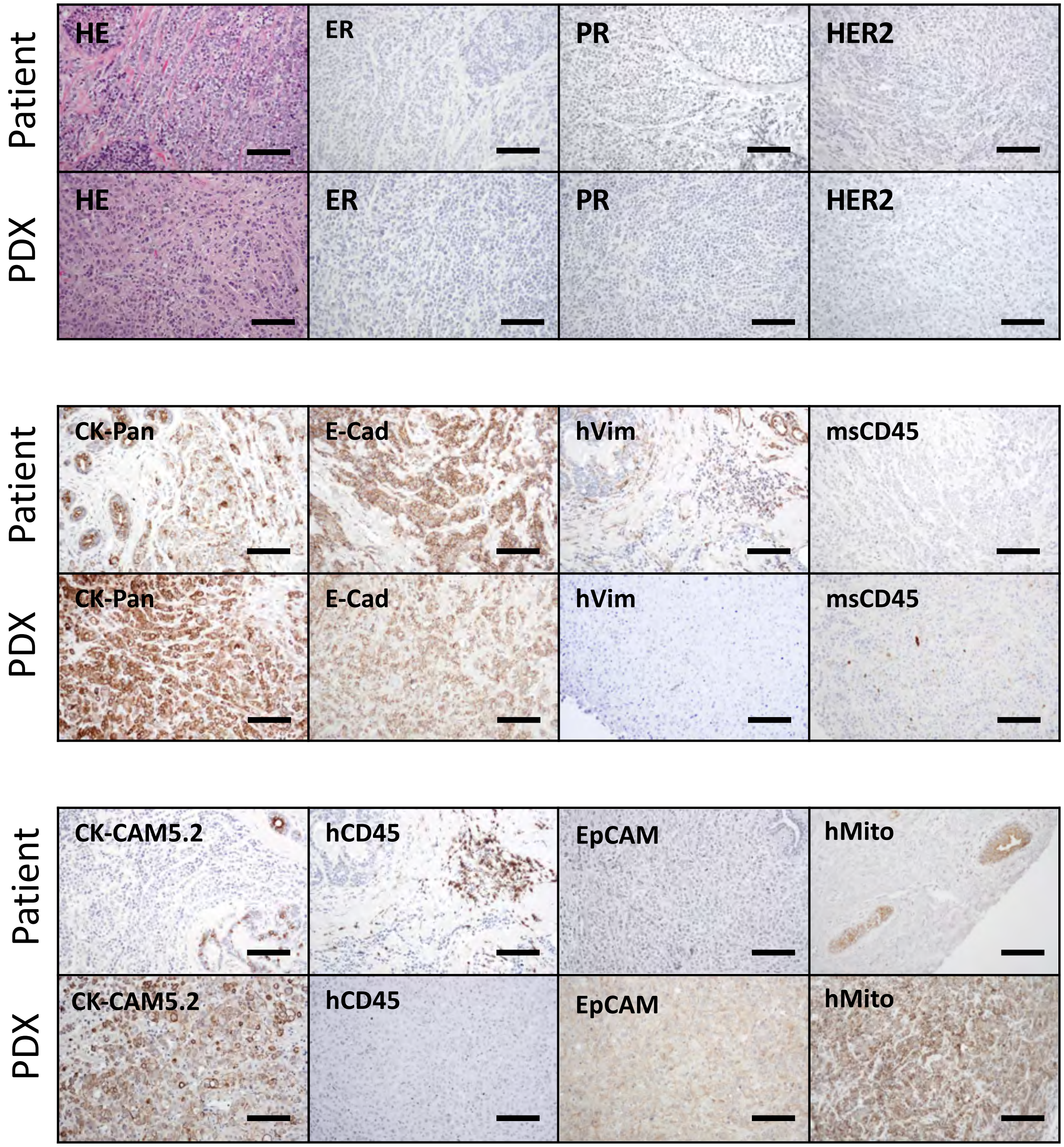

Scale bars = 100 µm

Supplementary Fig. 13

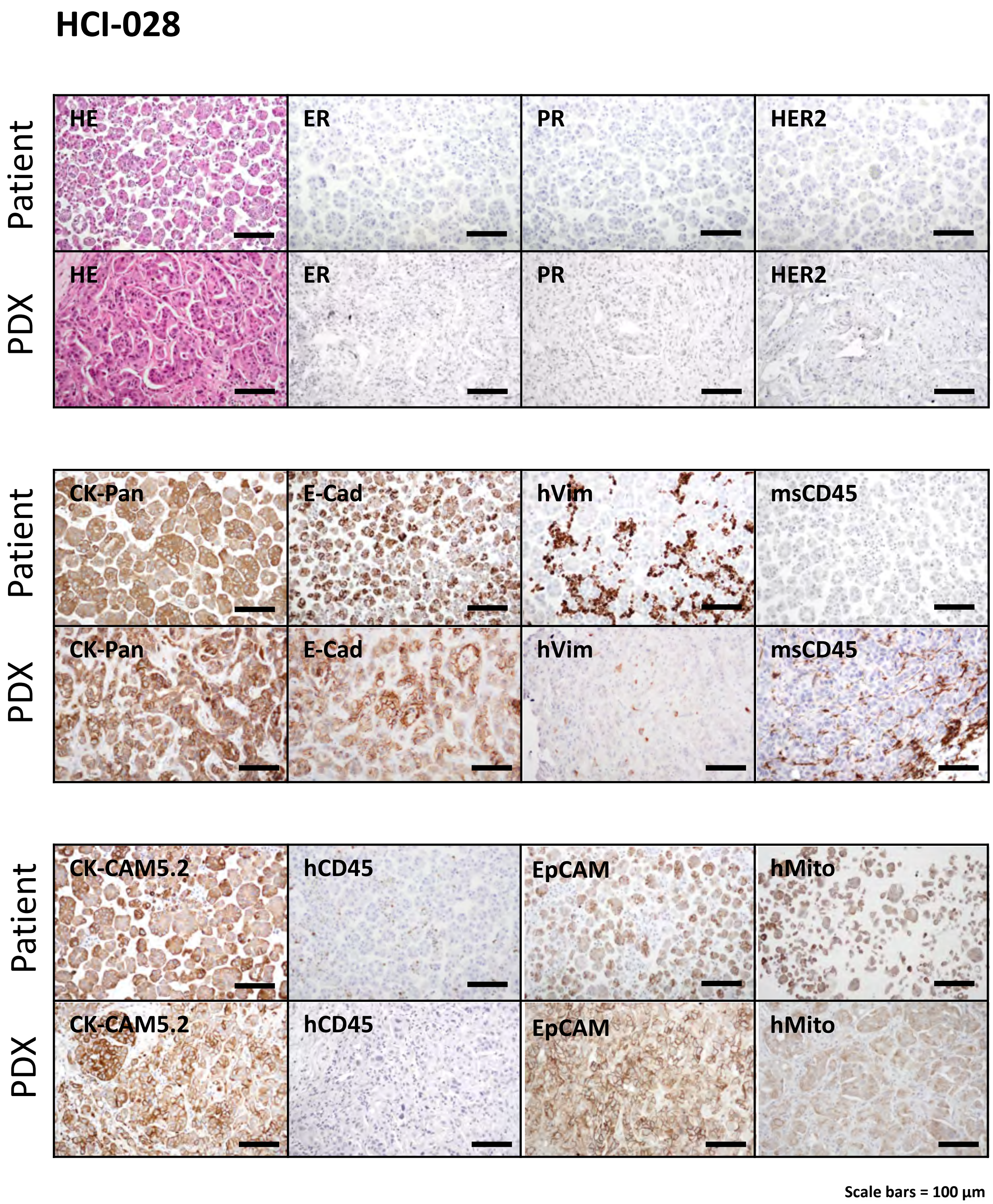

Supplementary Fig. 14

HCI-030

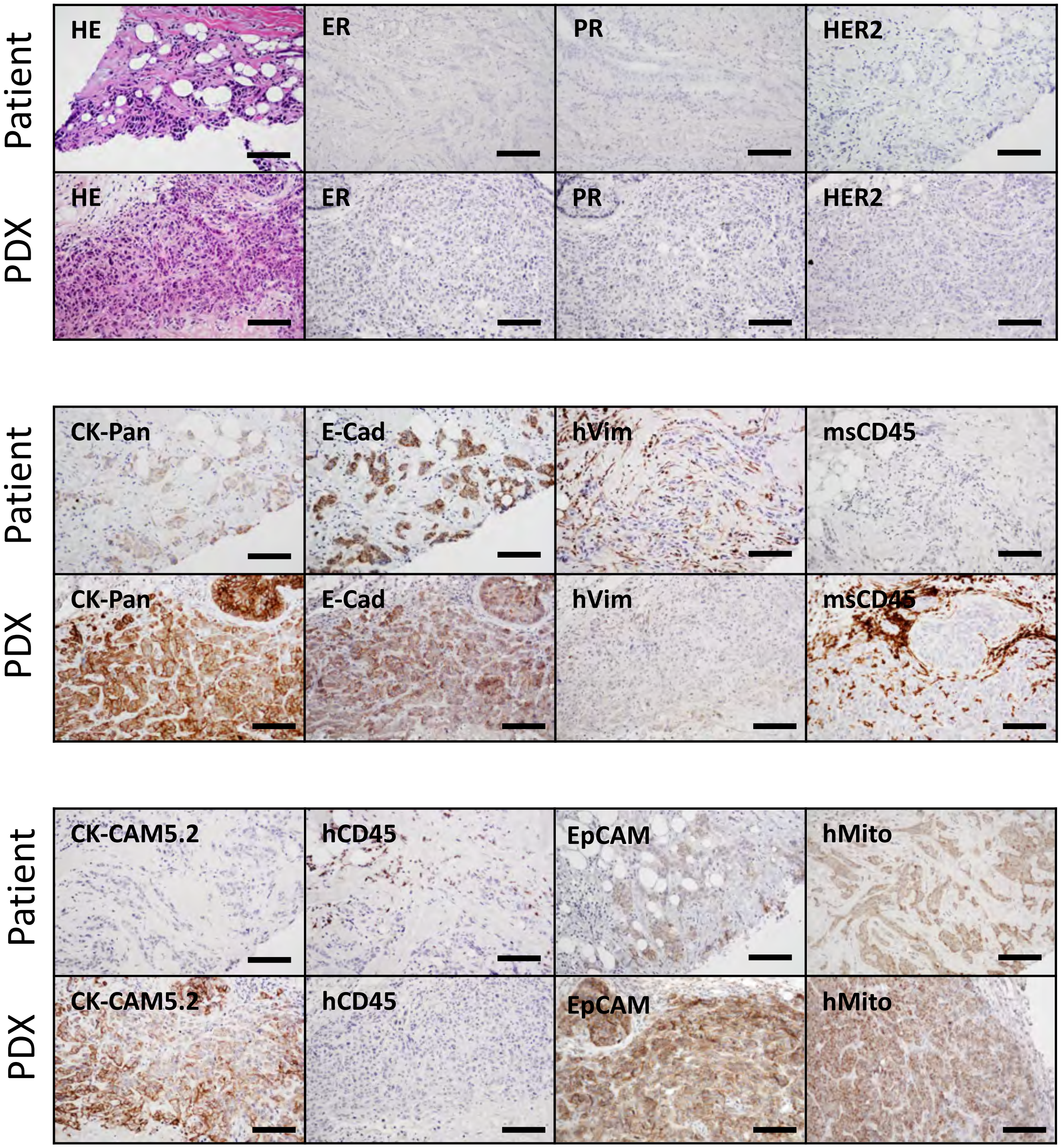

Scale bars = 100 µm

Supplementary Fig. 15

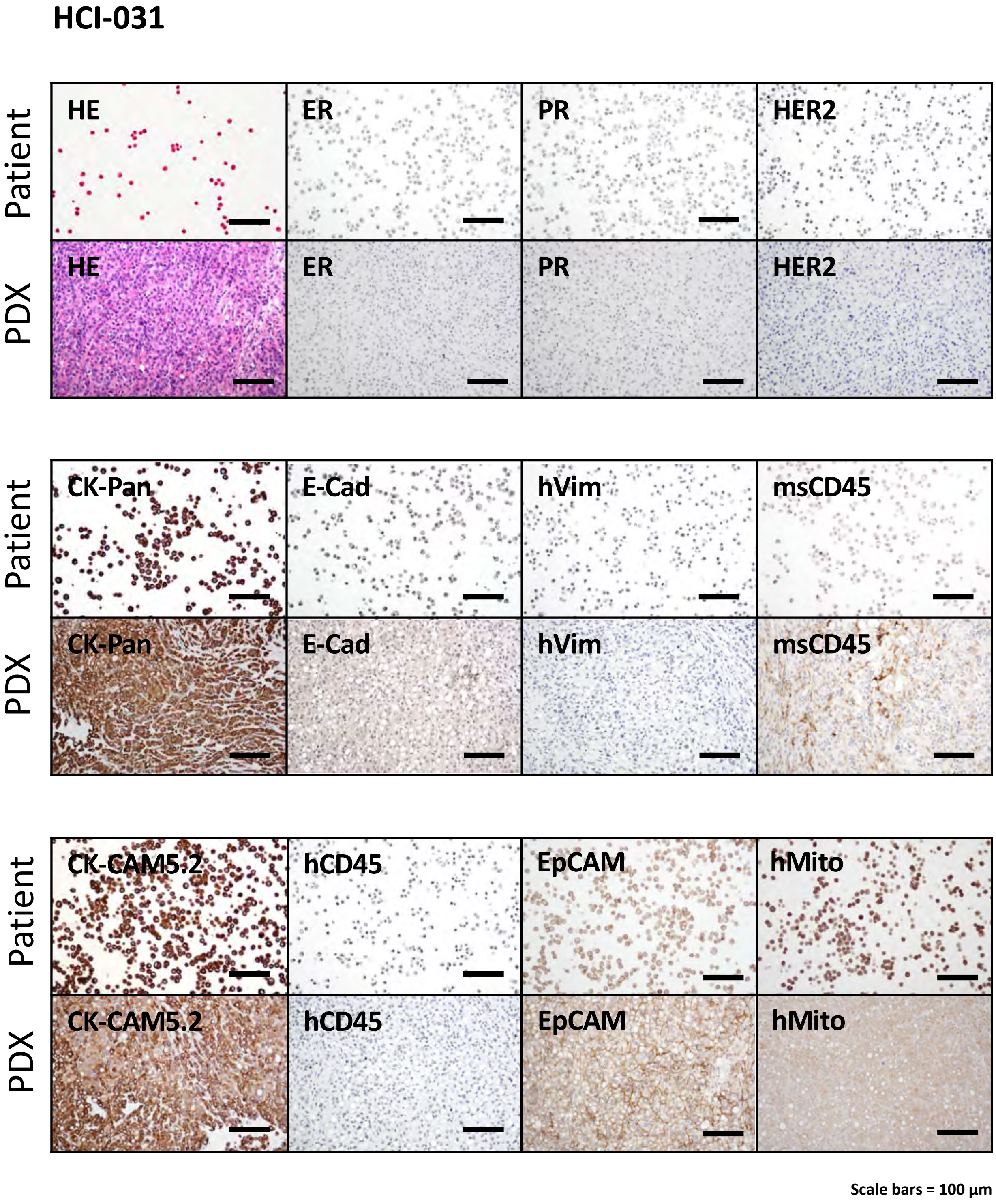

Supplementary Fig. 16

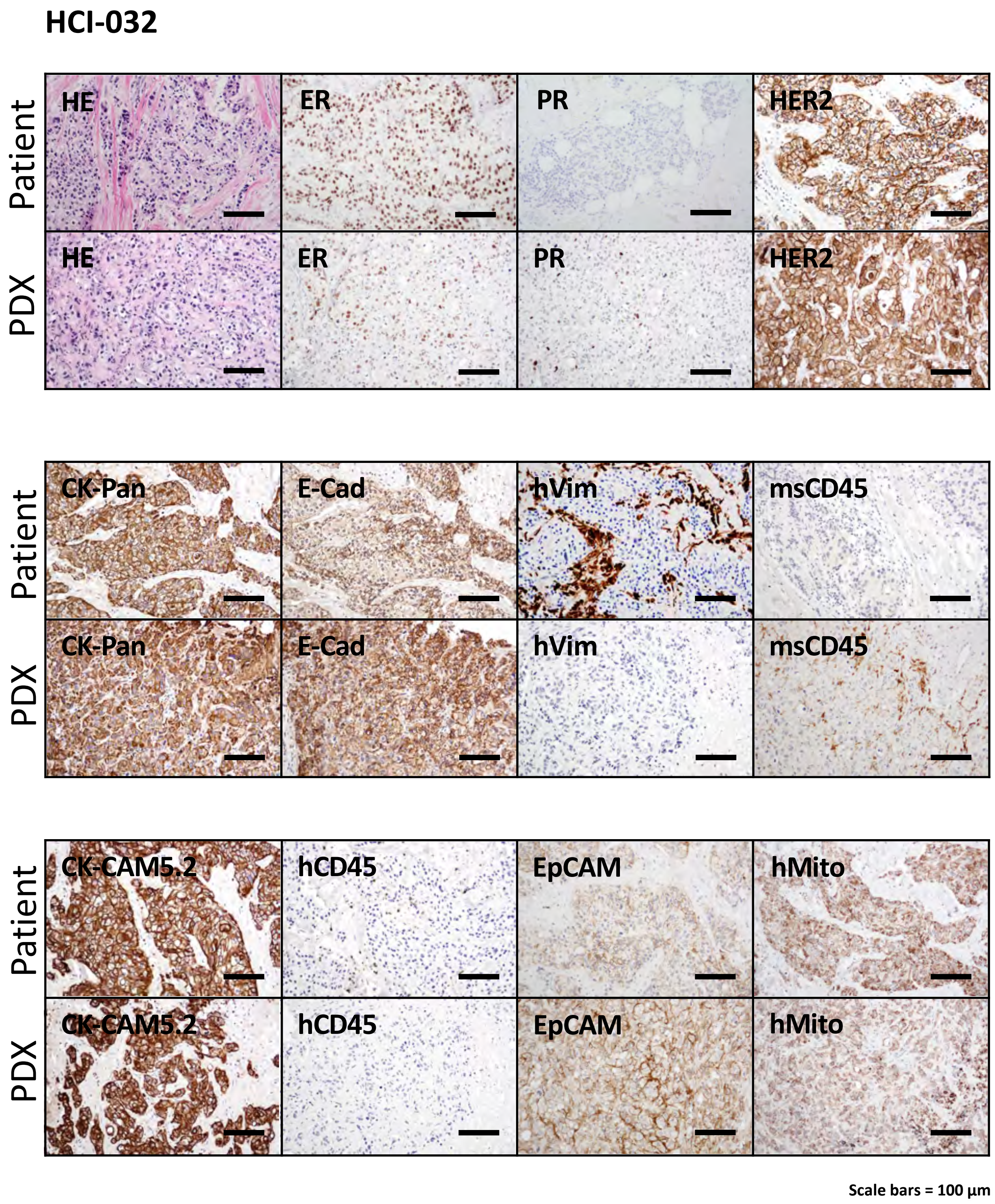

Supplementary Fig. 17

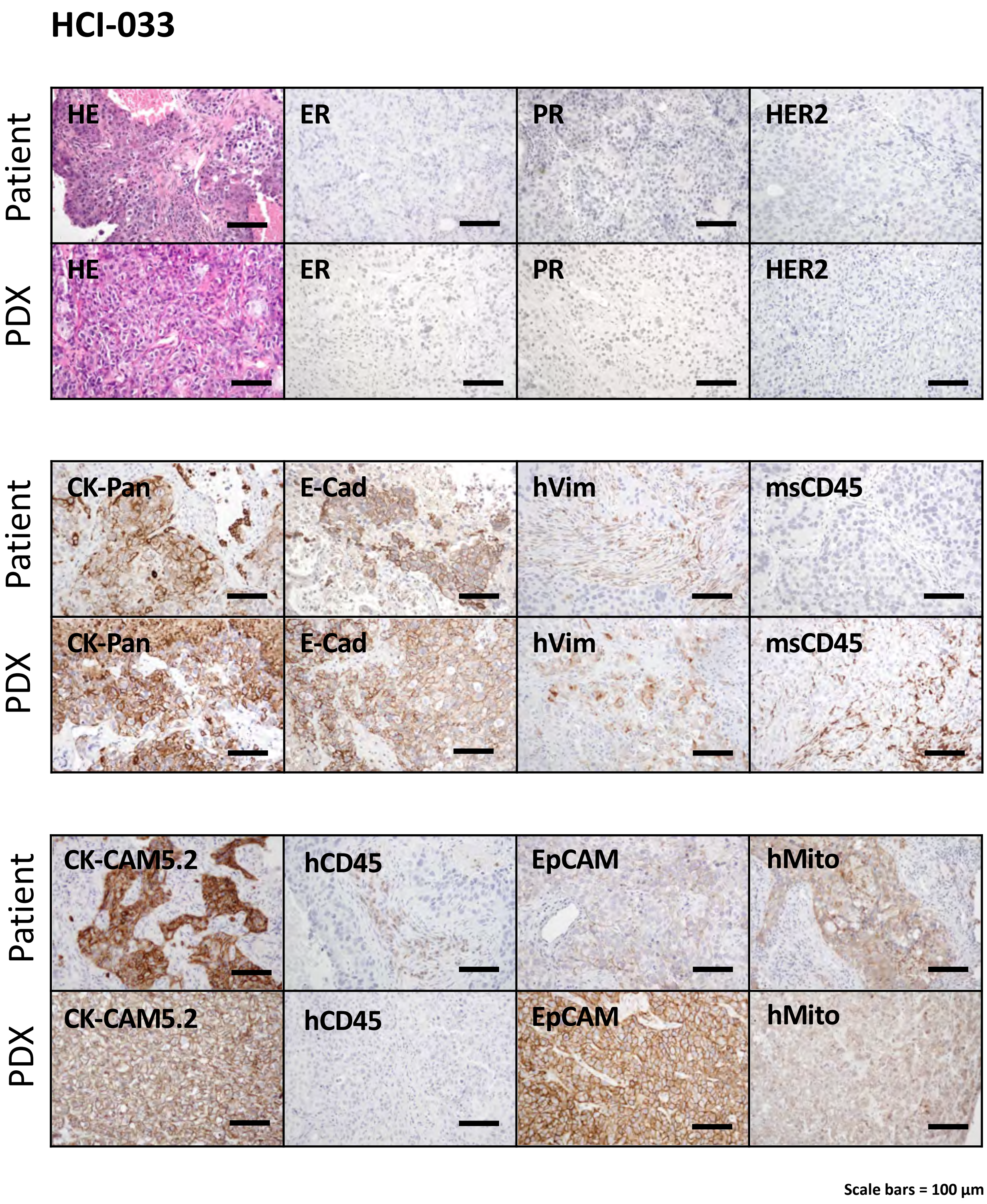

Supplementary Fig. 18

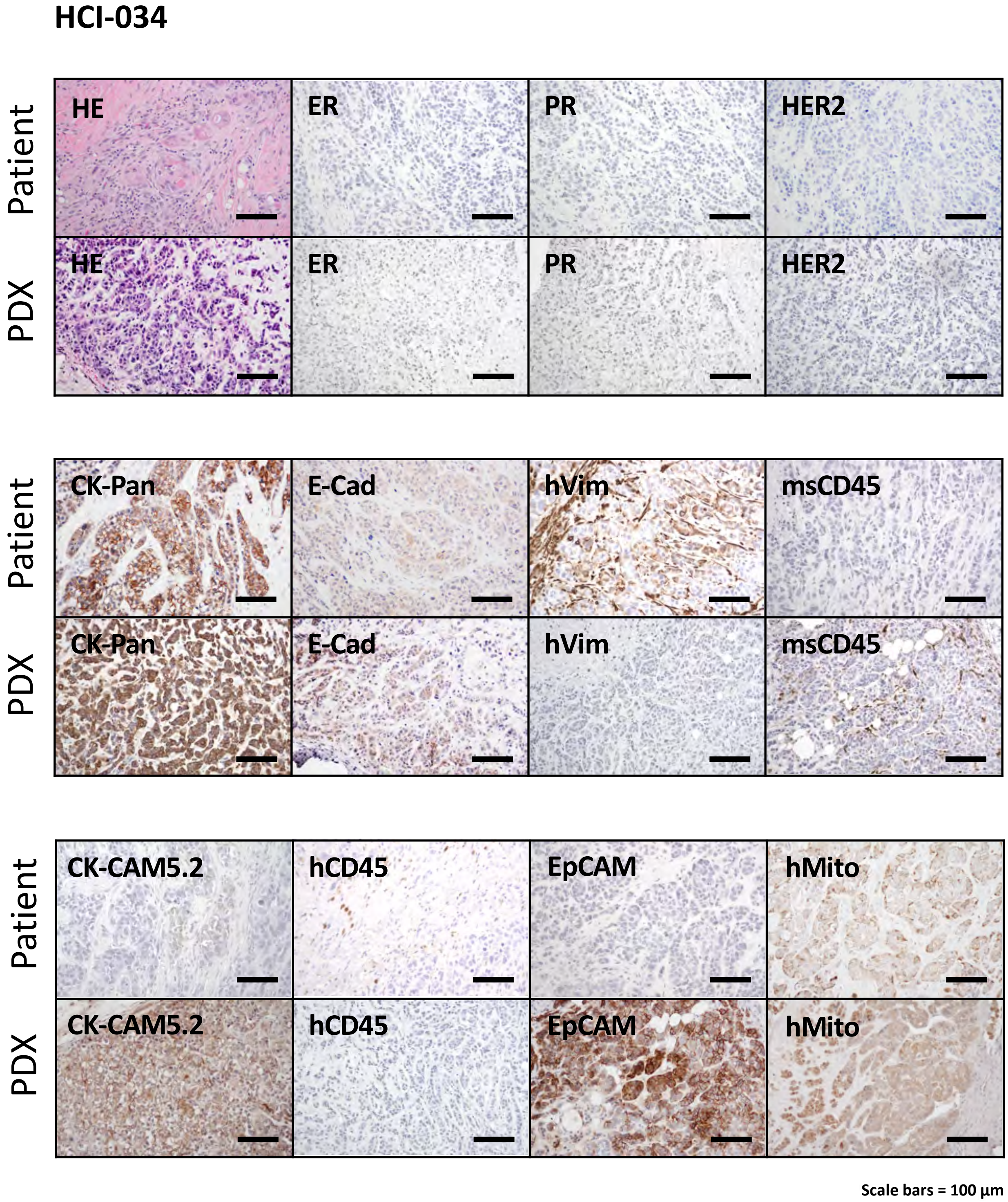

Supplementary Fig. 19

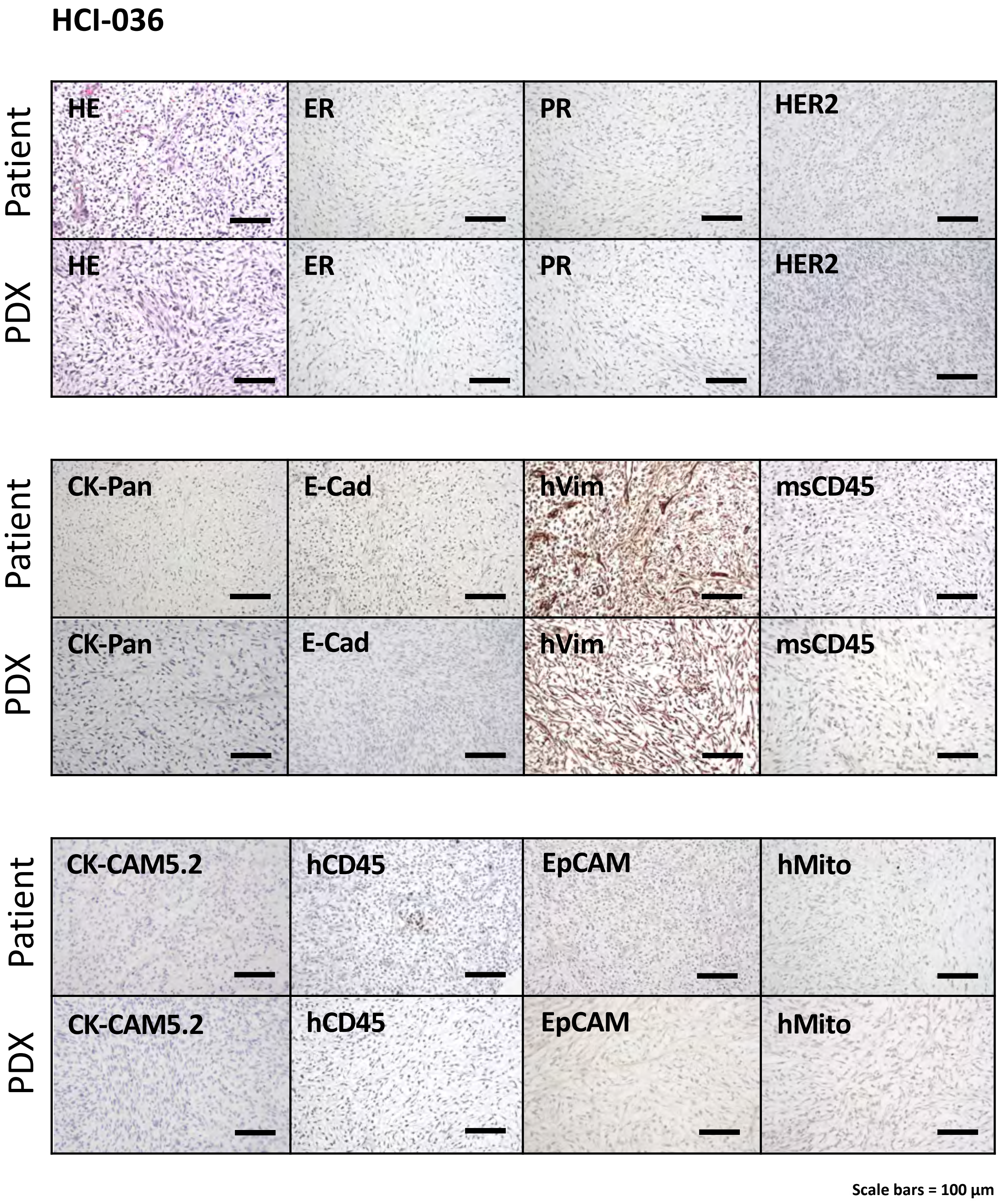

Supplementary Fig. 20

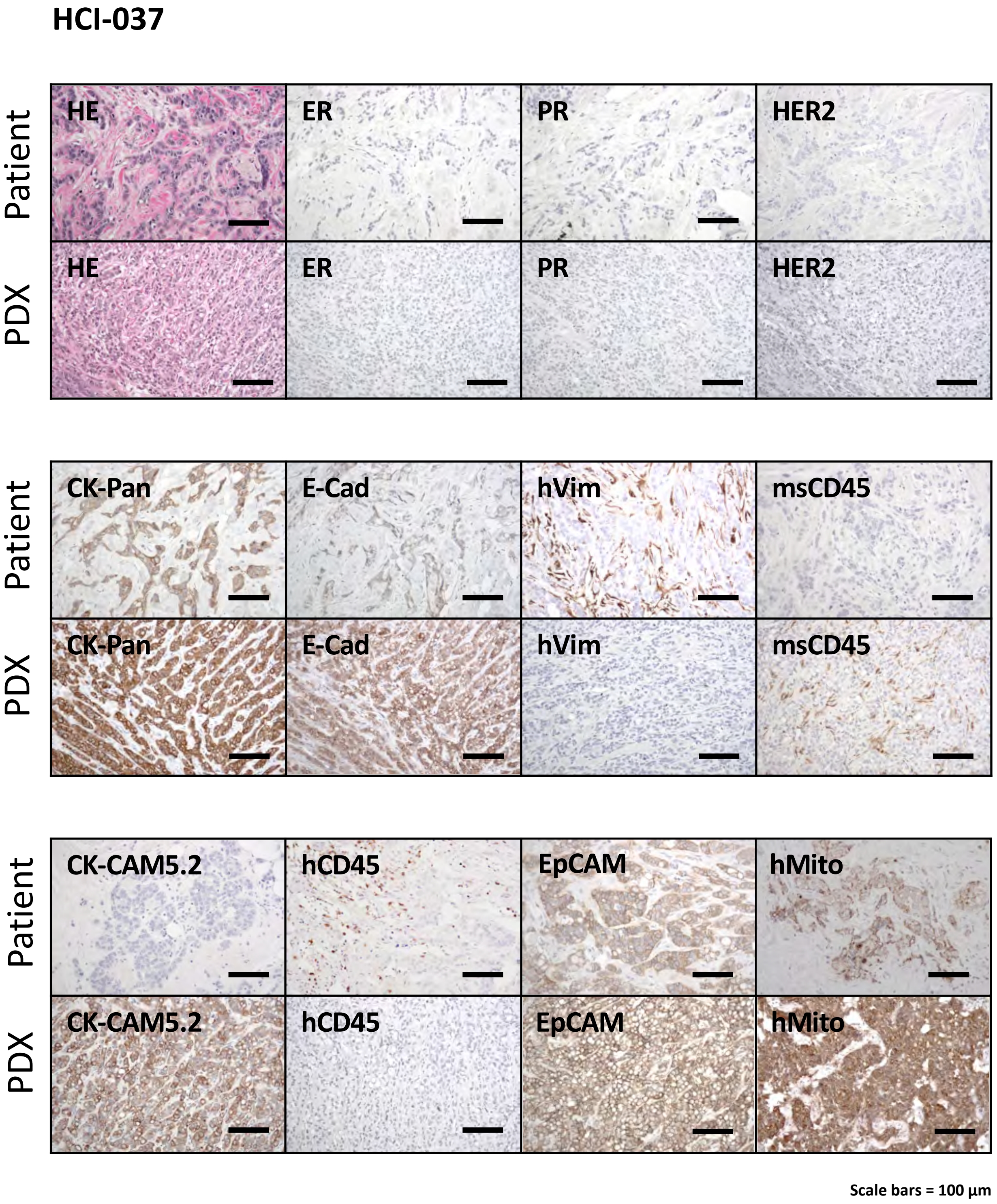

Supplementary Fig. 21

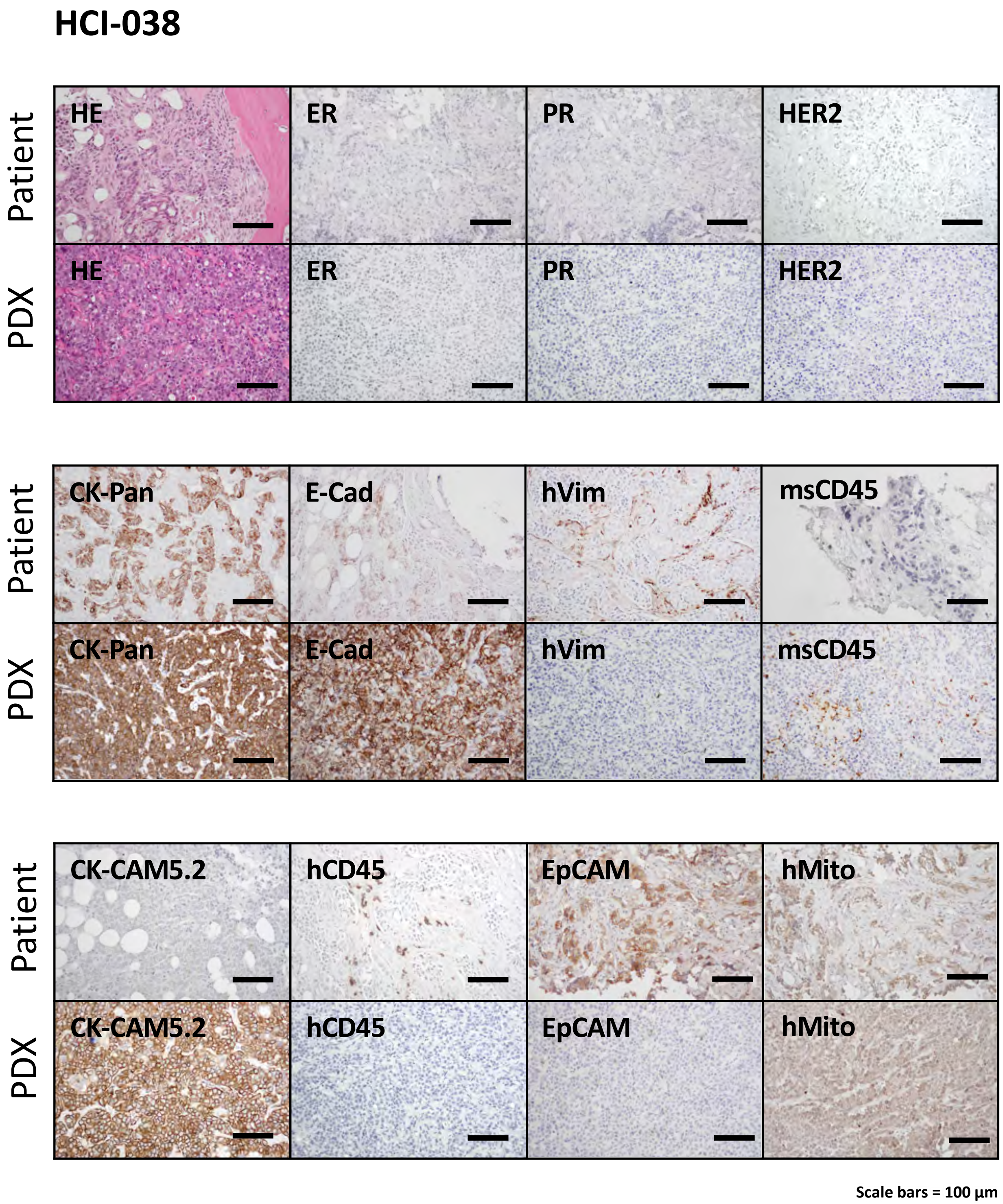

Supplementary Fig. 22

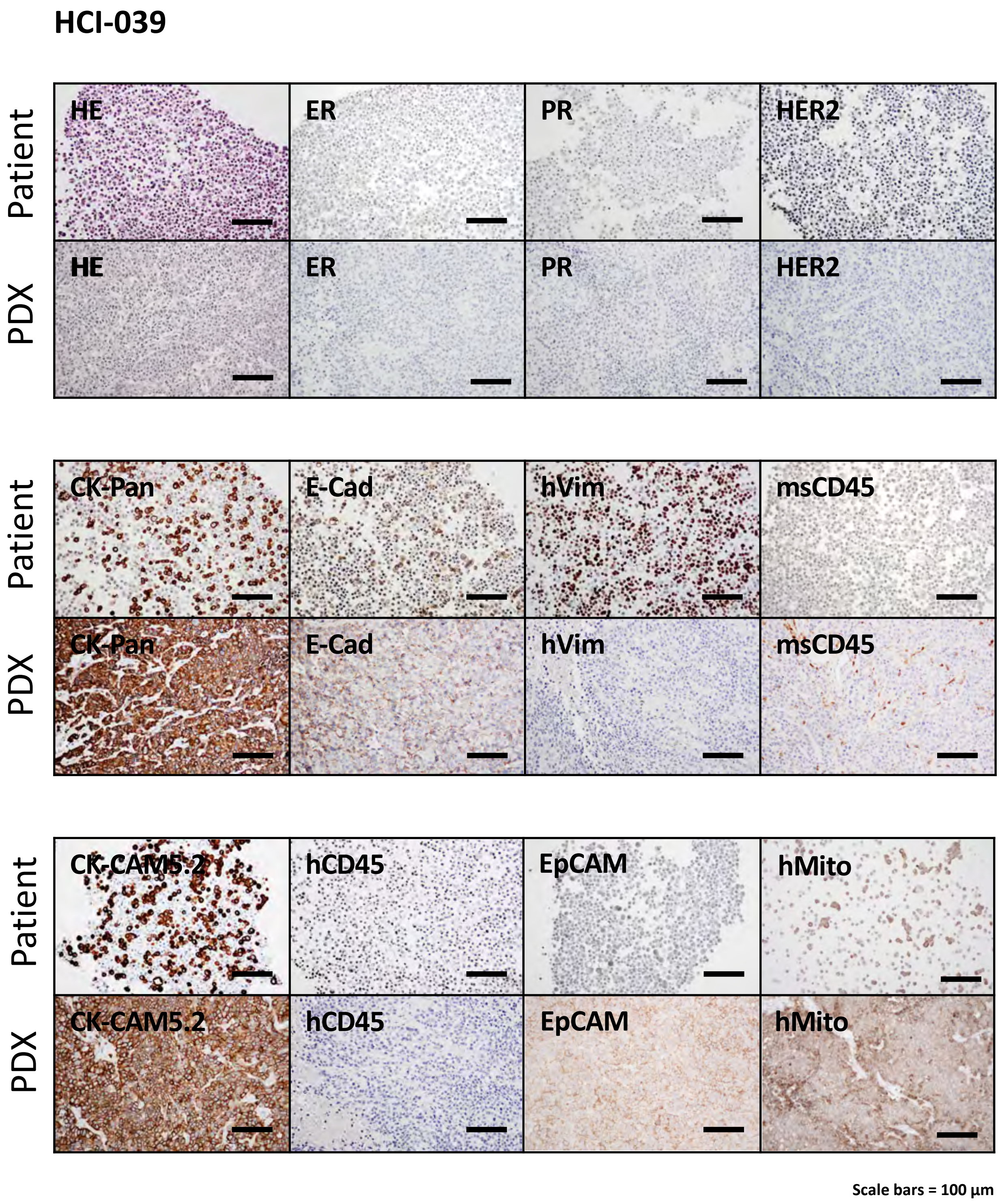

Supplementary Fig. 23

HCI-040

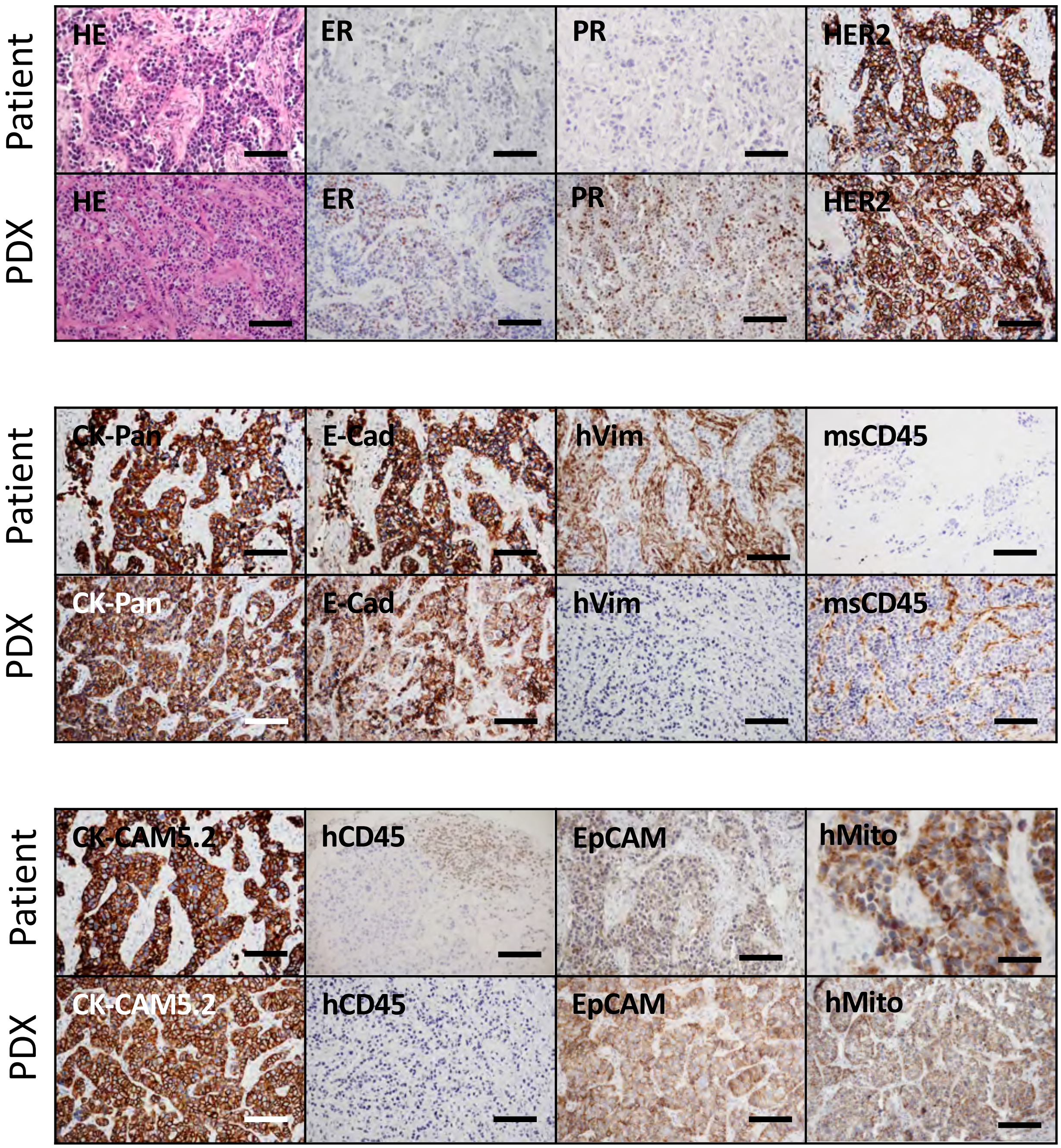

Scale bars = 100 µm

Supplementary Fig. 24

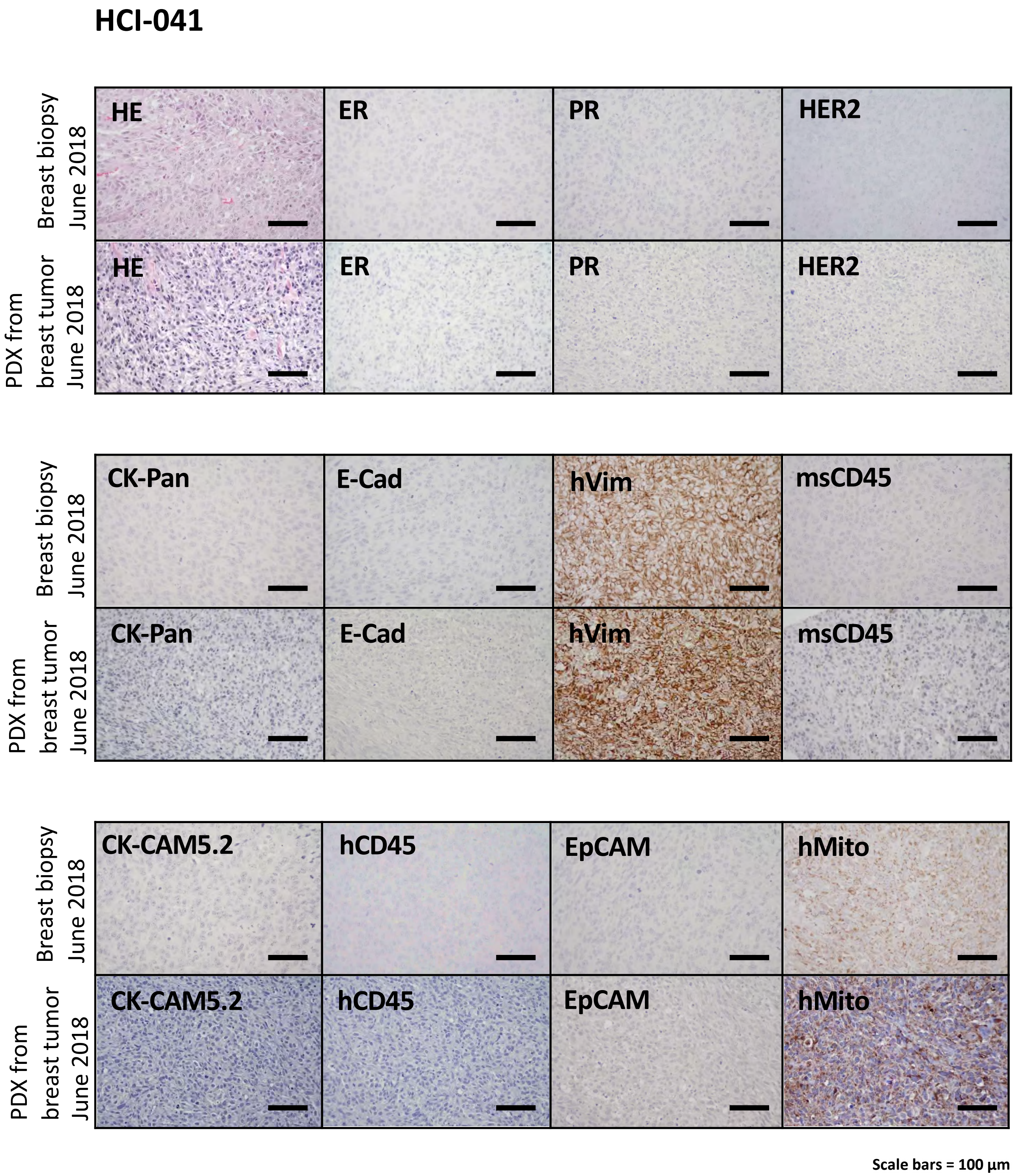

Supplementary Fig. 25

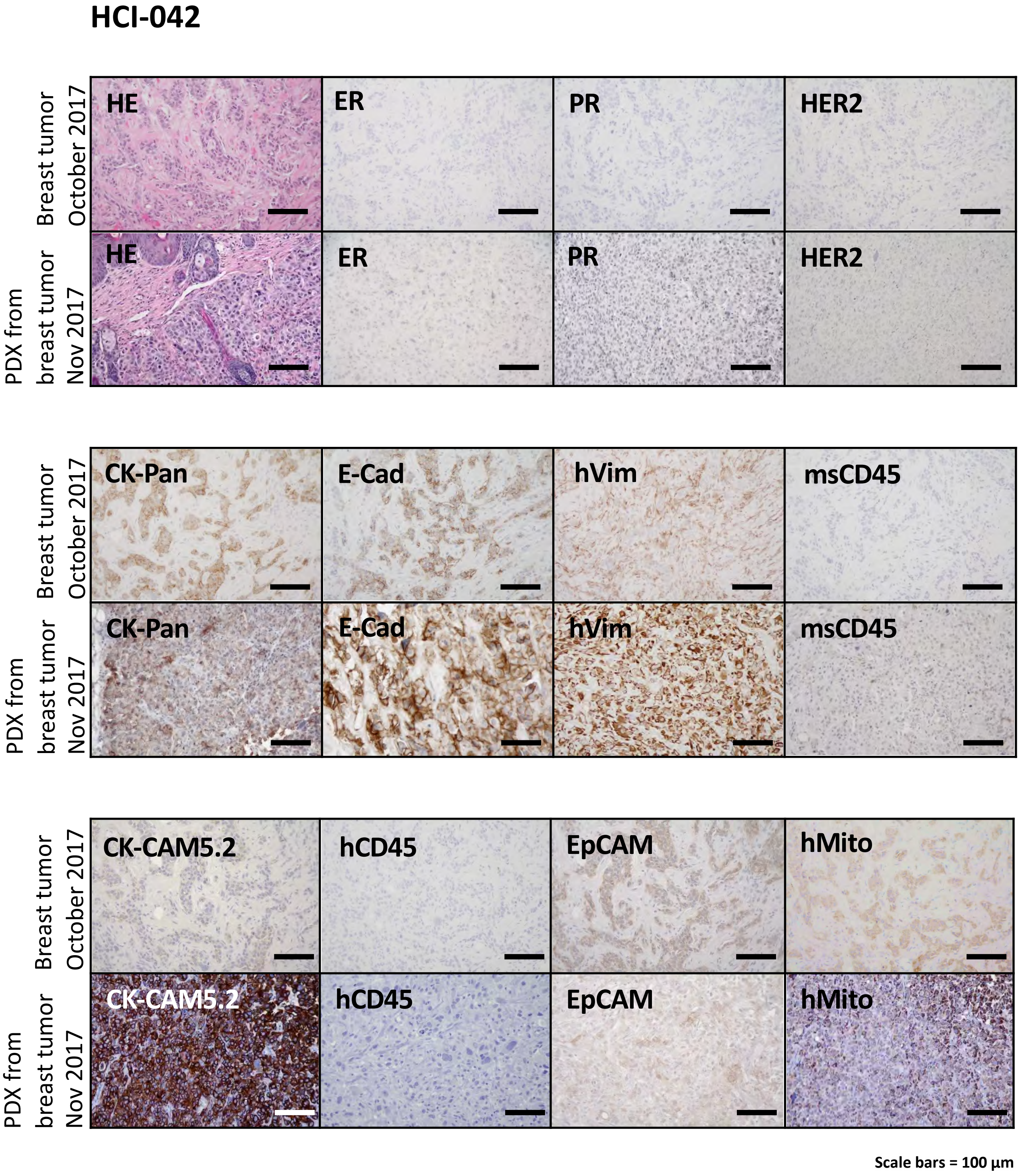

Supplementary Fig. 26

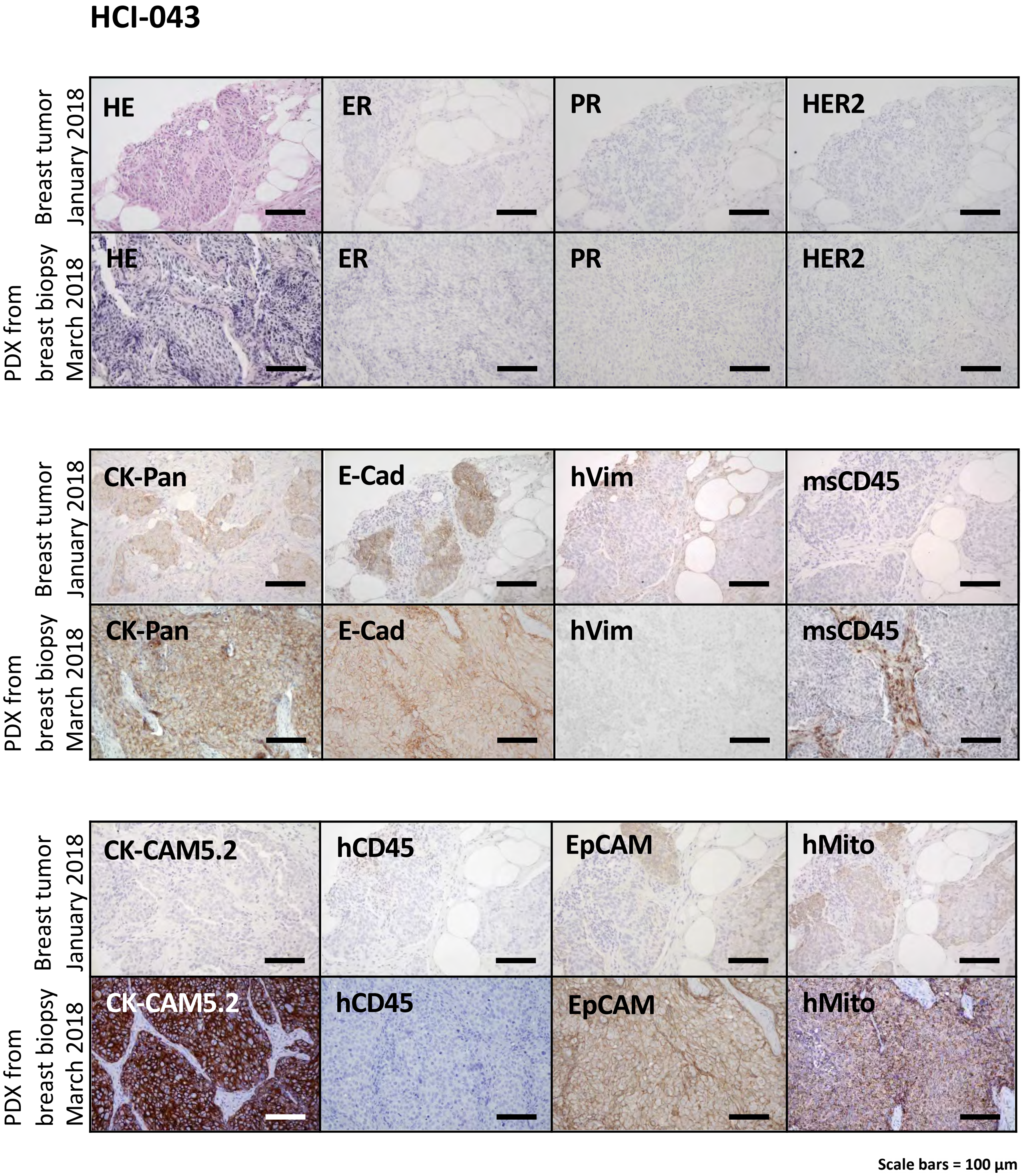

Supplementary Fig. 27

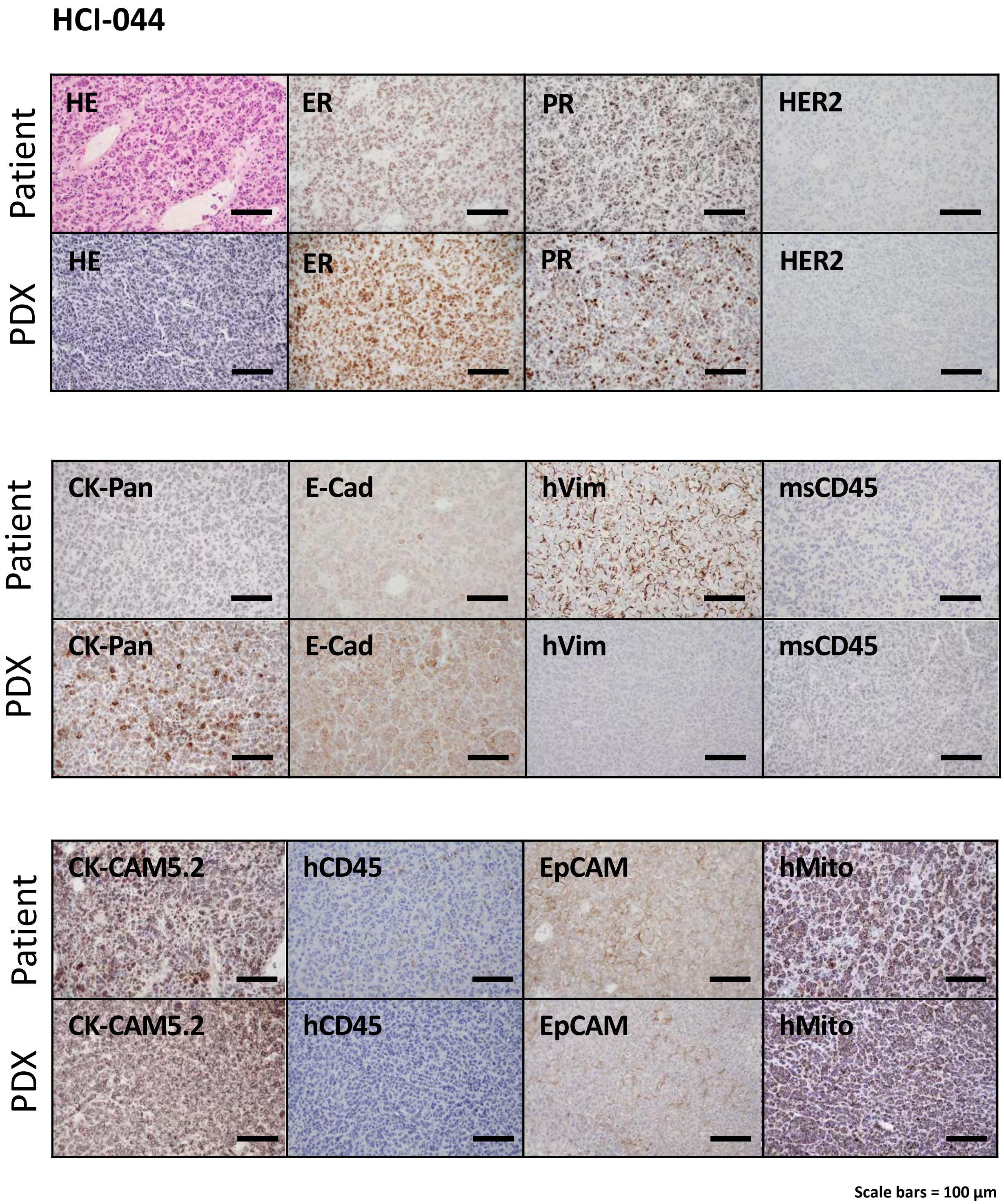

Supplementary Fig. 28

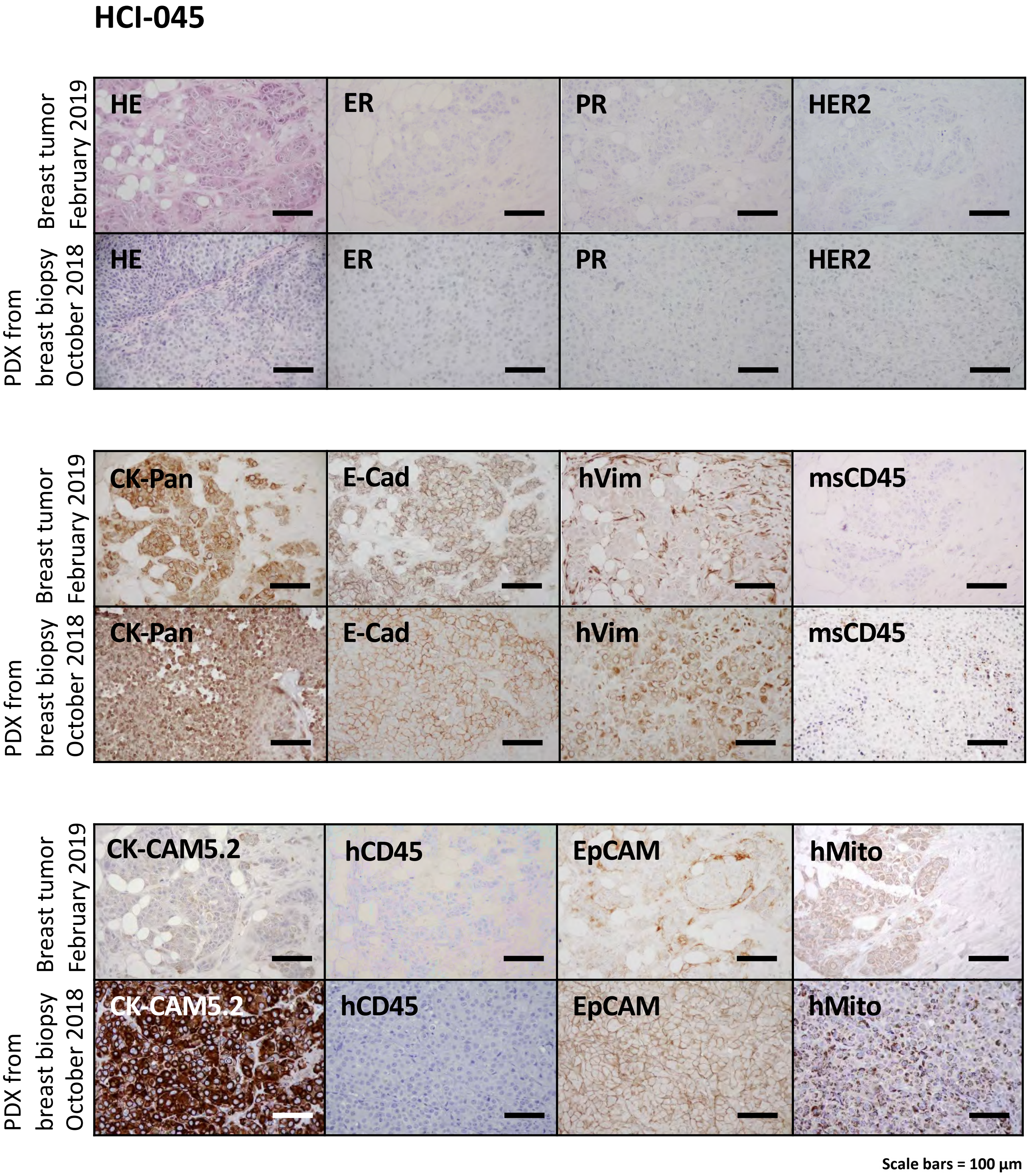

Supplementary Fig. 29

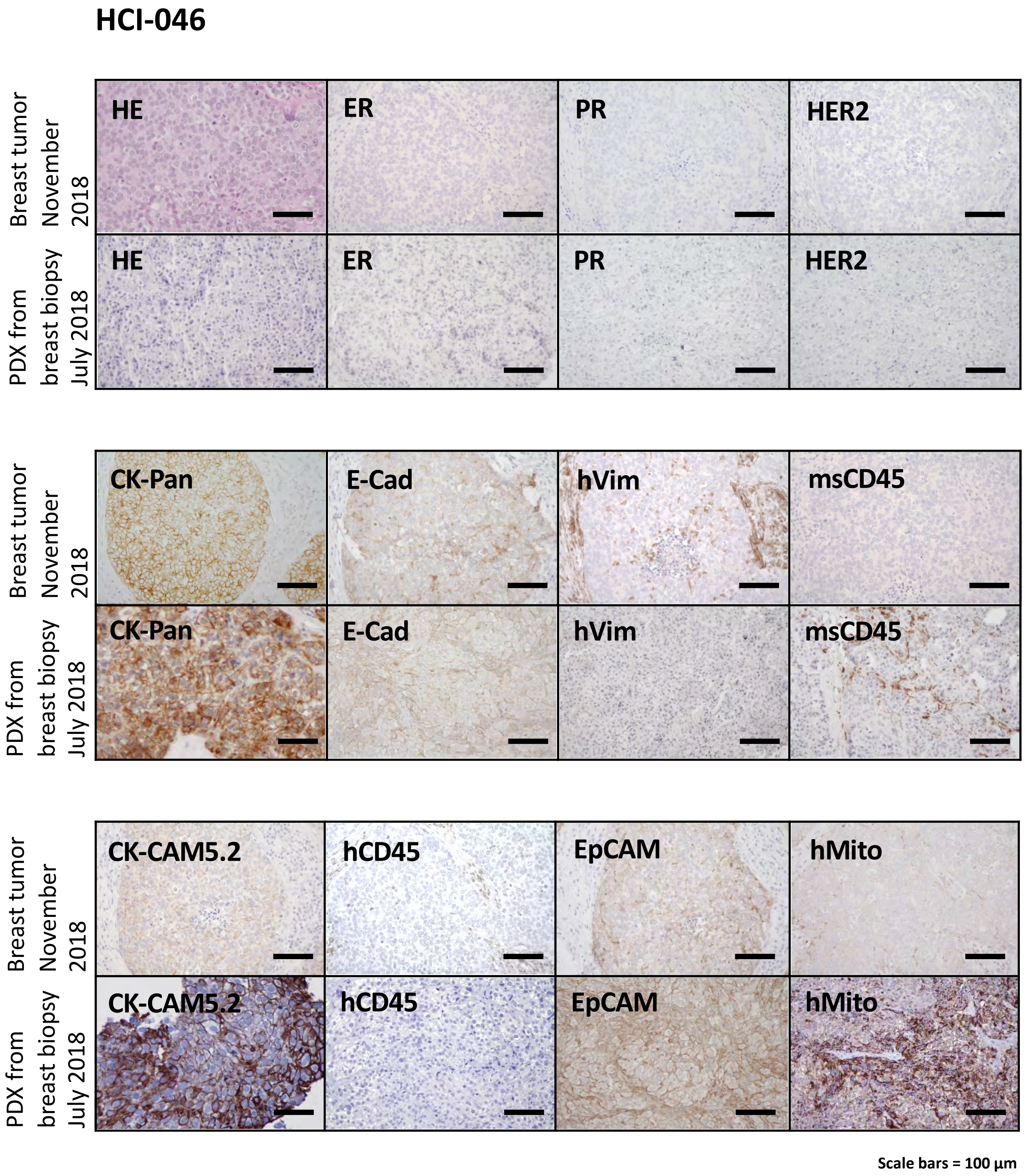

Supplementary Fig. 30

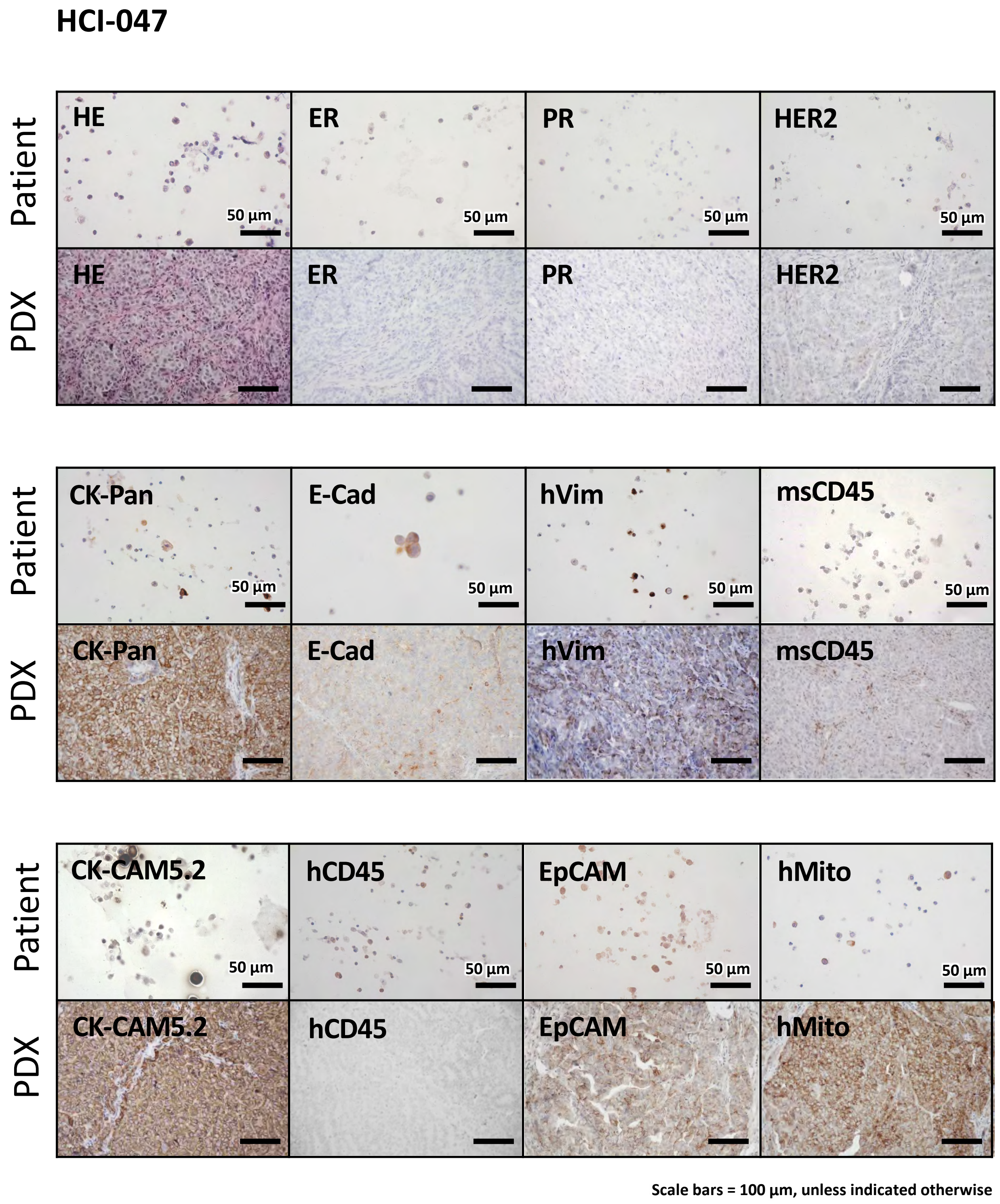

Supplementary Fig. 31

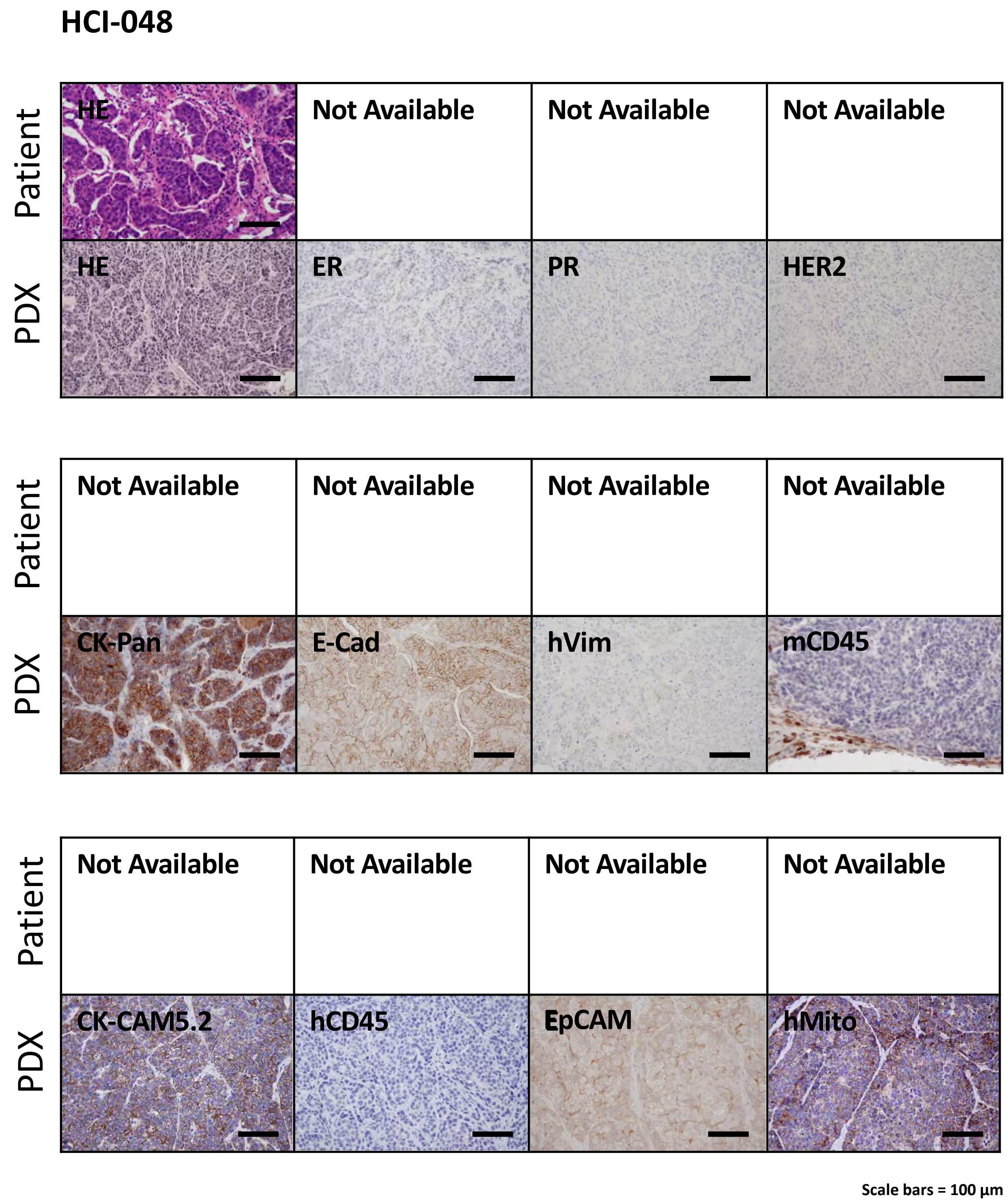

Supplementary Fig. 32

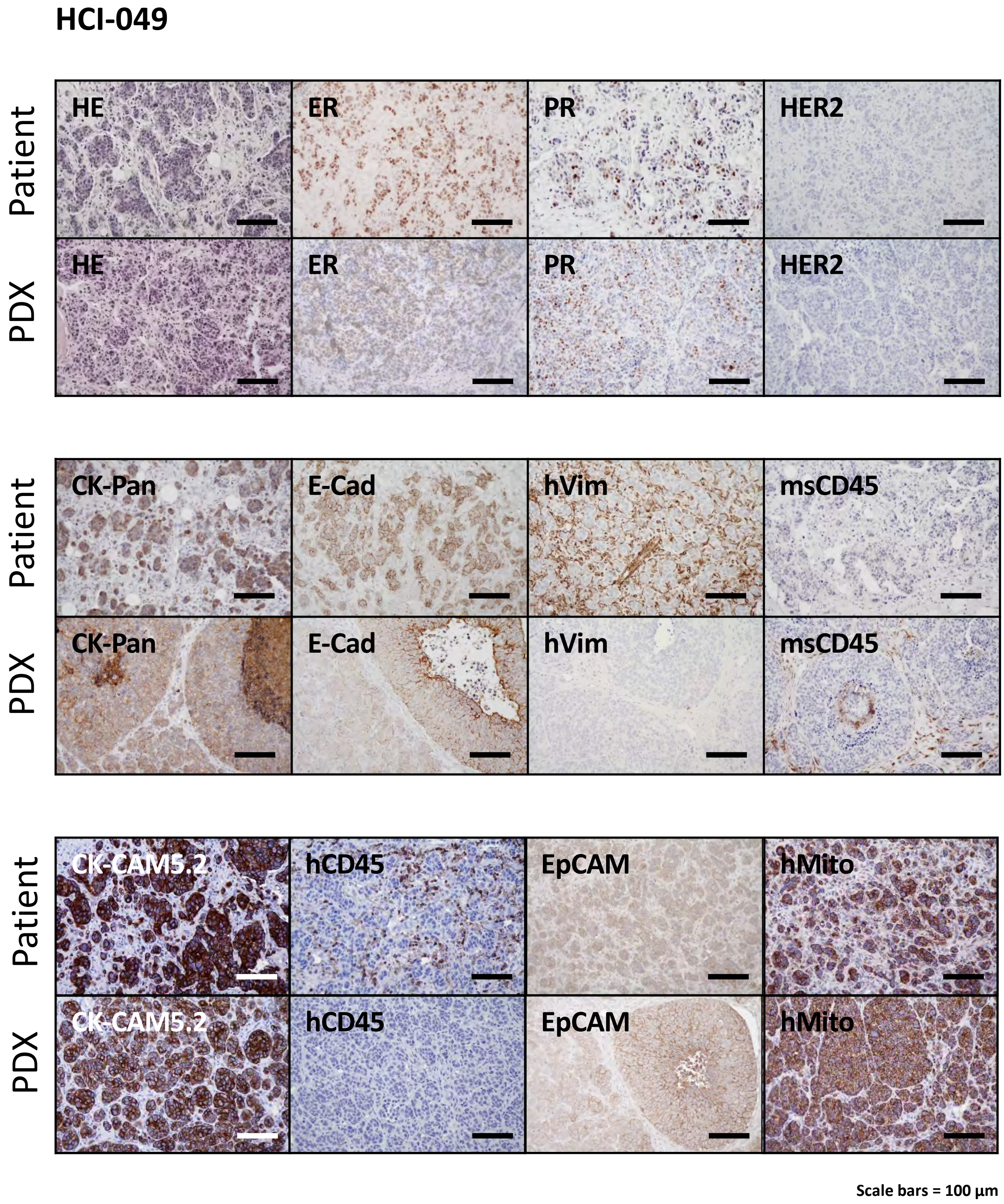

Supplementary Fig. 33

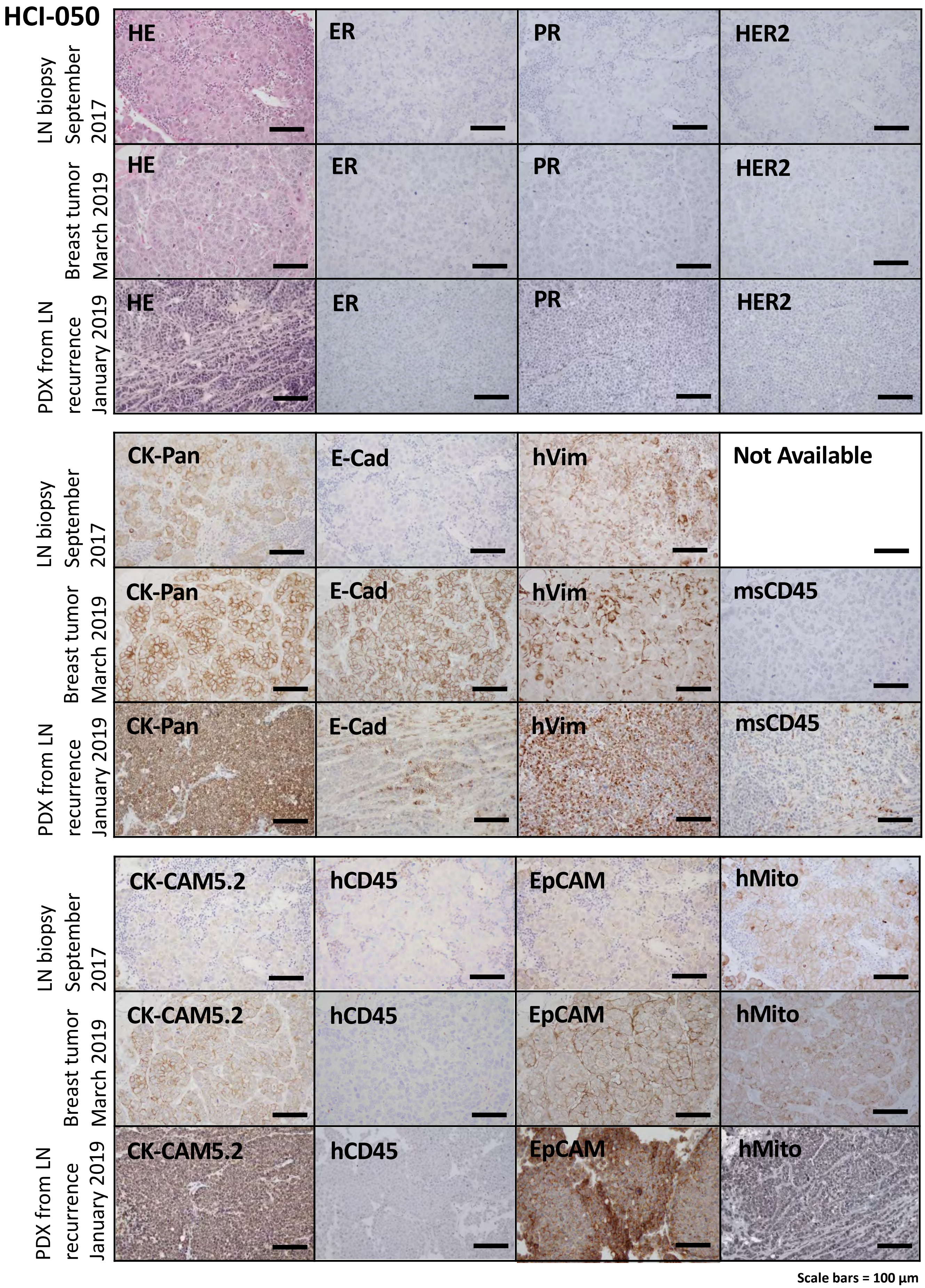

Supplementary Fig. 34

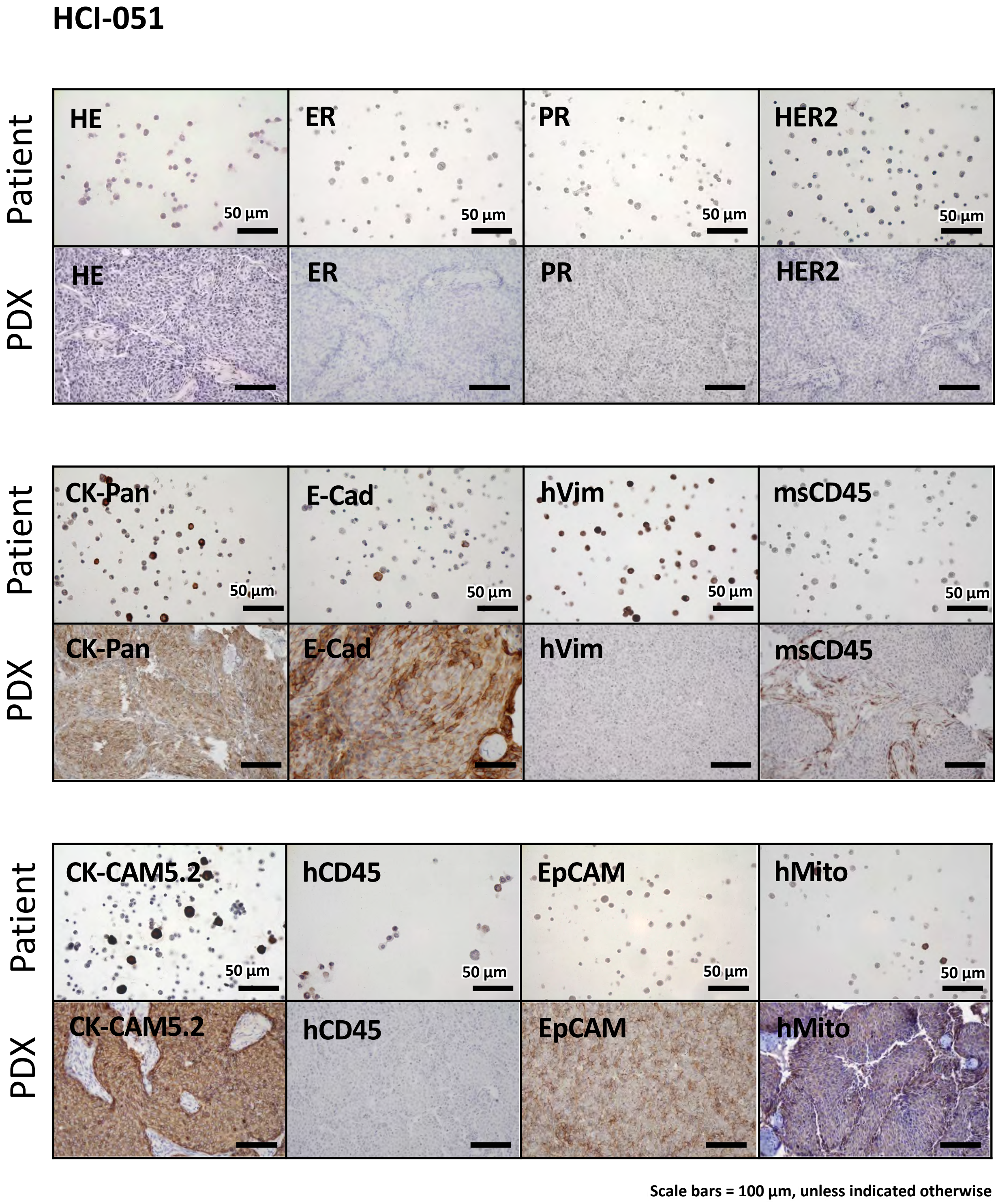

Supplementary Fig. 35

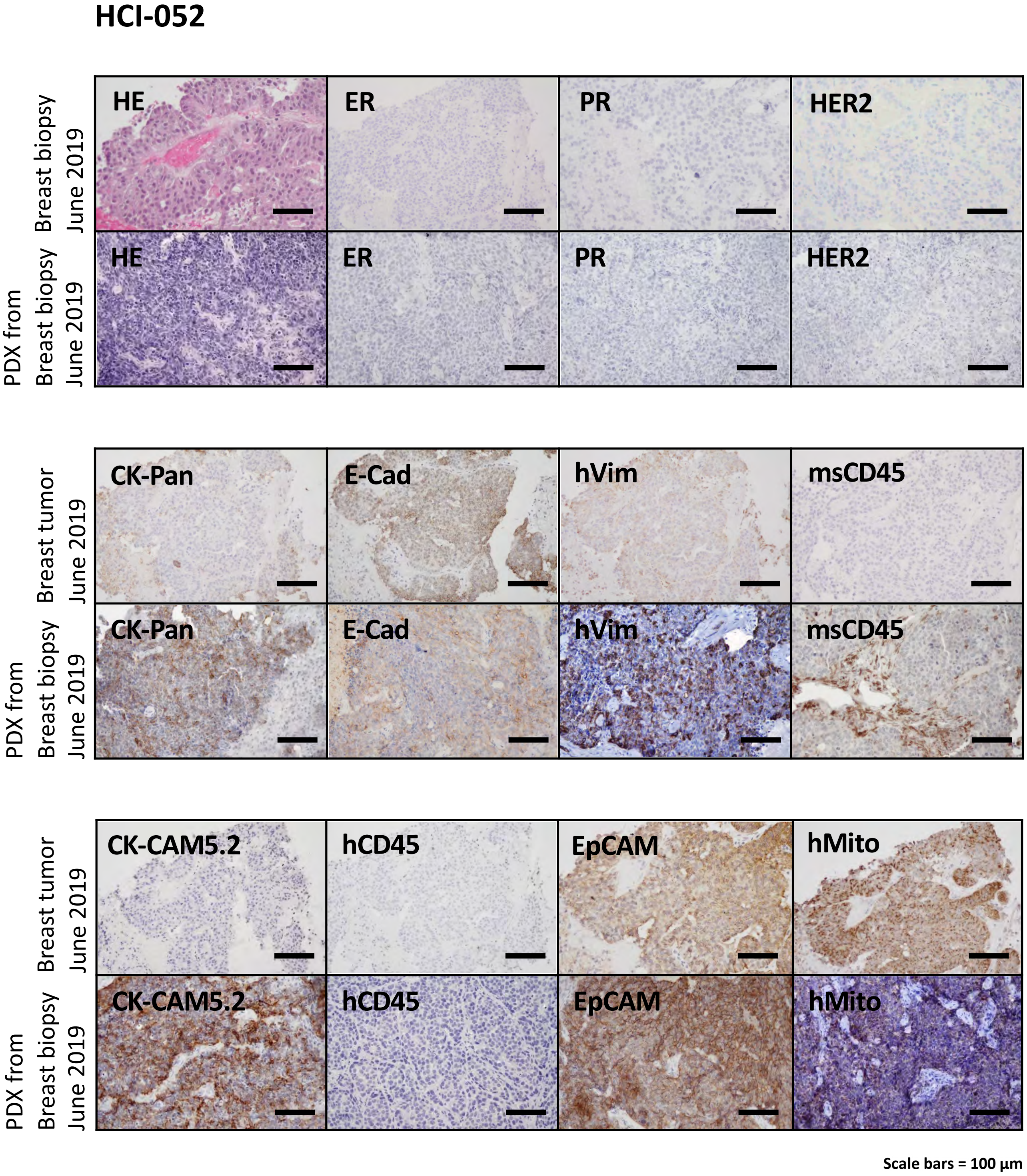

Supplementary Fig. 36

HCI-053

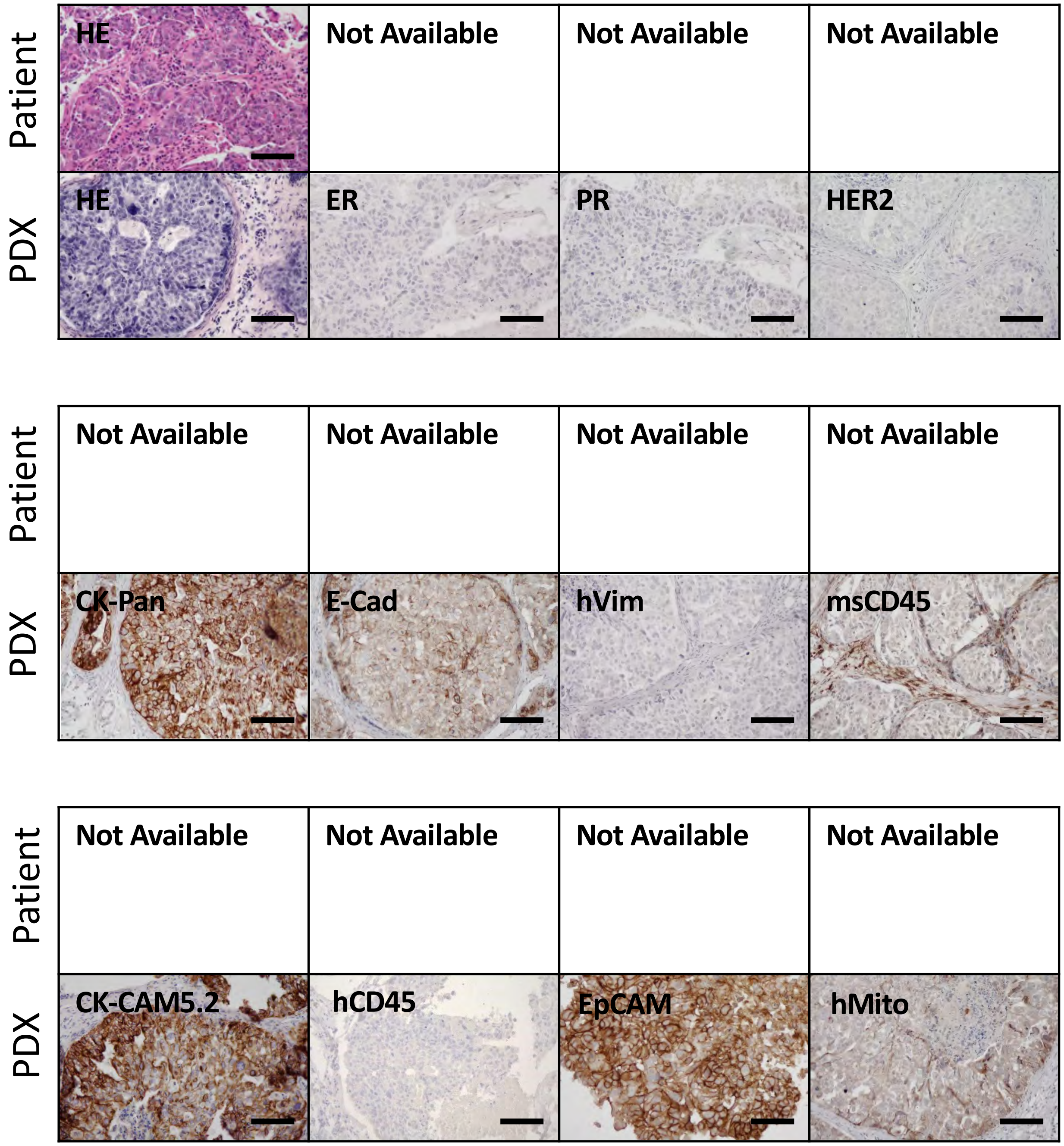

Scale bars = 100 µm

Supplementary Fig. 37

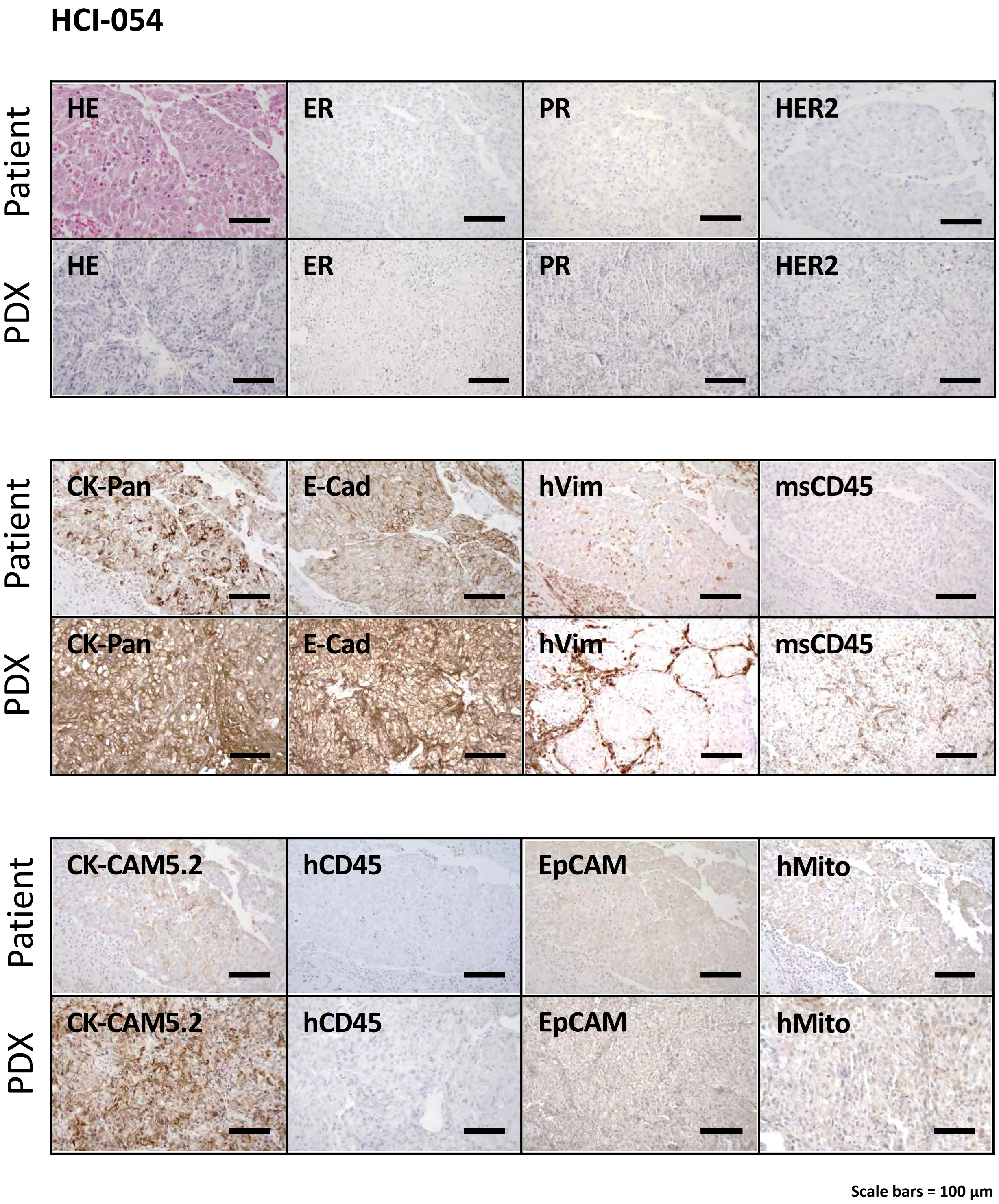

Supplemental Fig. 38

a

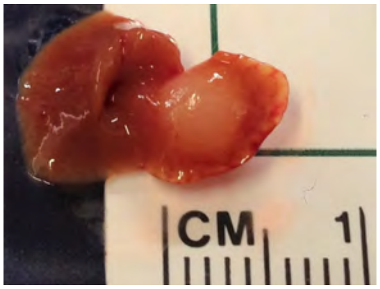

b

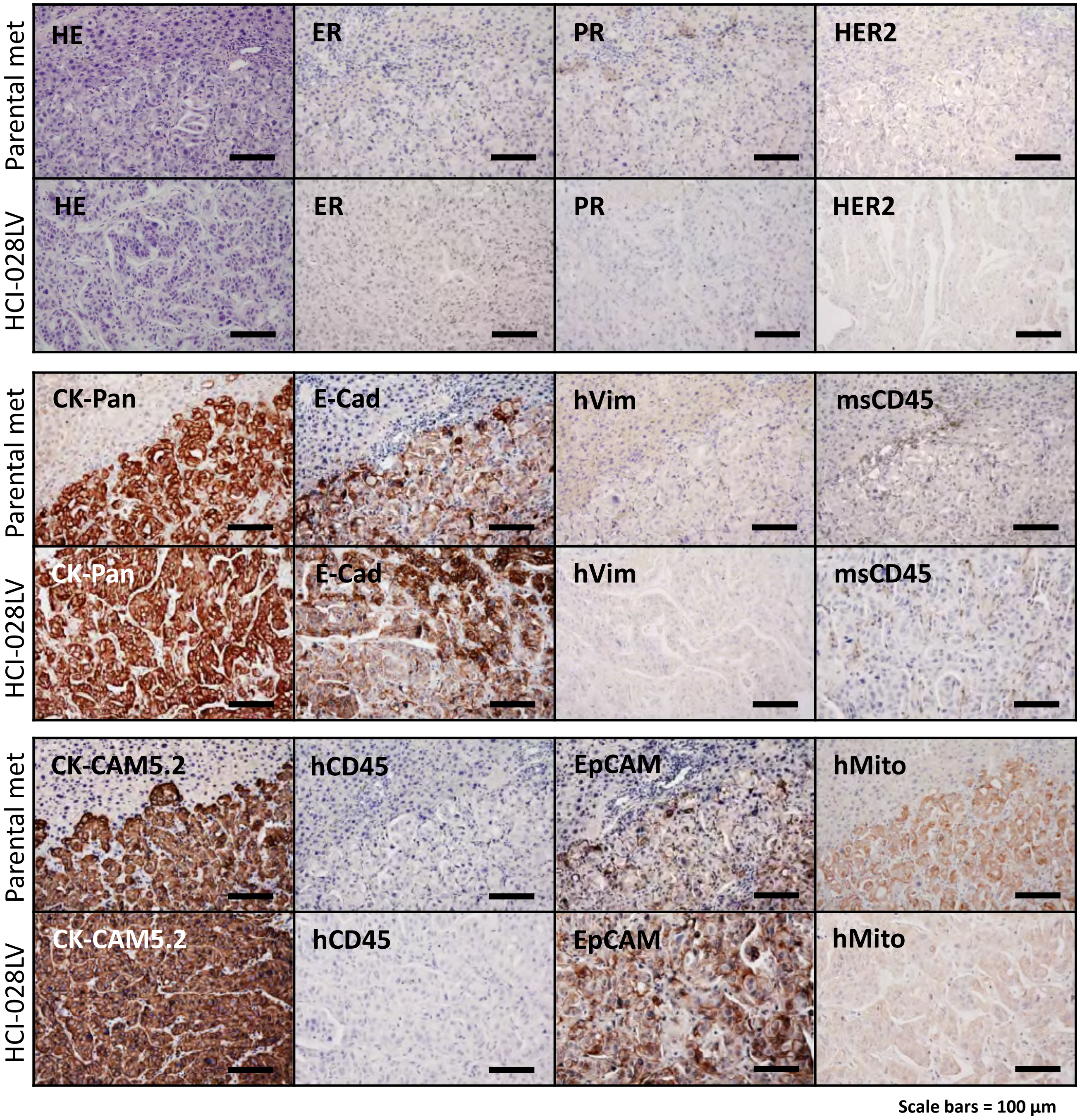

Supplemental Fig. 39

a

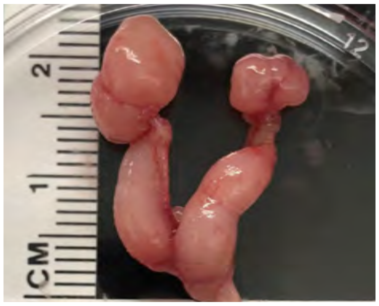

b

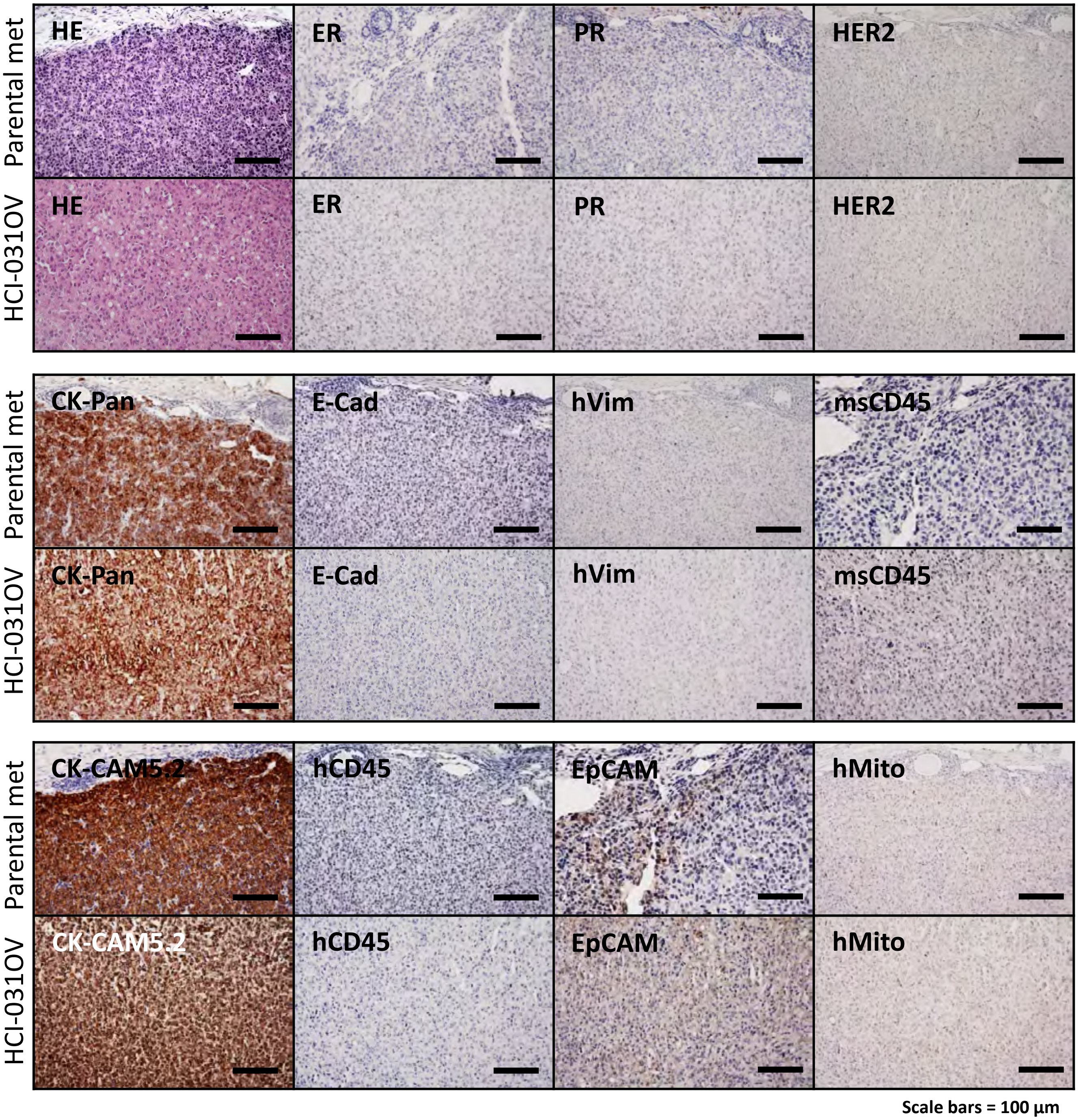

Supplementary Fig. 40

a **HCI-013EI**

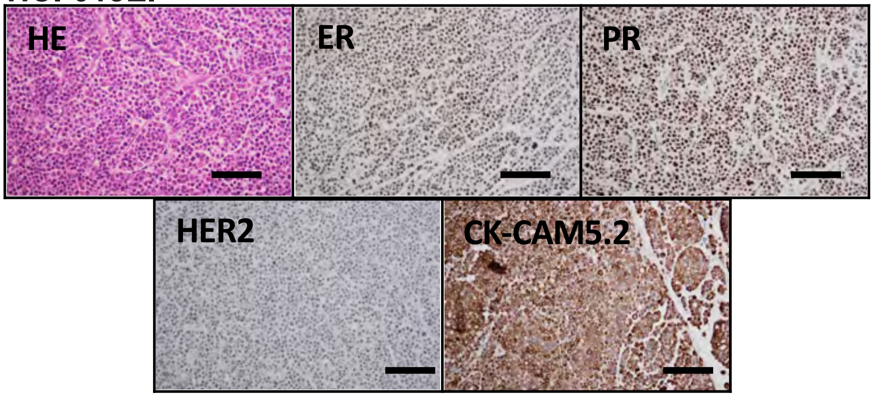

b **HCI-032EI**

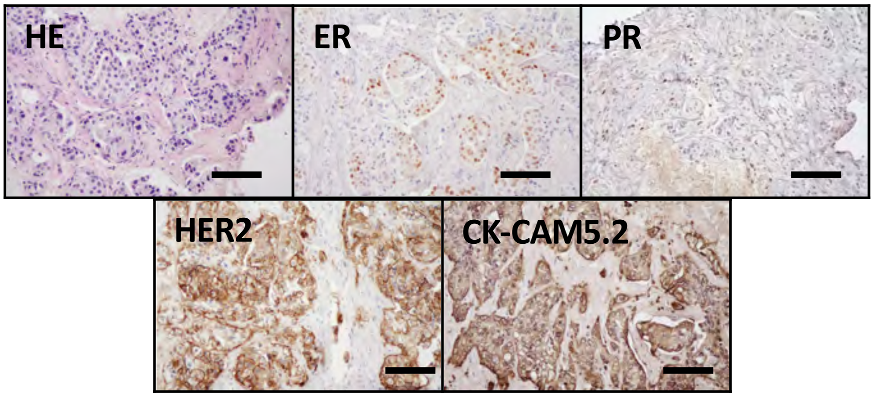

c **HCI-040EI**

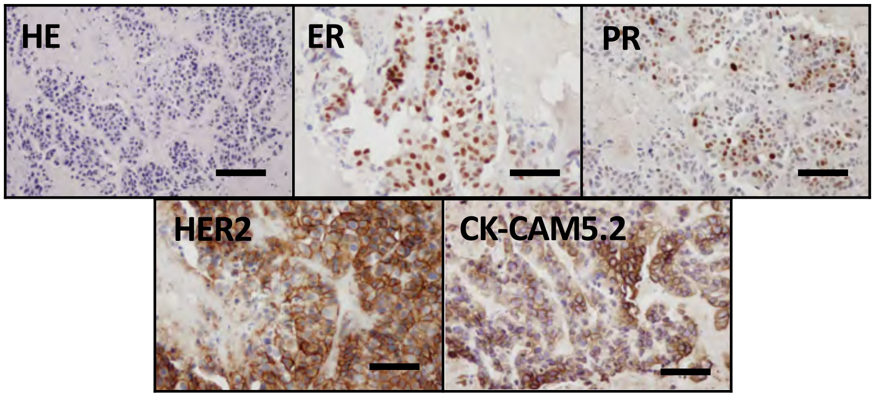

d **HCI-044EI**

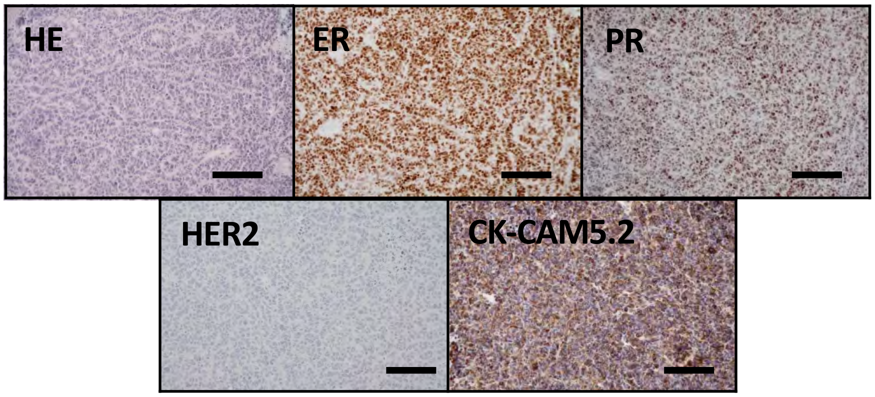

Scale bars = 100 µm

Supplementary Fig. 41

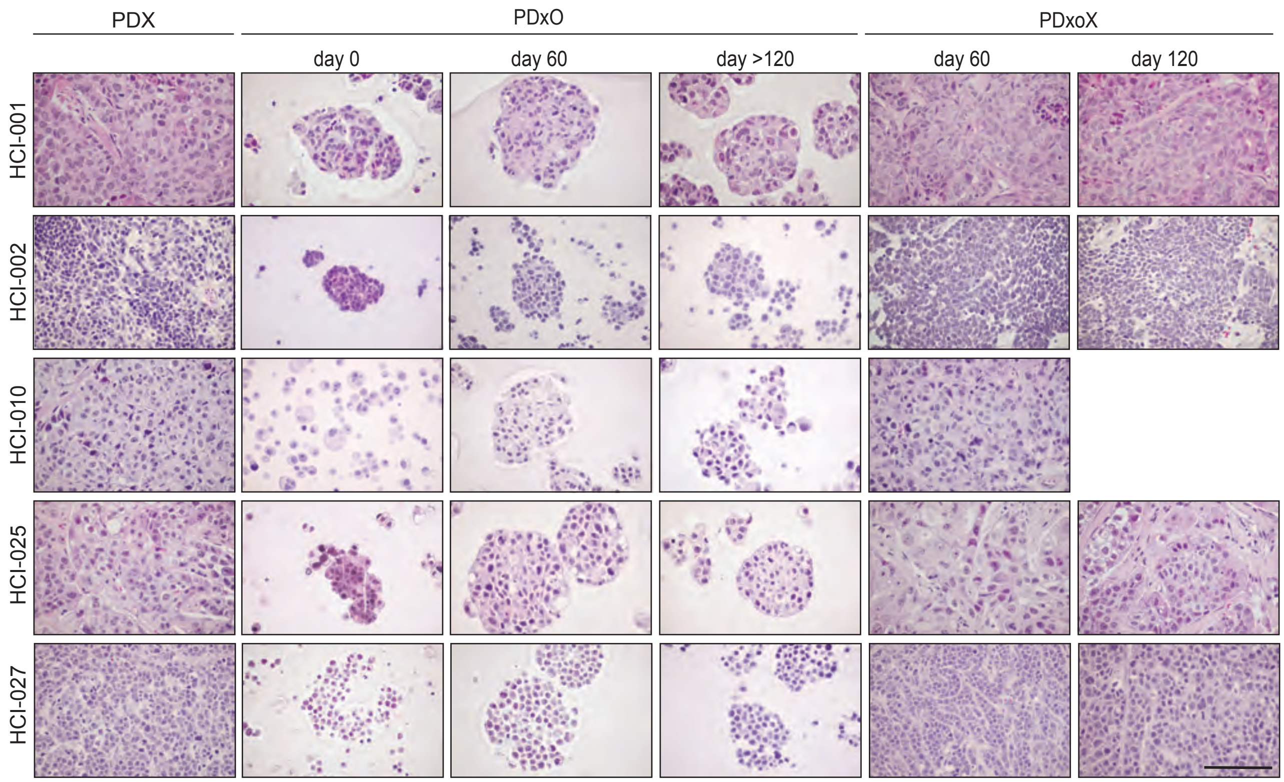

Supplementary Fig. 42

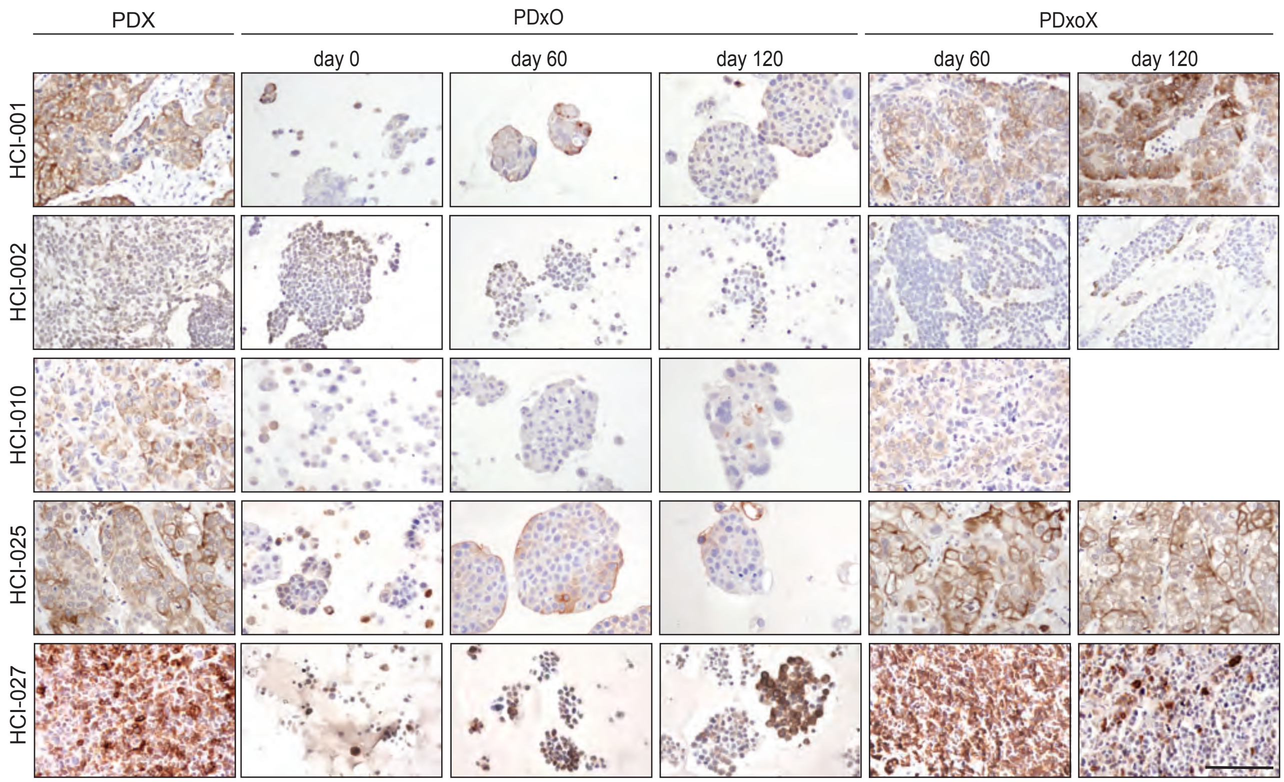

Supplementary Fig. 43

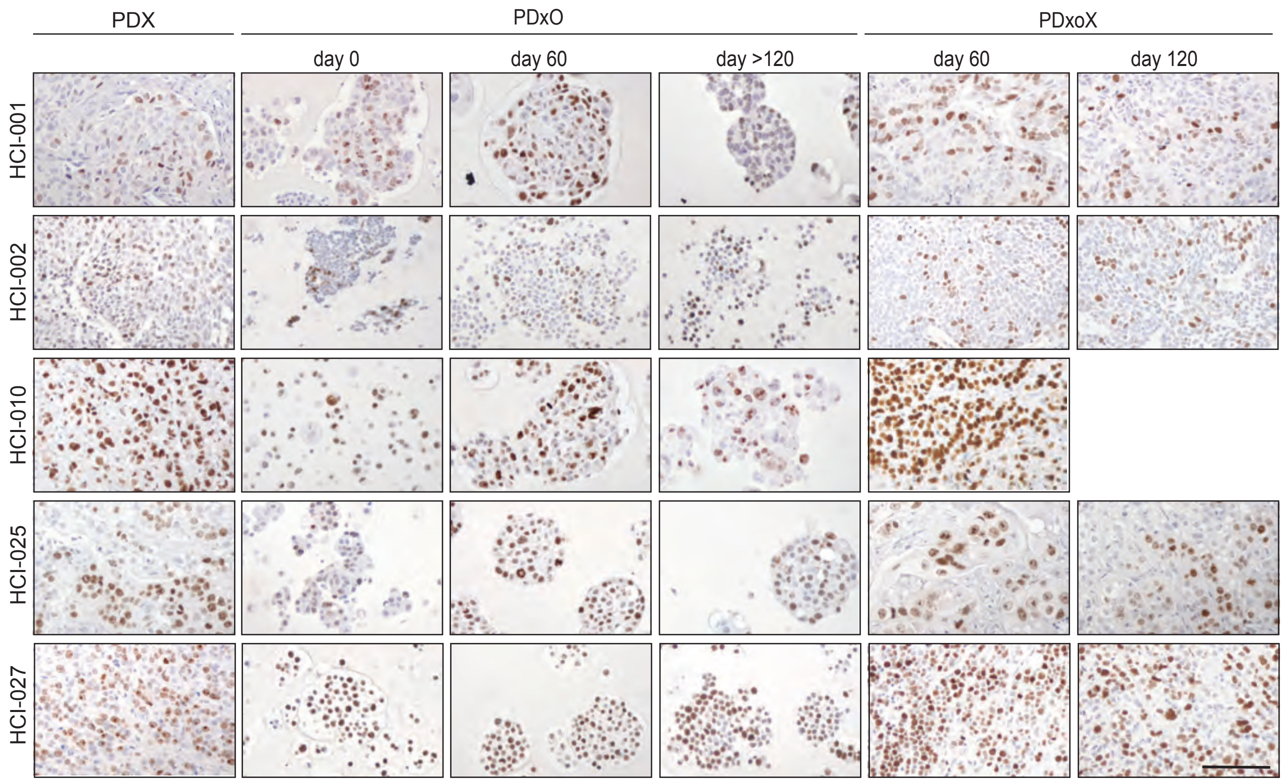

Supplementary Fig. 44

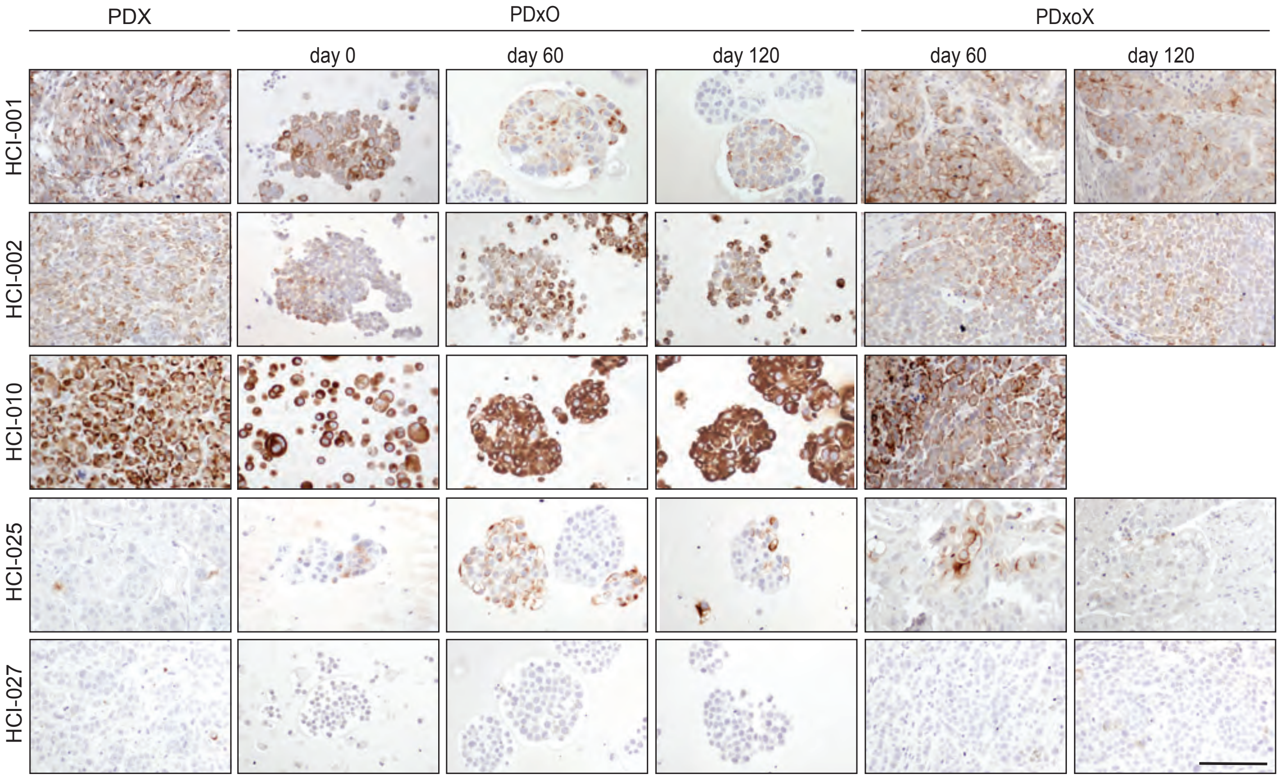

Supplementary Fig. 45

a

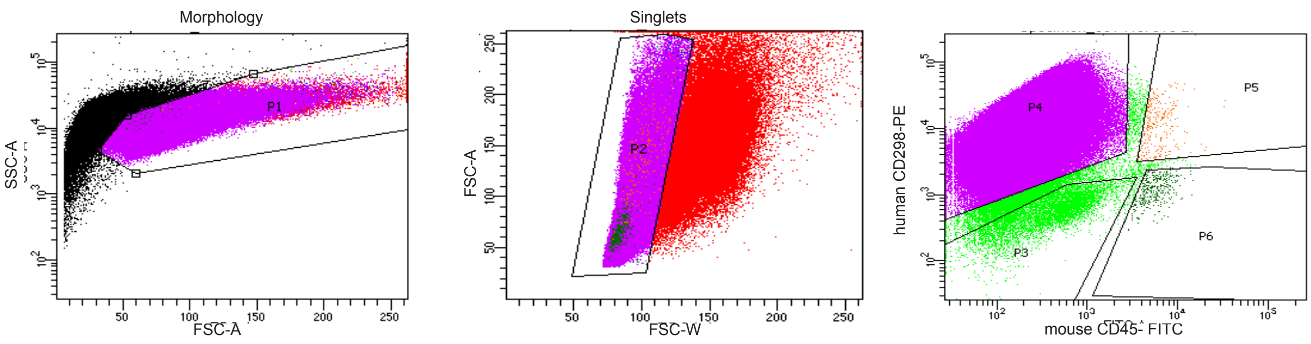

Tube: HCI 013 EI

Population	#Events	%Parent	%Total
All Events	3,259,497	####	100.0
P1	2,549,516	78.2	78.2
P2	2,477,000	97.2	76.0
P3	6,451	0.3	0.2
P4	2,463,148	99.4	75.6
P5	269	0.0	0.0
P6	365	0.0	0.0

b

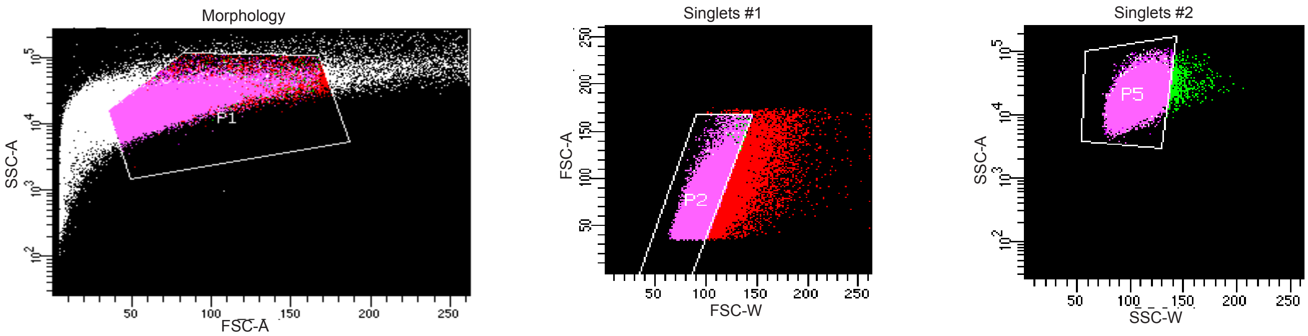

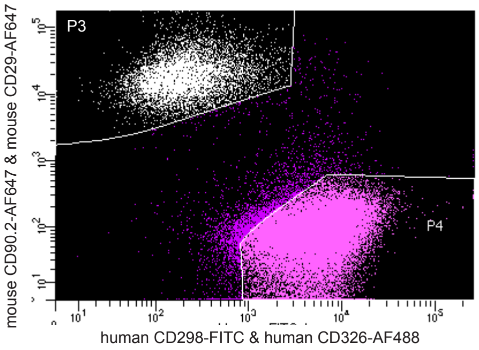

Tube: TOW-14 two mouse and two human ab

Population	#Events	%Parent	%Total
All Events	82,947	####	100.0
P1	57,793	69.7	69.7
P2	49,651	85.9	59.9
P5	49,042	98.8	59.1
P3	6,685	13.6	8.1
P4	39,342	80.2	47.4

Supplemental Figure Legends

Supplemental Figure 1. IHC for HCI-013

Immunohistochemistry of HCI PDX line comparing the original patient tumor (as a cytospin) to the resulting PDX tumor. Stainings: H&E, ER, PR, HER2, CK-pan (Cytokeratin-pan), E-Cad (E-cadherin), hVim (human vimentin), msCD45, CK-CAM5.2 (human specific cytokeratin CAM5.2), hCD45, EpCAM, and hMito (human mitochondria). Each IHC staining has been performed once.

Supplemental Figure 2. IHC for HCI-014

Immunohistochemistry of HCI PDX line comparing the original patient tumor to the resulting PDX tumor. Stainings: H&E, ER, PR, HER2, CK-pan (Cytokeratin-pan), E-Cad (E-cadherin), hVim (human vimentin), msCD45, CK-CAM5.2 (human specific cytokeratin CAM5.2), hCD45, EpCAM, and hMito (human mitochondria). Each IHC staining has been performed once, and there was not enough patient tumor sample available for all IHC stains.

Supplemental Figure 3. IHC for HCI-015

Immunohistochemistry of HCI PDX line comparing the original patient tumor to the resulting PDX tumor. Stainings: H&E, ER, PR, HER2, CK-pan (Cytokeratin-pan), E-Cad (E-cadherin), hVim (human vimentin), msCD45, CK-CAM5.2 (human specific cytokeratin CAM5.2), hCD45, EpCAM, and hMito (human mitochondria). Each IHC staining has been performed once.

Supplemental Figure 4. IHC for HCI-016

Immunohistochemistry of HCI PDX line comparing the original patient tumor to the resulting PDX tumor. Stainings: H&E, ER, PR, HER2, CK-pan (Cytokeratin-pan), E-Cad (E-cadherin), hVim (human vimentin), msCD45, CK-CAM5.2 (human specific cytokeratin CAM5.2), hCD45, EpCAM, and hMito (human mitochondria). Each IHC staining has been performed once.

Supplemental Figure 5. IHC for HCI-017

Immunohistochemistry of HCI PDX line comparing the original patient tumor to the resulting PDX tumor. Stainings: H&E, ER, PR, HER2, CK-pan (Cytokeratin-pan), E-Cad (E-cadherin), hVim (human vimentin), msCD45, CK-CAM5.2 (human specific cytokeratin CAM5.2), hCD45, EpCAM, and hMito (human mitochondria). Each IHC staining has been performed once.

Supplemental Figure 6. IHC for HCI-018

Immunohistochemistry of HCI PDX line comparing the original patient tumor to the resulting PDX tumor. Stainings: H&E, ER, PR, HER2, CK-pan (Cytokeratin-pan), E-Cad (E-cadherin), hVim (human vimentin), msCD45, CK-CAM5.2 (human specific cytokeratin CAM5.2), hCD45, EpCAM, and hMito (human mitochondria). Each IHC staining has been performed once.

Supplemental Figure 7. IHC for HCI-019

Immunohistochemistry of HCI PDX line comparing the original patient tumor to the resulting PDX tumor. Stainings: H&E, ER, PR, HER2, CK-pan (Cytokeratin-pan), E-Cad (E-cadherin), hVim (human vimentin), msCD45, CK-CAM5.2 (human specific cytokeratin CAM5.2), hCD45, EpCAM, and hMito (human mitochondria). Each IHC staining has been performed once.

Supplemental Figure 8. IHC for HCI-023

Immunohistochemistry of HCI PDX line comparing the original patient tumor to the resulting PDX tumor. Stainings: H&E, ER, PR, HER2, CK-pan (Cytokeratin-pan), E-Cad (E-cadherin), hVim (human vimentin), msCD45, CK-CAM5.2 (human specific cytokeratin CAM5.2), hCD45, EpCAM, and hMito (human mitochondria). Each IHC staining has been performed once.

Supplemental Figure 9. IHC for HCI-024

Immunohistochemistry of HCI PDX line comparing the original patient tumor to the resulting PDX tumor. Stainings: H&E, ER, PR, HER2, CK-pan (Cytokeratin-pan), E-Cad (E-cadherin), hVim (human vimentin), msCD45, CK-CAM5.2 (human specific cytokeratin CAM5.2), hCD45, EpCAM, and hMito (human mitochondria). Each IHC staining has been performed once.

Supplemental Figure 10. IHC for HCI-025

Immunohistochemistry of HCI PDX line comparing the original patient tumor to the resulting PDX tumor. Stainings: H&E, ER, PR, HER2, CK-pan (Cytokeratin-pan), E-Cad (E-cadherin), hVim (human vimentin), msCD45, CK-CAM5.2 (human specific cytokeratin CAM5.2), hCD45, EpCAM, and hMito (human mitochondria). Each IHC staining has been performed once.

Supplemental Figure 11. IHC for HCI-026

Immunohistochemistry of HCI PDX line comparing the original patient tumor to the resulting PDX tumor. Stainings: H&E, ER, PR, HER2, CK-pan (Cytokeratin-pan), E-Cad (E-cadherin), hVim (human vimentin),

msCD45, CK-CAM5.2 (human specific cytokeratin CAM5.2), hCD45, EpCAM, and hMito (human mitochondria). Each IHC staining has been performed once.

Supplemental Figure 12. IHC for HCI-027

Immunohistochemistry of HCI PDX line comparing the original patient tumor to the resulting PDX tumor. Stainings: H&E, ER, PR, HER2, CK-pan (Cytokeratin-pan), E-Cad (E-cadherin), hVim (human vimentin), msCD45, CK-CAM5.2 (human specific cytokeratin CAM5.2), hCD45, EpCAM, and hMito (human mitochondria). Each IHC staining has been performed once.

Supplemental Figure 13. IHC for HCI-028

Immunohistochemistry of HCI PDX line comparing the original patient tumor to the resulting PDX tumor. Stainings: H&E, ER, PR, HER2, CK-pan (Cytokeratin-pan), E-Cad (E-cadherin), hVim (human vimentin), msCD45, CK-CAM5.2 (human specific cytokeratin CAM5.2), hCD45, EpCAM, and hMito (human mitochondria). Each IHC staining has been performed once.

Supplemental Figure 14. IHC for HCI-030

Immunohistochemistry of HCI PDX line comparing the original patient tumor to the resulting PDX tumor. Stainings: H&E, ER, PR, HER2, CK-pan (Cytokeratin-pan), E-Cad (E-cadherin), hVim (human vimentin), msCD45, CK-CAM5.2 (human specific cytokeratin CAM5.2), hCD45, EpCAM, and hMito (human mitochondria). Each IHC staining has been performed once.

Supplemental Figure 15. IHC for HCI-031

Immunohistochemistry of HCI PDX line comparing the original patient tumor (as a cytospin) to the resulting PDX tumor. Stainings: H&E, ER, PR, HER2, CK-pan (Cytokeratin-pan), E-Cad (E-cadherin), hVim (human vimentin), msCD45, CK-CAM5.2 (human specific cytokeratin CAM5.2), hCD45, EpCAM, and hMito (human mitochondria). Each IHC staining has been performed once.

Supplemental Figure 16. IHC for HCI-032

Immunohistochemistry of HCI PDX line comparing the original patient tumor to the resulting PDX tumor. Stainings: H&E, ER, PR, HER2, CK-pan (Cytokeratin-pan), E-Cad (E-cadherin), hVim (human vimentin), msCD45, CK-CAM5.2 (human specific cytokeratin CAM5.2), hCD45, EpCAM, and hMito (human mitochondria). Each IHC staining has been performed once.

Supplemental Figure 17. IHC for HCI-033

Immunohistochemistry of HCI PDX line comparing the original patient tumor to the resulting PDX tumor. Stainings: H&E, ER, PR, HER2, CK-pan (Cytokeratin-pan), E-Cad (E-cadherin), hVim (human vimentin), msCD45, CK-CAM5.2 (human specific cytokeratin CAM5.2), hCD45, EpCAM, and hMito (human mitochondria). Each IHC staining has been performed once.

Supplemental Figure 18. IHC for HCI-034

Immunohistochemistry of HCI PDX line comparing the original patient tumor to the resulting PDX tumor. Stainings: H&E, ER, PR, HER2, CK-pan (Cytokeratin-pan), E-Cad (E-cadherin), hVim (human vimentin), msCD45, CK-CAM5.2 (human specific cytokeratin CAM5.2), hCD45, EpCAM, and hMito (human mitochondria). Each IHC staining has been performed once.

Supplemental Figure 19. IHC for HCI-036

Immunohistochemistry of HCI PDX line comparing the original patient tumor to the resulting PDX tumor. Stainings: H&E, ER, PR, HER2, CK-pan (Cytokeratin-pan), E-Cad (E-cadherin), hVim (human vimentin), msCD45, CK-CAM5.2 (human specific cytokeratin CAM5.2), hCD45, EpCAM, and hMito (human mitochondria). Each IHC staining has been performed once.

Supplemental Figure 20. IHC for HCI-037

Immunohistochemistry of HCI PDX line comparing the original patient tumor to the resulting PDX tumor. Stainings: H&E, ER, PR, HER2, CK-pan (Cytokeratin-pan), E-Cad (E-cadherin), hVim (human vimentin), msCD45, CK-CAM5.2 (human specific cytokeratin CAM5.2), hCD45, EpCAM, and hMito (human mitochondria). Each IHC staining has been performed once.

Supplemental Figure 21. IHC for HCI-038

Immunohistochemistry of HCI PDX line comparing the original patient tumor to the resulting PDX tumor. Stainings: H&E, ER, PR, HER2, CK-pan (Cytokeratin-pan), E-Cad (E-cadherin), hVim (human vimentin), msCD45, CK-CAM5.2 (human specific cytokeratin CAM5.2), hCD45, EpCAM, and hMito (human mitochondria). Each IHC staining has been performed once.

Supplemental Figure 22. IHC for HCI-039

Immunohistochemistry of HCI PDX line comparing the original patient tumor to the resulting PDX tumor. Stainings: H&E, ER, PR, HER2, CK-pan (Cytokeratin-pan), E-Cad (E-cadherin), hVim (human vimentin),

msCD45, CK-CAM5.2 (human specific cytokeratin CAM5.2), hCD45, EpCAM, and hMito (human mitochondria). Each IHC staining has been performed once.

Supplemental Figure 23. IHC for HCI-040

Immunohistochemistry of HCI PDX line comparing the original patient tumor to the resulting PDX tumor. Stainings: H&E, ER, PR, HER2, CK-pan (Cytokeratin-pan), E-Cad (E-cadherin), hVim (human vimentin), msCD45, CK-CAM5.2 (human specific cytokeratin CAM5.2), hCD45, EpCAM, and hMito (human mitochondria). Each IHC staining has been performed once.

Supplemental Figure 24. IHC for HCI-041

Immunohistochemistry of HCI PDX line comparing the original patient tumor to the resulting PDX tumor. Stainings: H&E, ER, PR, HER2, CK-pan (Cytokeratin-pan), E-Cad (E-cadherin), hVim (human vimentin), msCD45, CK-CAM5.2 (human specific cytokeratin CAM5.2), hCD45, EpCAM, and hMito (human mitochondria). Each IHC staining has been performed once.

Supplemental Figure 25. IHC for HCI-042

Immunohistochemistry of HCI PDX line comparing the original patient tumor to the resulting PDX tumor. Stainings: H&E, ER, PR, HER2, CK-pan (Cytokeratin-pan), E-Cad (E-cadherin), hVim (human vimentin), msCD45, CK-CAM5.2 (human specific cytokeratin CAM5.2), hCD45, EpCAM, and hMito (human mitochondria). Each IHC staining has been performed once.

Supplemental Figure 26. IHC for HCI-043

Immunohistochemistry of HCI PDX line comparing the original patient tumor to the resulting PDX tumor. Stainings: H&E, ER, PR, HER2, CK-pan (Cytokeratin-pan), E-Cad (E-cadherin), hVim (human vimentin), msCD45, CK-CAM5.2 (human specific cytokeratin CAM5.2), hCD45, EpCAM, and hMito (human mitochondria). Each IHC staining has been performed once.

Supplemental Figure 27. IHC for HCI-044

Immunohistochemistry of HCI PDX line comparing the original patient tumor to the resulting PDX tumor. Stainings: H&E, ER, PR, HER2, CK-pan (Cytokeratin-pan), E-Cad (E-cadherin), hVim (human vimentin), msCD45, CK-CAM5.2 (human specific cytokeratin CAM5.2), hCD45, EpCAM, and hMito (human mitochondria). Each IHC staining has been performed once.

Supplemental Figure 28. IHC for HCI-045

Immunohistochemistry of HCI PDX line comparing the original patient tumor to the resulting PDX tumor. Stainings: H&E, ER, PR, HER2, CK-pan (Cytokeratin-pan), E-Cad (E-cadherin), hVim (human vimentin), msCD45, CK-CAM5.2 (human specific cytokeratin CAM5.2), hCD45, EpCAM, and hMito (human mitochondria). Each IHC staining has been performed once.

Supplemental Figure 29. IHC for HCI-046

Immunohistochemistry of HCI PDX line comparing the original patient tumor to the resulting PDX tumor. Stainings: H&E, ER, PR, HER2, CK-pan (Cytokeratin-pan), E-Cad (E-cadherin), hVim (human vimentin), msCD45, CK-CAM5.2 (human specific cytokeratin CAM5.2), hCD45, EpCAM, and hMito (human mitochondria). Each IHC staining has been performed once.

Supplemental Figure 30. IHC for HCI-047

Immunohistochemistry of HCI PDX line comparing the original patient tumor (as a cytospin) to the resulting PDX tumor. Stainings: H&E, ER, PR, HER2, CK-pan (Cytokeratin-pan), E-Cad (E-cadherin), hVim (human vimentin), msCD45, CK-CAM5.2 (human specific cytokeratin CAM5.2), hCD45, EpCAM, and hMito (human mitochondria). Each IHC staining has been performed once.

Supplemental Figure 31. IHC for HCI-048

Immunohistochemistry of HCI PDX line comparing the original patient tumor to the resulting PDX tumor. Stainings: H&E, ER, PR, HER2, CK-pan (Cytokeratin-pan), E-Cad (E-cadherin), hVim (human vimentin), msCD45, CK-CAM5.2 (human specific cytokeratin CAM5.2), hCD45, EpCAM, and hMito (human mitochondria). Each IHC staining has been performed once. There was not enough patient tumor sample available for IHC stains.

Supplemental Figure 32. IHC for HCI-049

Immunohistochemistry of HCI PDX line comparing the original patient tumor to the resulting PDX tumor. Stainings: H&E, ER, PR, HER2, CK-pan (Cytokeratin-pan), E-Cad (E-cadherin), hVim (human vimentin), msCD45, CK-CAM5.2 (human specific cytokeratin CAM5.2), hCD45, EpCAM, and hMito (human mitochondria). Each IHC staining has been performed once.

Supplemental Figure 33. IHC for HCI-050

Immunohistochemistry of HCI PDX line comparing the original patient tumor to the resulting PDX tumor. Stainings: H&E, ER, PR, HER2, CK-pan (Cytokeratin-pan), E-Cad (E-cadherin), hVim (human vimentin), msCD45, CK-CAM5.2 (human specific cytokeratin CAM5.2), hCD45, EpCAM, and hMito (human mitochondria). Each IHC staining has been performed once.

Supplemental Figure 34. IHC for HCI-051

Immunohistochemistry of HCI PDX line comparing the original patient tumor (as a cytospin) to the resulting PDX tumor. Stainings: H&E, ER, PR, HER2, CK-pan (Cytokeratin-pan), E-Cad (E-cadherin), hVim (human vimentin), msCD45, CK-CAM5.2 (human specific cytokeratin CAM5.2), hCD45, EpCAM, and hMito (human mitochondria). Each IHC staining has been performed once.

Supplemental Figure 35. IHC for HCI-052

Immunohistochemistry of HCI PDX line comparing the original patient tumor to the resulting PDX tumor. Stainings: H&E, ER, PR, HER2, CK-pan (Cytokeratin-pan), E-Cad (E-cadherin), hVim (human vimentin), msCD45, CK-CAM5.2 (human specific cytokeratin CAM5.2), hCD45, EpCAM, and hMito (human mitochondria). Each IHC staining has been performed once.

Supplemental Figure 36. IHC for HCI-053

Immunohistochemistry of HCI PDX line comparing the original patient tumor to the resulting PDX tumor. Stainings: H&E, ER, PR, HER2, CK-pan (Cytokeratin-pan), E-Cad (E-cadherin), hVim (human vimentin), msCD45, CK-CAM5.2 (human specific cytokeratin CAM5.2), hCD45, EpCAM, and hMito (human mitochondria). Each IHC staining has been performed once, and there was not enough patient tumor sample available for IHC stains.

Supplemental Figure 37. IHC for HCI-054

Immunohistochemistry of HCI PDX line comparing the original patient tumor to the resulting PDX tumor. Stainings: H&E, ER, PR, HER2, CK-pan (Cytokeratin-pan), E-Cad (E-cadherin), hVim (human vimentin), msCD45, CK-CAM5.2 (human specific cytokeratin CAM5.2), hCD45, EpCAM, and hMito (human mitochondria). Each IHC staining has been performed once.

Supplemental Figure 38. PDX subline HCI-028LV

(a) Image showing a breast cancer metastasis to mouse liver that was used to establish PDX subline HCI-028LV. **(b)** Immunohistochemistry of parental metastases and PDX line. Stainings: H&E, ER, PR, HER2, CK-pan (Cytokeratin-pan), E-Cad (E-cadherin), hVim (human vimentin), msCD45, CK-CAM5.2 (human-specific

cytokeratin CAM5.2), hCD45, EpCAM, and hMito (human mitochondria). Each IHC staining has been performed once for each PDX line.

Supplemental Figure 39. PDX subline HCI-031OV

(a) Image showing a breast cancer metastasis to mouse ovary that was used to establish PDX subline HCI-031OV. **(b)** Immunohistochemistry of parental metastases and PDX line. Stainings: H&E, ER, PR, HER2, CK-pan (Cytokeratin-pan), E-Cad (E-cadherin), hVim (human vimentin), msCD45, CK-CAM5.2 (human-specific cytokeratin CAM5.2), hCD45, EpCAM, and hMito (human mitochondria). Each IHC staining has been performed once for each PDX line.

Supplemental Figure 40. IHC for estrogen independent (-EI) PDX lines

Immunohistochemistry of estrogen-independent PDX HCI lines **(a)** HCI-013EI, **(b)** HCI-032, **(c)** HCI-040EI, and **(d)** HCI-044EI. Stainings: H&E, ER, PR, HER2, CK-CAM5.2 (Cytokeratin CAM5.2). “P” denotes passage number. Scale bar corresponds to 100 um. Each IHC staining has been performed once for each PDX line.

Supplemental Figure 41. PDxO Histology

Histology showing H&E stains of PDxO lines HCI-001, HCI-002, HCI-010, HCI-025 and HCI027. Stains of the parental PDX tumor or PDxOs cultured for 0, 60 or >120 days are shown. PDX tumors retain morphology when compared to re-implanted PDxoX after being cultured as PDxoX for 60 or 120 days. Scale bar corresponds to 50 um. Each IHC staining has been performed once for each PDX line.

Supplemental Figure 42. IHC for CK-CAM5.2 on PDxO lines

Immunohistochemistry showing CK-CAM5.2 stains for PDxO lines HCI-001, HCI-002, HCI-010, HCI-025 and HCI-027. Stains of the parental PDX tumor or PDxOs cultured for 0, 60 or >120 days are shown. Scale bar corresponds to 50 um. Each IHC staining has been performed once for each PDX line.

Supplemental Figure 43. IHC for Ki67 on PDxO lines

Immunohistochemistry showing Ki67 stains for PDxO lines HCI-001, HCI-002, HCI-010, HCI-025 and HCI-027. Stains of the parental PDX tumor or PDxOs cultured for 0, 60 or >120 days are shown. Scale bar corresponds to 50 um. Each IHC staining has been performed once for each PDX line.

Supplemental Figure 44. IHC for human vimentin on PDxO lines

Immunohistochemistry showing human vimentin stains for PDxO lines HCI-001, HCI-002, HCI-010, HCI-025 and HCI-027. Stains of the parental PDX tumor or PDxOs cultured for 0, 60 or >120 days are shown. Scale bar corresponds to 50 um. Each IHC staining has been performed once for each PDX line.

Supplemental Figure 45. FACS gating strategy

FACS sorting gates shown for **(a)** LDEV clean up and **(b)** PDxO mouse cell removal.
