# Supplementary material for: A human breast cancer-derived xenograft and organoid platform for drug discovery and precision oncology
Source: Nat Cancer. 2022 Feb 24;3(2):232–50. doi: 10.1038/s43018-022-00337-6 (PMC8882468; doi:10.1038/s43018-022-00337-6)
Supplement: Supplementary file 2 — Reporting Summary [file 43018_2022_337_MOESM2_ESM.pdf]

nature research

Corresponding author(s): Alana L. Welm, Bryan E. Welm

Last updated by author(s): January 11, 2022

Reporting Summary

Nature Research wishes to improve the reproducibility of the work that we publish. This form provides structure for consistency and transparency in reporting. For further information on Nature Research policies, see our Editorial Policies and the Editorial Policy Checklist.

Statistics

For all statistical analyses, confirm that the following items are present in the figure legend, table legend, main text, or Methods section.

n/a	Confirmed	
☐	☒	The exact sample size (*n*) for each experimental group/condition, given as a discrete number and unit of measurement
☐	☒	A statement on whether measurements were taken from distinct samples or whether the same sample was measured repeatedly
☐	☒	The statistical test(s) used AND whether they are one- or two-sided<br>*Only common tests should be described solely by name; describe more complex techniques in the Methods section.*
☒	☐	A description of all covariates tested
☒	☐	A description of any assumptions or corrections, such as tests of normality and adjustment for multiple comparisons
☐	☒	A full description of the statistical parameters including central tendency (e.g. means) or other basic estimates (e.g. regression coefficient) AND variation (e.g. standard deviation) or associated estimates of uncertainty (e.g. confidence intervals)
☐	☒	For null hypothesis testing, the test statistic (e.g. F, t, r) with confidence intervals, effect sizes, degrees of freedom and P value noted<br>*Give P values as exact values whenever suitable.*
☒	☐	For Bayesian analysis, information on the choice of priors and Markov chain Monte Carlo settings
☒	☐	For hierarchical and complex designs, identification of the appropriate level for tests and full reporting of outcomes
☐	☒	Estimates of effect sizes (e.g. Cohen's d, Pearson's r), indicating how they were calculated

Our web collection on statistics for biologists contains articles on many of the points above.

Software and code

Policy information about availability of computer code

Data collection	CellTiter Glo data were collected using PerkinElmer EnVision Manager v1.14 plate reader software. Confocal images were acquired with CellSens Imaging software from Olympus. IHC images were acquired on an Olympus BX50 microscope with UplanFI 20x/0.50 using a Canon EOS camera and acquisition software EOS Utility 2 ver.2. If brightness and/or saturation were adjusted it was applied to the entire image using Adobe Photoshop CC 2019.
Data analysis	Code used to parse, analyze, and visualize high-throughput genomics and drug screening data is publicly available at https://github.com/MHBailey/pdxo_2021_paper. DNA, RNA, and SNParray sequence alignment and variant calling were performed on the SevenBridges platform using PDXnet specific workflows. Code is available at the following URLs after acquiring CGC access permissions (https://cgc.sbgenomics.com/u/michael_lloyd/pdxnet-jax-wes-tumor-only-workflow/, https://cgc.sbgenomics.com/u/pdxnet/pdxnet-jax-rna-seq-workflow/, and https://cgc.sbgenomics.com/u/pdxnet/pdx-wf-commit2/apps/#pdxnet/pdx-wf-commit2/snp-array-tumor-only-workflow-for-illumina-infinium-omni-2-5-exo/3, respectively). Other data were analyzed using GraphPad Prism v9.2.0. Combenefit software was used to generate Loewe synergy plots of PDxO drug screening. Quantasoft Software (#1864011, Bio-Rad) was used for ddPCR analysis.

For manuscripts utilizing custom algorithms or software that are central to the research but not yet described in published literature, software must be made available to editors and reviewers. We strongly encourage code deposition in a community repository (e.g. GitHub). See the Nature Research guidelines for submitting code & software for further information.

Data

Policy information about availability of data

All manuscripts must include a data availability statement. This statement should provide the following information, where applicable:

- Accession codes, unique identifiers, or web links for publicly available datasets
- A list of figures that have associated raw data
- A description of any restrictions on data availability

Whole exome sequencing, SNP array CNV data, and RNA sequencing data reported in Figures 1 and 4 are available to authorized users in the NIH database of Genotypes and Phenotypes (dbGaP) repository under the accession number phs002479.v1.p1. Data from five patients are excluded for posting raw data due to IRB language permitting their use in research but not public dissemination of genomic data (HCI-025, HCI-026, HCI-027, HCI-031, and HCI-035). Access to data will be granted upon registration in the dbGaP system as an approved user with an eRA Commons account and completion of the online data access request process. Cell line and patient-derived model DNA methylation data are available at GEO under accession code GSE152202 and GSE186747, respectively. Raw data from the drug screen are available in the Source Data files accompanying the online version of the manuscript. Raw data for Figures 2-4, and 6-8 and Extended Data Figures 1, 3, 4, and 9 have also been provided as Source Data files. All other data supporting the findings of this study are available from the corresponding author on reasonable request.

Field-specific reporting

Please select the one below that is the best fit for your research. If you are not sure, read the appropriate sections before making your selection.

☒ Life sciences ☐ Behavioural & social sciences ☐ Ecological, evolutionary & environmental sciences

For a reference copy of the document with all sections, see nature.com/documents/nr-reporting-summary-flat.pdf

Life sciences study design

All studies must disclose on these points even when the disclosure is negative.

Sample size

No statistical methods were used to predetermine sample sizes; our sample sizes are similar to those reported in previous publications and by PDXNet consortium standards (Evrard et al Cancer Research 2020: DOI: 10.1158/0008-5472).

Data exclusions

No data was excluded, with a few exceptions where technical errors occurred during an experiment, rendering data unusable (e.g. a Matrigel dome detached).

Replication

Replicates for each experiment are described in the figure legends. If not specified, the experiment was performed once with the presented number of data points. All datapoints were distinct; no repeated measures were used.

Randomization

Mice were randomized to treatment groups. For organoid experiments, organoids were randomly aliquoted to wells for treatment.

Blinding

Investigators were not blinded to the experiment (e.g. treatment group) due to staffing shortages, but data were analyzed by multiple investigators, some of whom were blinded to the experiment.

Reporting for specific materials, systems and methods

We require information from authors about some types of materials, experimental systems and methods used in many studies. Here, indicate whether each material, system or method listed is relevant to your study. If you are not sure if a list item applies to your research, read the appropriate section before selecting a response.

Materials & experimental systems

n/a	Involved in the study	
☐	☒	Antibodies
☐	☒	Eukaryotic cell lines
☒	☐	Palaeontology and archaeology
☐	☒	Animals and other organisms
☐	☒	Human research participants
☒	☐	Clinical data
☒	☐	Dual use research of concern

Methods

n/a	Involved in the study	
☒	☐	ChIP-seq
☐	☒	Flow cytometry
☒	☐	MRI-based neuroimaging

Antibodies

Antibodies used

The list is too long to include here. All antibodies, dilutions, supplier name, catalog number, clone name and lot number, as applicable are listed in the Supp. Table referenced in the methods.

Validation	All antibodies are commercially available and were not validated independently, except by including appropriate positive and negative controls in each experiment.

Eukaryotic cell lines

Policy information about cell lines

Cell line source(s)	Cell lines were obtained from ATCC (MCF7, #HTB-22; MDA-MB-231, #HTB-26; MDA-MB-468, #HTB-132; T47D, #HTB-133) or Lonza (hMSC, #PT2501) and cultured as recommended. MDA-MB-361 was obtained from another lab and validated by IDEXX CellCheck 9-Human STR marker profiling.
Authentication	Most cell lines were purchased as authenticated lines from ATCC. MDA-MB-361 was obtained from another lab and validated by IDEXX CellCheck 9-Human STR marker profiling.
Mycoplasma contamination	Cell lines were not tested for mycoplasma contamination.
Commonly misidentified lines (See ICLAC register)	No commonly misidentified cell lines were used in the study.

Animals and other organisms

Policy information about studies involving animals; ARRIVE guidelines recommended for reporting animal research

Laboratory animals	Female immune-compromised mice (strains NSG (Jax stock #5557), NOD/SCID (Jax stock #1303), or NRG (Jax stock #7799) were used to generate PDX, typically at the age of 3-4 weeks. In rare cases, for urgent situations with younger mice not available or for ovariectomy experiments, we used mice up to 10 weeks of age. 8-10 week old male or female C57BL/6J mice (Jax stock #664) were used to grow EHS tumors. 3-4 week old female NIH-RNU rats (Charles River stock #568) were used to passage HCI-010 to rid it of LDEV. All animals were housed under standard, regulated conditions: Temperature range: 68 – 79 degrees<br>Humidity range: 30 – 70%, 12 hours light cycle (6am-6pm = On, 6pm-6am = Off).
Wild animals	No wild animals were used in the study.
Field-collected samples	No field-collected samples were used in the study.
Ethics oversight	All procedures using live animals were reviewed and approved by the University of Utah Institutional Animal Care and Use Committee (IACUC).

Note that full information on the approval of the study protocol must also be provided in the manuscript.

Human research participants

Policy information about studies involving human research participants

Population characteristics	Female breast cancer patients who were undergoing surgery or tumor biopsy as standard of care, or enrolled in the TOWARDS study, were eligible to donate a portion of the tumor that would otherwise be thrown away for research purposes. The age of the participants is listed in Supp. Table 2.
Recruitment	Patients who met the eligibility criteria were identified and prospectively accrued by their treating physicians at the outpatient clinics of Huntsman Cancer Institute. The treating physicians serve as co-investigators in the protocol and all patients signed informed consent to allow for tissue acquisition. The study was conducted in a single institution and, while reflective of the Utah patient population, every effort was undertaken for an as broad patient representation as possible. All research procedures were conducted in the context of other medically necessary procedures. Participants were not compensated.
Ethics oversight	The University of Utah Institutional Review Board (IRB protocols #89989, #91596 and #10924) approved human sample collection following informed consent. IRB#91596 allows us to return drug testing data to clinic, which was performed for one patient with written informed consent. The deidentified clinical information in this study is published in accordance to the ethics approvals for this study.

Note that full information on the approval of the study protocol must also be provided in the manuscript.

Flow Cytometry

Plots

Confirm that:

☒ The axis labels state the marker and fluorochrome used (e.g. CD4-FITC).

☒ The axis scales are clearly visible. Include numbers along axes only for bottom left plot of group (a 'group' is an analysis of identical markers).

☒ All plots are contour plots with outliers or pseudocolor plots.

☒ A numerical value for number of cells or percentage (with statistics) is provided.

Methodology

Sample preparation	FACS was performed for a) LDEV removal of PDX lines or b) mouse cell elimination from PDxO cultures. In both cases, single cell suspensions were prepared and after Fc receptor blockage, antibody stains were performed. Cells were washed and kept on ice and protected from light until acquisition. Control stains and unstained control cells were used to set gates. Cells were sorted using a 100um nozzle. In the case of a), cells were immediately implanted into mice for PDX tumor expansion. In the case of b), cells were immediately embedded into matrigel to re-initiate PDxO cultures.
Instrument	BD FACSAria II
Software	Diva version 8.0
Cell population abundance	Abundance of post-sort cell populations are reported in Suppl.Fig. 45 for representative experiments. Specifically, for LDEV removal the sorted sample population was 99.4% (P4) of the parent (P2) (Suppl.Fig. 45a). Purity was determined by re-analyzing a small volume of the sorted cell fraction P4, and was 99.8%. In the case were mouse cells were eliminated in the representative experiment in Suppl. Fig. 45b, the sorted human tumor cell population was 80.2% of the parental. Purity checks were performed similar to a).
Gating strategy	For a) LDEV removal of PDX lines, cells were gated on SSC-Area and FSC-Area for morphology to remove debris (P1). Cells in P1 were then gated on FSC-Area and FSC-Width to remove doublets and only cells in the singlet gate P2 were used for sorting. Cells were then gated on fluorophore intensity which represents expression of mouse cell marker CD45 to exclude potential mouse lymphoma cells and residual mouse immune cells (P6), and on human CD298 (P4). Only cells from gate P4 were collected and re-injected into mice to generate PDX.<br>In the case of b) mouse cell elimination from PDxO cultures, cells were gated on SSC-Area and FSC-Area for morphology to remove debris (P1). Cells in P1 were then gated on FSC-Area and FSC-Width to remove doublets and only cells in the singlet gate P2 were used in a second gating to remove doublets (P5) using SSC-Area and SSC-Width. Cells from P5 were then gated on fluorophore intensity which represents expression of mouse cell markers (P3) and human cell markers (P4). Sorted cells from P4 were collected and used for mouse cell eliminated organoid culure.<br>Gates that were set using FSC and SSC were determined by unstained control cells. Gates which were used to define positive populations based on fluorophore intensity were defined based on positive controls stained with a single fluorophore.

☒ Tick this box to confirm that a figure exemplifying the gating strategy is provided in the Supplementary Information.

April 2020
